# Pacsin 2-dependent N-cadherin internalization regulates the migration behaviour of malignant cancer cells

**DOI:** 10.1242/jcs.260827

**Published:** 2023-05-31

**Authors:** Haymar Wint, Jianzhen Li, Tadashi Abe, Hiroshi Yamada, Takumi Higaki, Yasutomo Nasu, Masami Watanabe, Kohji Takei, Tetsuya Takeda

**Affiliations:** ^1^ Department of Neuroscience, Okayama University Graduate School of Medicine, Dentistry and Pharmaceutical Sciences, Shikata-cho 2-5-1, Kita-ku, Okayama 700-8558, Japan; ^2^ Laboratory for Neural Cell Dynamics, RIKEN Center for Brain Science, Hirosawa 2-1, Wako, Saitama 351-0198, Japan; ^3^ Faculty of Advanced Science and Technology, Kumamoto University, Kurokami, Chuo-ku, Kumamoto 860-8555, Japan; ^4^ International Research Organization for Advanced Science and Technology, Kumamoto University, Kurokami, Chuo-ku, Kumamoto 860-8555, Japan; ^5^ Department of Urology, Okayama University Graduate School of Medicine, Dentistry and Pharmaceutical Sciences, Okayama 700-8558, Japan

**Keywords:** N-cadherin, Pacsin 2, Dynamin 2, Endocytosis, Collective cell migration

## Abstract

Collective cell migration is the coordinated movement of multiple cells connected by cadherin-based adherens junctions and is essential for physiological and pathological processes. Cadherins undergo dynamic intracellular trafficking, and their surface level is determined by a balance between endocytosis, recycling and degradation. However, the regulatory mechanism of cadherin turnover in collective cell migration remains elusive. In this study, we show that the Bin/amphiphysin/Rvs (BAR) domain protein pacsin 2 (protein kinase C and casein kinase substrate in neurons protein 2) plays an essential role in collective cell migration by regulating N-cadherin (also known as CDH2) endocytosis in human cancer cells. Pacsin 2-depleted cells formed cell–cell contacts enriched with N-cadherin and migrated in a directed manner. Furthermore, pacsin 2-depleted cells showed attenuated internalization of N-cadherin from the cell surface. Interestingly, GST pull-down assays demonstrated that the pacsin 2 SH3 domain binds to the cytoplasmic region of N-cadherin, and expression of an N-cadherin mutant defective in binding to pacsin 2 phenocopied pacsin 2 RNAi cells both in cell contact formation and N-cadherin endocytosis. These data support new insights into a novel endocytic route of N-cadherin in collective cell migration, highlighting pacsin 2 as a possible therapeutic target for cancer metastasis.

## Teaser

**Summary:** The BAR domain protein pacsin 2 plays an essential role in collective cell migration of cancer cells by regulating internalization of N-cadherin.

## INTRODUCTION

Cell migration is fundamental for diverse physiological and pathological processes, including development, immune responses and cancer metastasis (Yamada and Sixt, 2019). Cancer cells migrate either individually or collectively during metastasis (Pandya et al., 2017). Collectively migrating cancer cells are generally more aggressive and resistant to chemotherapies compared to individually migrating cancer cells (Aceto et al., 2014). Collective cell migration is a coordinated movement of a group of cells that are connected via adherens junctions (Friedl and Gilmour, 2009; Rørth, 2009). Different guidance mechanisms, such as chemotaxis, haptotaxis, durotaxis and strain-induced mechanosensing, are involved in the collective movement of cells (Haeger et al., 2015; Shellard and Mayor, 2021). For successful collective cell migration, two groups of cell adhesion molecules play essential roles in generating and coordinating mechanical forces among cells: focal adhesion (FA) molecules such as integrins, which transmit forces between cells and the underlying extracellular matrix (ECM), and adherens junction molecules such as cadherins, which transmit forces at intercellular adhesion sites (Halbleib and Nelson, 2006; Ray et al., 2017).

Cadherins are homophilic Ca^2+^-dependent cell adhesion molecules that play important roles in various physiological and pathological processes such as development (Gumbiner, 2005; Halbleib and Nelson, 2006) and cancer (Kaszak et al., 2020). There are over 100 different cadherin subtypes in vertebrates, and they can be classified into four groups: classical cadherins, desmosomal cadherins, protocadherins and unconventional cadherins (Yagi and Takeichi, 2000). From N- to C-terminus, each cadherin contains a large extracellular ectodomain followed by a transmembrane domain and a small cytoplasmic domain (Oda and Takeichi, 2011). Interactions between the ectodomains of cadherins from apposed cells mediate cell–cell contact, whereas the cytoplasmic domain contributes to linking cadherins to the underlying actin cytoskeleton by forming a complex with α- and β-catenins (Ratheesh and Yap, 2012). The cytoplasmic domain of cadherins also binds to p120 catenin (hereafter referred to as p120; also known as CTNND1), which controls endocytosis and turnover of cadherin, thus regulating cell surface cadherin levels responsible for cell–cell adhesion (Cadwell et al., 2016). A recent study has shown that classical cadherins – E-cadherin (also known as CDH1) and N-cadherin (also known as CDH2) – mediate cell–cell contacts to enhance the spreading efficiency of collectively migrating cells (Zisis et al., 2022). Another study on collectively migrating endothelial cells has shown that polarized membrane protrusions enriched with unconventional VE-cadherin (CDH5) called ‘cadherin fingers’ serve as guidance cues that direct collective cell migration (Hayer et al., 2016). Furthermore, classical P-cadherin (CDH3) enhances the collective cell migration of myoblasts by activating Cdc42, increasing the strength and anisotropy of mechanical forces (Plutoni et al., 2016). The cadherin-mediated cell–cell contact is determined by a balance between endocytosis, recycling and degradation (Akhtar and Hotchin, 2001; Cadwell et al., 2016; Kowalczyk and Nanes, 2012; Le et al., 1999). However, the regulatory mechanisms of cadherin turnover in collective cell migration remain to be elucidated.

Bin/amphiphysin/Rvs (BAR) domain proteins are a conserved family of proteins that possess the ability to sense membrane curvature and deform membranes (Peter et al., 2004; Safari and Suetsugu, 2012). BAR domain proteins play crucial roles in endocytosis (Takei et al., 1999), exocytosis (Pinheiro et al., 2014), cell migration (Sánchez-Barrena et al., 2012), cytokinesis (Takeda et al., 2013) and cancer metastasis (Yamamoto et al., 2011). BAR domains form ‘crescent-shaped’ dimers that are classified into three subtypes, each with distinctive topology and curvature: N-BAR (N-terminal amphipathic helix and BAR), F-BAR (Fes/CIP4 homology BAR) and I-BAR (inverse BAR) (Qualmann et al., 2011; Safari and Suetsugu, 2012). Pacsin (protein kinase C and casein kinase substrate in neurons protein; also known as synaptic dynamin-associated protein, syndapin) contains an F-BAR domain and an SH3 domain in its N- and C-termini, respectively (Dumont and Lehtonen, 2022). Three pacsin isoforms are expressed in mammalian cells: the neuronal isoform pacsin 1, the muscle-specific isoform pacsin 3 and the ubiquitously expressed isoform pacsin 2 (Modregger et al., 2000). Pacsin 2 has been implicated in caveolar endocytosis, vesicle trafficking and actin dynamics (Chandrasekaran et al., 2016; de Kreuk et al., 2011; Hansen et al., 2011; Senju et al., 2011). Pacsin 2 is also involved in the regulation of cell spreading and migration by associating with Rac1 (de Kreuk et al., 2011). Furthermore, based on TCGA PanCancer Atlas studies in cBioPortal (https://www.cbioportal.org/), deep deletions or mutations in the pacsin 2 gene that potentially cause gain or loss of function have been identified in samples from people with bladder cancer as well as other types of malignant cancers including ovarian and breast cancers (Cerami et al., 2012; Gao et al., 2013). Previous studies have shown that dynamin 2, a major pacsin 2-associated protein, is required for the internalization of E-cadherin (Miyashita and Ozawa, 2007; Paterson et al., 2003) and VE-cadherins (Chiasson et al., 2009). However, the requirement of pacsins in cadherin turnover remains elusive.

In this study, we show that pacsin 2 is involved in the collective cell migration of cancer cells by controlling N-cadherin internalization. Depletion of pacsin 2 in T24 bladder cancer cells and H1299 lung cancer cells induces cell–cell contacts enriched with N-cadherin. Electron microscopy shows that the cell–cell contacts induced by pacsin 2 depletion consist of interdigitating finger-like membranous protrusions. Imaging analyses of wound healing assays demonstrate that pacsin 2-depleted T24 cells exhibit directed cell migration. Furthermore, cell surface biotinylation and endocytosis assays show that N-cadherin internalization is inhibited in pacsin 2-depleted T24 cells. Interestingly, GST pull-down assays show that the SH3 domain of pacsin 2 binds to the cytoplasmic domain of N-cadherin, suggesting a direct role of pacsin 2 in regulating N-cadherin endocytosis. Indeed, expression of an N-cadherin mutant with defective pacsin 2 binding induced cell–cell contact formation and attenuated internalization, phenocopying pacsin 2 RNAi cells. These results suggest that pacsin 2 plays an essential role in regulating the endocytosis of N-cadherin, which affects the cell migration behaviour of malignant cancer cells.

## RESULTS

### Pacsin 2 localizes at the cell periphery in T24 cells

**Fig. 1.**
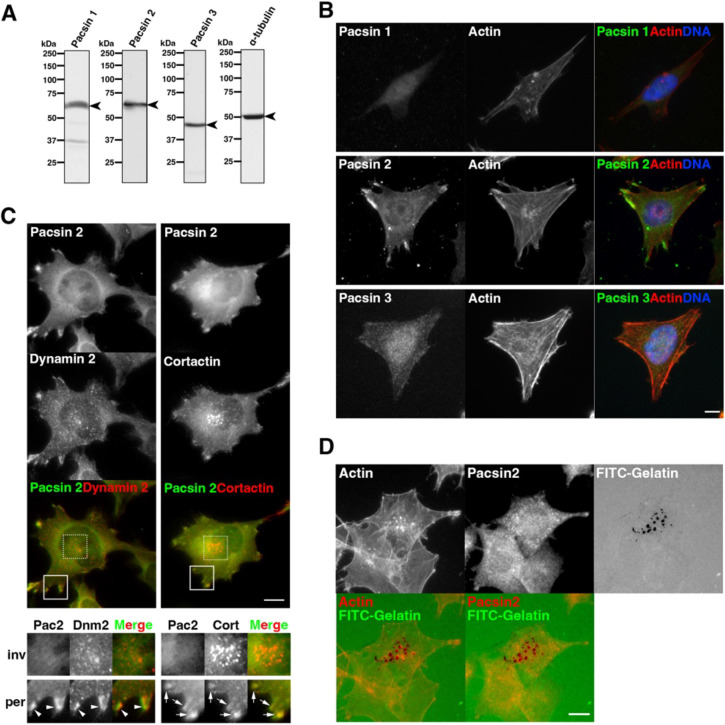
**Pacsin 2 localises to the cell periphery in T24 cells.** (A) Immunoblot analyses of endogenous pacsin 1, pacsin 2, pacsin 3 and α-tubulin (arrowheads) in T24 cells. Blots shown are representative of three independent experiments. (B) Localization of endogenous pacsin 1, pacsin 2 and pacsin 3, and of F-actin. Merged images show pacsins (green), F-actin (red) and DNA (blue). (C) Immunofluorescence microscopy images of endogenous pacsin 2 (green) with either endogenous dynamin 2 (red) or endogenous cortactin (red), as indicated. Perinuclear (dashed box) and peripheral (solid box) regions are shown as enlarged images in the lower panel (inv and per, respectively). Colocalization of pacsin 2 (Pac2) with either dynamin 2 (Dnm2, arrowheads) or cortactin (Cort, arrows) in peripheral regions can be observed in the enlarged images. (D) Localization of pacsin 2 (red) or F-actin (red, pseudocolour), as indicated, with FITC–gelatin (green) in T24 cells. Images in B–D are representative of *n*≥105 cells from three independent experiments. Scale bars: 10 μm.

To determine the functions of pacsins in cancer cells, the expression and localization profiles of pacsin isoforms were examined in T24 cells. Immunoblot analysis of whole-cell extract from T24 cells revealed that all the pacsin isoforms were expressed in T24 cells (Fig. 1A). Immunofluorescence microscopy showed that pacsin 2 was concentrated at the cell periphery in T24 cells, whereas pacsin 1 and pacsin 3 dispersedly localized in the cytoplasm (Fig. 1B). Pacsin 2 interacts with dynamin 2, which is required for the formation of invadopodia in T24 cells (Zhang et al., 2016). However, pacsin 2 did not colocalize with dynamin 2 at the perinuclear invadopodia, but the two proteins did colocalize at the cell periphery (Fig. 1C). Similarly, pacsin 2 also colocalized with the essential actin organizer cortactin (CTTN) at the cell periphery, but they were not colocalized at invadopodia (Fig. 1C). Furthermore, a degradation assay using FITC–gelatin confirmed that pacsin 2 does not localize to the degradation-competent invadopodia in perinuclear regions (Fig. 1D). These results suggest that, unlike dynamin 2, pacsin 2 is not involved in invadopodia formation but instead plays a role in processes at the cell periphery, such as cell migration.

### Pacsin 2 depletion induces directional migration of T24 cells

**Fig. 2.**
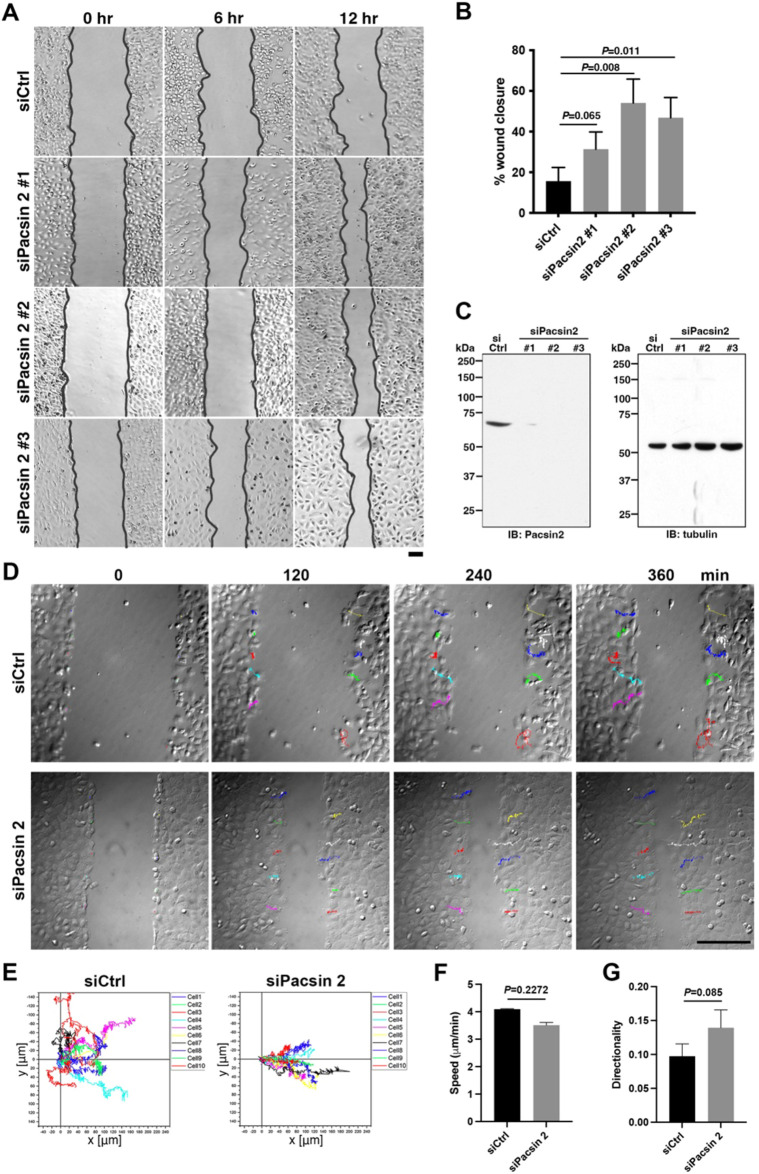
**Pacsin 2 depletion induces directional cell migration in T24 cells.** (A) Differential interference contrast microscopy images of migrating cells in the wound healing assay. Representative micrographs show either control RNAi cells (siCtrl) or pacsin 2 RNAi cells (siPacsin 2 #1, #2 and #3) at 0, 6 and 12 h after the start of the wound healing assay. Black lines indicate the wound edges. Scale bar: 200 μm. (B) Quantitation of wound closure by either control RNAi cells (siCtrl) or pacsin 2 RNAi cells (siPacsin 2 #1, #2 and #3). Data are means±s.d. of three independent experiments, five areas each. *P*-values were calculated using an unpaired two-tailed *t*-test. (C) Immunoblot analysis of cell extract from control RNAi cells (siCtrl) and pacsin 2 RNAi cells (siPacsin 2 #1, #2 and #3) using antibodies against pacsin 2 (IB: Pacsin 2) or tubulin as an internal control (IB: tubulin). Blots shown are representative of three independent experiments. (D) Live-cell imaging analysis of the wound healing assay. Time-lapse images of control RNAi (siCtrl) or pacsin 2 RNAi (siPacsin 2) cells at 0, 120, 240 and 360 min after the start of the wound healing assay. Traced paths of ten representative cells are shown in different colours. Scale bar: 100 μm. (E) Trajectories of cell tracking for representative cells over 360 min. The position of each cell at 0 min was set as the origin, and tracks were aligned so that positive *x* displacement values were towards the centre of the wound. (F) Quantitation of cell speed in the wound healing assay. Data are mean±s.d. (*n*=10 cells, *N*=3) in 360 min of the wounding healing assay. (G) Quantitative analysis of cell directionality in the wound healing assay. Data are mean±s.d. (*n*=10 cells, *N*=3) in 360 min of the wounding healing assay. *P*-values in F and G were calculated using an unpaired two-tailed *t*-test.

To elucidate whether pacsin 2 is involved in the migration of T24 cells, the effect of pacsin 2 depletion was examined in a wound healing assay. Control RNAi T24 cells (treated with a non-targeting siRNA, siCtrl) migrated slowly, and only 15.7% of the scratched area was filled after 12 h (Fig. 2A,B, siCtrl). In contrast, pacsin 2 RNAi cells showed enhanced migration activity, and the wound closure area was extended to 31.3–54.1% in 12 h (Fig. 2A,B, siPacsin 2 #1, #2 and #3). Immunoblot analyses confirmed that all three different siRNAs targeting pacsin 2 caused depletion of pacsin 2 (Fig. 2C). These results suggest that pacsin 2 negatively regulates cell migration activities of T24 cells.

To clarify the cause of the enhanced cell migration exhibited by pacsin 2 RNAi cells, the dynamics of cell migration were analysed by live-cell imaging in the wound healing assay. Tracking of representative cells showed that control RNAi cells moved with an average speed of 4.1 μm/min, but they moved randomly (Fig. 2D–G, siCtrl; Movie 1). In contrast, pacsin 2 RNAi cells migrated in a more directed manner, though their speed was comparable to that of control RNAi cells (3.5 μm/min) (Fig. 2D–G, siPacsin 2; Movie 2). These results suggest that pacsin 2 has a role in regulating the directionality of cell migration.

### Pacsin 2 depletion induces cell–cell contacts enriched with N-cadherin

**Fig. 3.**
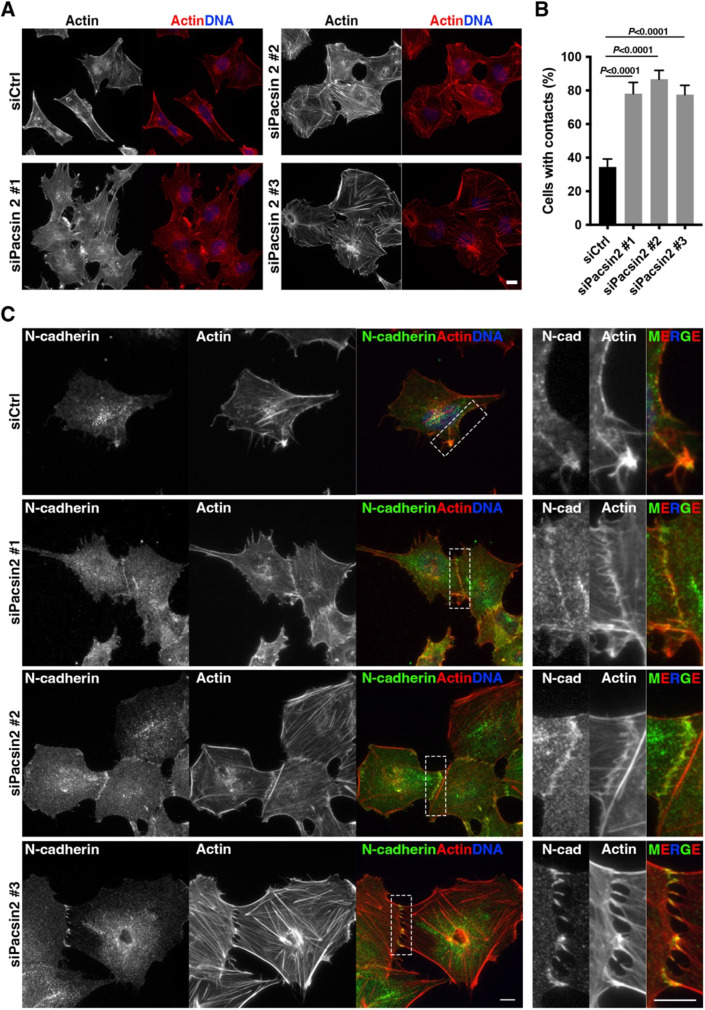
**Pacsin 2 depletion induces formation of N-cadherin-rich cell–cell contacts in T24 cells.** (A) Immunofluorescence micrographs of control RNAi cells (siCtrl) and pacsin 2 RNAi cells (siPacsin 2 #1, #2 and #3) stained for F-actin. Merged images show F-actin (red) with DNA (blue). Scale bar: 10 μm. (B) Quantitation of the percentage of cells with cell contacts in control RNAi cells (siCtrl) and pacsin 2 RNAi cells (siPacsin 2 #1, #2 and #3). Data are mean±s.d. (*n*≥120 cells, *N*=3). *P*-values were calculated using an unpaired two-tailed *t*-test. (C) Immunofluorescence micrographs of control RNAi cells (siCtrl) and pacsin 2 RNAi cells (siPacsin 2 #1, #2 and #3) stained for endogenous N-cadherin (green), F-actin (red) and DNA (blue). Dashed boxes mark regions of the cell periphery in control cells or N-cadherin-rich cell–cell contact sites in pacsin 2 RNAi cells, and they are shown as enlarged images (right). Images are representative of *n*≥150 cells from three independent experiments. Scale bars: 10 μm.

To elucidate how pacsin 2 can affect the directionality of cell migration, cellular phenotypes were analysed using immunofluorescence microscopy. Control RNAi cells tended to grow individually, and only 34.5% of cells formed cell–cell contacts at subconfluent cell densities (Fig. 3A,B, siCtrl). In contrast, pacsin 2 RNAi induced cell clustering, and more than 77.5% of cells exhibited cell–cell contacts (Fig. 3A,B, siPacsin 2 #1, #2 and #3). Similarly, cell cluster formation was also induced by dynamin 2 RNAi (55.3%), whereas only 26.4% of control RNAi cells exhibited cell-cell contacts (Fig. S1A,B). These results suggest that pacsin 2 and dynamin 2 are involved in the formation of cell–cell contacts in T24 cells.

To identify the molecular components of the cell–cell contacts induced following RNAi of pacsin 2 or dynamin 2, the expression and localization profiles of cadherins were examined. Immunoblot analyses showed that RT4 cells, which represent papillary bladder carcinoma, expressed E-cadherin but not N-cadherin, whereas T24 cells, which represent more aggressive bladder cancer, expressed N-cadherin but not E-cadherin (Fig. S2A). In contrast, neither classical P-cadherin nor unconventional VE-cadherin were expressed in T24 cells (Fig. S2A). To determine whether N-cadherin is a component of the cell–cell contacts induced following RNAi of pacsin 2 or dynamin 2, the localization of N-cadherin in T24 cells was analysed by immunofluorescence microscopy. Endogenous N-cadherin colocalized with pacsin 2 and dynamin 2 at the cell periphery in T24 cells (Fig. S2B). Similarly, N-cadherin localized at the cell periphery, as well as in cytoplasmic dots, in control RNAi cells (Fig. 3C, siCtrl). In contrast, in pacsin 2 RNAi cells, N-cadherin accumulated at cell–cell contact sites where actin filaments from contacting cells were interdigitated (Fig. 3C, siPacsin 2 #1, #2 and #3). To probe the relationship between depletion of pacsin 2 and formation of cell–cell contacts more robustly and transparently, we captured low magnification images of the formation of cell–cell contacts by pacsin 2 RNAi cells (Fig. S3). Furthermore, to assess the effects of pacsin 2 depletion on N-cadherin localization at junctions, densely plated control and pacsin 2 RNAi cells were stained for N-cadherin and pacsin 2 (Fig. S4A,B). A similar distribution of N-cadherin to the cell–cell contact sites was also observed in dynamin 2 RNAi cells (Fig. S1C), suggesting a functional association between pacsin 2 and dynamin 2 in the formation of N-cadherin-rich cell–cell contacts.

To determine whether pacsin 2 has conserved roles in the induction of N-cadherin-rich cell junctions, its function was also analysed using the human non-small lung carcinoma cell lines A549 and H1299. Immunoblot analyses showed that H1299 cells, but not A549 cells, have a cadherin expression profile similar to that of T24 cells: N-cadherin expression was detected, but expression of E-, P- and VE-cadherins was not detected (Fig. S5A). Pacsin 2 was also expressed in H1299 cells (Fig. S5B) and colocalized with N-cadherin at the cell periphery (Fig. S5C). As shown in immunoblot analysis, pacsin 2 was efficiently depleted by RNAi in H1299 cells (Fig. S5D). Importantly, depletion of pacsin 2 in H1299 cells also induced cell–cell contacts enriched with N-cadherin (Fig. S5E,F). To probe the relationship between pacsin 2 depletion and cell–cell contact formation more transparently, we captured low magnification views of the formation of cell–cell contacts by pacsin 2 RNAi cells (Fig. S5G). These results suggest that pacsin 2 has a conserved role in the formation of cell junctions at least within the context of a cancer cell line that expresses only N-cadherin.

**Fig. 4.**
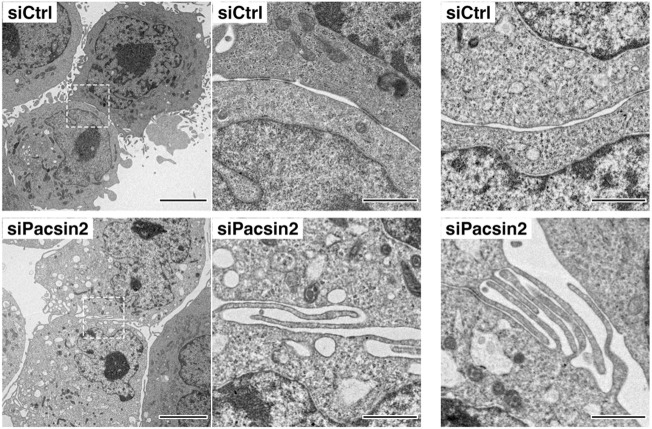
**Pacsin 2-depleted cells form interdigitating membrane protrusions at cell–cell contact sites in T24 cells.** Transmission electron microscopy images of cell–cell contact sites in control RNAi T24 cells (siCtrl) and pacsin 2 RNAi T24 cells (siPacsin 2) at different magnifications (700×, left; 4000×, middle and right). Dashed boxes indicate regions shown at higher magnification in the middle panels. Images are representative of *n*≥48 contacts imaged. Scale bars: 5 μm (left), 1 μm (middle and right).

To gain further insights into the cell–cell contact sites induced by pacsin 2 RNAi, their ultrastructure was analysed using electron microscopy. Control RNAi cells sometimes formed cell–cell contacts, but structures of the plasma membrane between closely apposed cells were smooth (Fig. 4, siCtrl). In contrast, in pacsin 2 RNAi cells, numerous membranous protrusions were formed at the cell–cell contact sites, and these protrusions were often interdigitating (Fig. 4, siPacsin 2). Immunoblot analyses showed that the total amount of N-cadherin was not altered in pacsin 2 RNAi cells and dynamin 2 RNAi cells (Fig. S6), suggesting that the cell surface level of N-cadherin, but not its transcription and/or translation, is regulated by pacsin 2 and dynamin 2 in T24 cells to induce cell–cell contacts.

### Depletion of pacsin 2 attenuates N-cadherin endocytosis in T24 cells

**Fig. 5.**
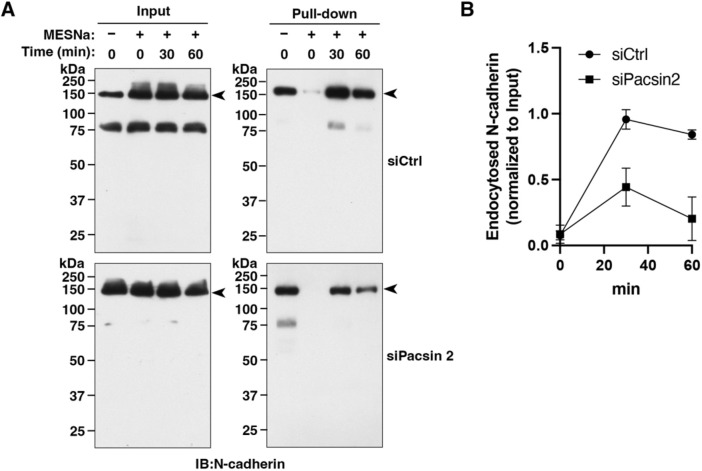
**Pacsin 2 is required for the internalization of N-cadherin.** (A) Representative immunoblots of N-cadherin internalization experiments. Surface N-cadherin in control RNAi T24 cells (siCtrl) and pacsin 2 RNAi T24 cells (siPacsin 2) was biotinylated at 4°C, and cells were subsequently incubated at 37°C for the indicated periods to allow endocytosis. MESNa was added, as indicated, to remove biotin remaining at the cell surface. Immunoblots (IB) for total N-cadherin (Input, arrowheads) and internalized N-cadherin (Pull-down, arrowheads) are shown. (B) Quantification of internalized N-cadherin in control RNAi cells (siCtrl) and Pacsin 2 RNAi cells (siPacsin 2) after normalizing the internalized N-cadherin (Pull-down) to the total amount of N-cadherin (Input) in experiments as shown in A. Data are mean±s.d. (*n*=3).

The cell surface level of cadherin is determined by a balance between endocytosis, recycling and degradation. Since both pacsin 2 and dynamin 2 have been implicated in endocytosis, we analysed the internalization of surface N-cadherin using a surface biotinylation and endocytosis assay. In both control and pacsin 2 RNAi cells, N-cadherin on the cell surface was internalized within 30 min, and the overall level of internalized N-cadherin then gradually decreased, probably due to degradation (Fig. 5A,B). However, in pacsin 2 RNAi cells, internalization of N-cadherin was attenuated, and the relative amount of internalized N-cadherin was ∼46.4% and ∼24.2% of that in the control cells at 30 min and 60 min, respectively, after the restart of endocytosis (Fig. 5A,B).

**Fig. 6.**
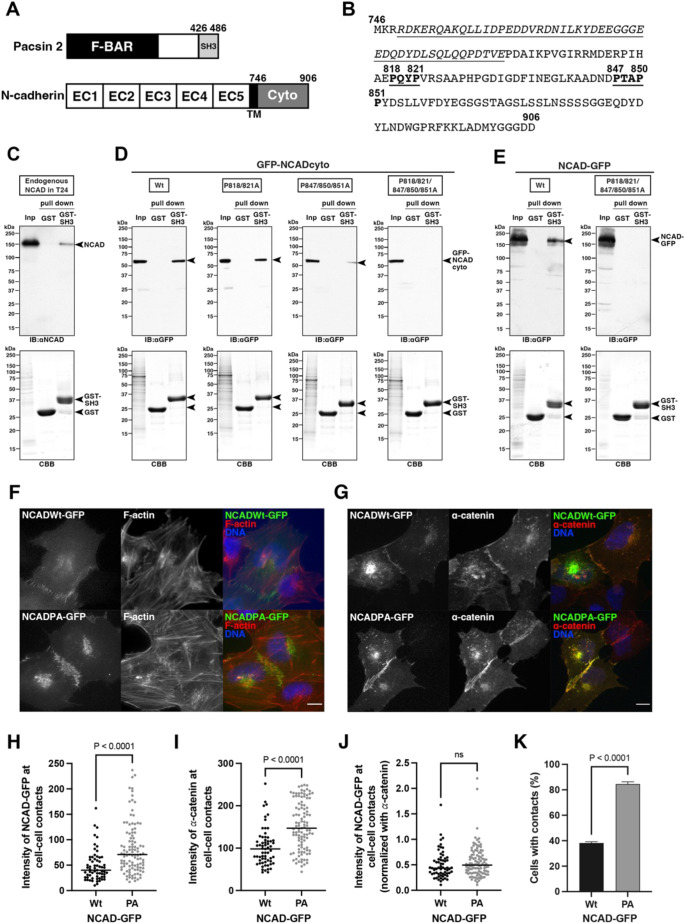
**Pacsin 2 SH3 domain interacts with the N-cadherin cytoplasmic region to regulate cell–cell contact formation.** (A) Schematically illustrated domain structures of human pacsin 2 and human N-cadherin. Cyto, cytoplasmic domain; EC, extracellular domain; TM, transmembrane domain. Numbers indicate amino acid positions. (B) Amino acid sequences of the cytoplasmic domain of N-cadherin. Two PxxP motifs (bold and underlined) and p120-binding regions (italicized and underlined) are shown. Numbers indicate amino acid positions. (C) Interaction between pacsin 2 SH3 domain and N-cadherin in T24 cells in GST pull-down assays. Immunoblot probed with anti-N-cadherin antibody (IB: αNCAD) and a CBB-stained SDS–PAGE gel (CBB) for input (Inp; 0.16% of total lysate) and pulled-down fractions with either GST beads (GST) or GST–pacsin 2 SH3 beads (GST–SH3) are shown. The positions of endogenous N-cadherin in T24 cells, and of GST–SH3 and GST alone, are marked by arrowheads. (D) Interaction between pacsin 2 SH3 domain and N-cadherin cytoplasmic domain in GST pull-down assays. Immunoblots probed with anti-GFP antibody (IB: αGFP) and CBB-stained SDS–PAGE gels (CBB) for input (Inp; 0.16% of total lysate) and pulled-down fractions with either GST beads (GST) or GST–pacsin 2 SH3 beads (GST–SH3) are shown. Positions of wild-type (wt) and mutant (P818/821A, P847/850/851A and P818/821/847/850/851A) forms of GFP-tagged N-cadherin cytoplasmic domain (GFP–NCADcyto), and of GST (GST) and GST-tagged pacsin 2 SH3 domain (GST–SH3), are marked by arrowheads. (E) Interaction between pacsin 2 SH3 domain and full-length N-cadherin in GST pull-down assays. Immunoblots probed with anti-GFP antibody (IB: αGFP) and CBB-stained SDS–PAGE gels (CBB) for input (Inp; 0.16% of total lysate) and pulled-down fractions with either GST beads (GST) or GST–pacsin 2 SH3 beads (GST–SH3) are shown. Positions of wild-type (Wt) or the PxxP mutant form (P818/821/847/850/851A) of GFP-tagged N-cadherin (NCAD–GFP), and of GST (GST) and GST-tagged pacsin 2 SH3 domain (GST–SH3), are marked by arrowheads. Images shown in C–E are representative of three independent experiments. (F) Expression of pacsin 2-binding-defective N-cadherin phenocopies the effects of pacsin 2 depletion. Immunofluorescence images showing exogenously expressed GFP-tagged wild-type (NCADWt–GFP) or PA mutant (P818/821/847/850/851A mutations; NCADPA–GFP) N-cadherin, and F-actin in T24 cells. Merged images show GFP-tagged N-cadherin (green), F-actin (red) and DNA (blue). Scale bar: 10 μm. (G) Expression of pacsin 2-binding-defective N-cadherin induces accumulations of a junctional component. Immunofluorescence images showing exogenously expressed GFP-tagged wild-type (NCADWt–GFP) or PA mutant (NCADPA–GFP) N-cadherin, and α-catenin in T24 cells. Merged images show GFP-tagged N-cadherin (green), α-catenin (red) and DNA (blue). Scale bar: 10 μm. (H) Quantification of GFP-tagged N-cadherin (NCAD–GFP) signal intensity at cell–cell contact sites for wild-type (Wt) and PA mutant (PA) (Wt, *n*=63; PA, *n*=105). (I) Quantification of α-catenin signal intensity at cell–cell contact sites in cells expressing either wild-type (Wt) or PA mutant (PA) NCAD–GFP (Wt, *n*=63; PA, *n*=105). (J) The relative intensity of either wild-type (Wt) or PA mutant (PA) NCAD–GFP at cell–cell contact sites, normalized to α-catenin intensity (Wt, *n*=63; PA, *n*=105). Horizontal lines in H–J indicate the median. *P*-values in H–J were calculated using a two-tailed Mann–Whitney test (ns, not significant). (K) Quantification of cell contact formation upon expression of either wild-type (Wt) or PA mutant (PA) NCAD–GFP. Data are presented as mean±s.d. (*n*≥110 cells, *N*=3). *P*-values were calculated using an unpaired two-tailed *t*-test.

To address whether pacsin 2 plays a direct role in regulating N-cadherin endocytosis, the interaction between pacsin 2 and N-cadherin was examined using a GST pull-down assay. Pacsin 2 contains a C-terminal SH3 domain (Fig. 6A), which binds to proline-rich motifs in its interacting proteins (Dumont and Lehtonen, 2022). Interestingly, the cytoplasmic domain of N-cadherin contains two PxxP motifs (where x indicates any amino acid), which potentially bind to the SH3 domain of pacsin 2 (Fig. 6A,B). Indeed, endogenous N-cadherin in T24 cells bound to GST-tagged pacsin 2 SH3 domain, but not to GST alone (Fig. 6C). Similarly, both GFP-tagged N-cadherin cytoplasmic domain and GFP-tagged full-length N-cadherin bound to GST-tagged pacsin 2 SH3 domain, but not to GST alone (Fig. 6D,E, Wt). In contrast, the interaction between GST-tagged pacsin 2 SH3 domain and GFP-tagged N-cadherin cytoplasmic domain with proline-to-alanine substitutions in the PxxP motifs was reduced (Fig. 6D, P818/821A and P847/850/851A). Interestingly, the interaction was almost undetectable when GFP-tagged cytoplasmic domain or full-length N-cadherin containing mutations in both PxxP motifs was used in the GST pull-down assay (Fig. 6D,E, P818/821/847/850/851A). These results strongly suggest that pacsin 2 SH3 domain binds to the cytoplasmic domain of N-cadherin via both PxxP motifs to regulate N-cadherin internalization.

**Fig. 7.**
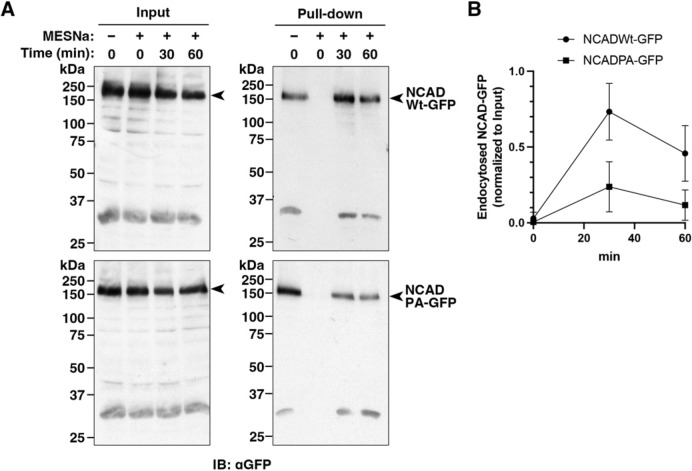
**A N-cadherin mutant with defective pacsin 2 binding shows attenuated internalization.** (A) Representative immunoblots of N-cadherin internalization experiments. Surface GFP-tagged N-cadherin was biotinylated at 4°C, and cells were subsequently incubated at 37°C for the indicated periods to allow endocytosis. MESNa was added, as indicated, to remove biotin remaining at the cell surface. Immunoblots using an anti-GFP antibody (IB: αGFP) to detect total GFP-tagged N-cadherin (Input, arrowheads) and internalized GFP-tagged N-cadherin (Pull-down, arrowheads) from T24 cells expressing either wild-type (NCADWt–GFP) or PA mutant (NCADPA–GFP) N-cadherin are shown. (B) Quantification of internalized GFP-tagged N-cadherin from cells expressing either wild-type (NCADWt–GFP) or PA mutant (NCADPA–GFP) N-cadherin after normalizing the internalized GFP-tagged N-cadherin (Pull-down) to the total amount of GFP-tagged N-cadherin (Input) in experiments as shown in A. Data are mean±s.d. (*n*=3).

We next determined whether expression of the proline-to-alanine mutant form of full-length N-cadherin (P818/821/847/850/851A, referred to hereafter as PA mutant) phenocopies the effects of pacsin 2 depletion. In T24 cells, exogenously expressed GFP-tagged full-length wild-type N-cadherin weakly accumulated at cell–cell contact sites together with another junctional component, α-catenin (herein referring to α-catenins in general), where interdigitating F-actin structures were rarely formed (Fig. 6F,G, NCADWt–GFP). In contrast, exogenously expressed GFP-tagged PA mutant N-cadherin localized to the cell–cell contact sites more robustly, together with α-catenin, often inducing interdigitated F-actin structures (Fig. 6F,G, NCADPA–GFP). Quantitative analyses showed that the signal intensities of GFP-tagged N-cadherin and α-catenin at cell–cell contact sites were slightly higher in cells expressing PA mutant N-cadherin–GFP than in cells expressing wild-type N-cadherin–GFP, and this difference was found to be statistically significant (Fig. 6H,I). Importantly, the relative intensities of N-cadherin–GFP normalized to α-catenin were equivalent between wild-type and PA mutants, excluding potential effects of differences in overexpression (Fig. 6J). Consistently, more than 80% of cells formed cell–cell contacts when the N-cadherin PA mutant was expressed, whereas only ∼30% of cells showed cell–cell contacts when expressing wild-type N-cadherin (Fig. 6K). Finally, surface biotinylation and endocytosis assays showed that internalization of the N-cadherin PA mutant was attenuated, and the relative amount of internalized N-cadherin was ∼32.5% and ∼25.5% of the amount of wild-type N-cadherin at 30 min and 60 min, respectively, after the restart of endocytosis (Fig. 7A,B). These results strongly suggest that pacsin 2 interacts with N-cadherin to mediate the N-cadherin internalization required for regulation of collective cell migration of T24 cells.

### Depletion of pacsin 2 and dynamin 2 enhances focal adhesion formation

**Fig. 8.**
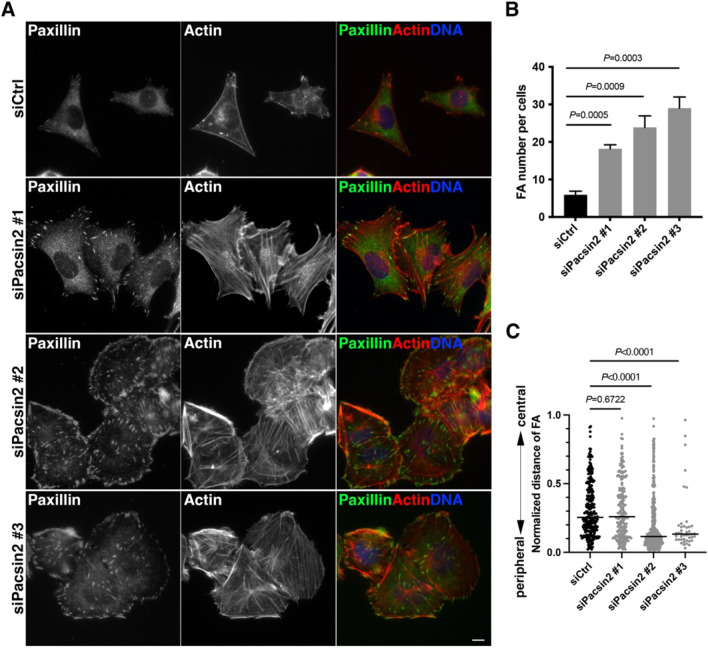
**Depletion of pacsin 2 induces an elevated number of FAs in T24 cells.** (A) Immunofluorescence micrographs of control RNAi cells (siCtrl) and pacsin 2 RNAi cells (siPacsin 2 #1, #2 and #3) stained for the FA marker paxillin and F-actin. Merged images show paxillin (green), F-actin (red) and DNA (blue). Scale bar: 10 μm. (B) Quantitation of FAs per cell in control RNAi cells (siCtrl) and pacsin 2 RNAi cells (siPacsin 2 #1, #2 and #3). Data are means±s.d. (*n*≥100 cells, *N*=3). *P*-values were calculated using an unpaired two-tailed *t*-test. (C) Spatial distribution of FAs in either control RNAi (siCtrl) or pacsin 2 RNAi (siPacsin 2 #1, #2 and #3) cells. Normalized positions of FAs between the cell periphery (0) and cell centre (1) are shown (control, *n*=272; siPacsin 2 #1, *n*=202; siPacsin 2 #2, *n*= 379; siPacsin 2 #3, *n*=43). Horizontal lines mark the median. *P*-values were calculated using a two-tailed Mann–Whitney test.

Collective cell migration requires not only cell–cell adhesion but also integrin-based FAs. A previous study has shown that dynamin 2 is required for the internalization of integrins in NIH-3T3 cells (Ezratty et al., 2005). Consistent with this, dynamin 2 RNAi also induced an increase in the number of FA sites in T24 cells (mean of 19.8 FA sites per cell) compared to the number in control RNAi cells (mean of 3.7 FA sites per cell) (Fig. S7A,B). Similar to the effects of dynamin 2 RNAi, immunofluorescence microscopy showed that the number of paxillin-positive FAs (18.2–29.0 FA sites per cell) exhibited by pacsin 2 RNAi cells was more than three times the number observed in control RNAi cells (5.9 FA sites per cell) (Fig. 8A,B). Quantification analyses showed that FAs are preferentially formed in peripheral regions in both pacsin 2 RNAi (Fig. 8C) and dynamin 2 RNAi cells (Fig. S7C). Increased FA numbers in pacsin 2 RNAi cells and dynamin 2 RNAi cells were also confirmed in single-cell conditions (Fig. S8). These results suggest that pacsin 2 and dynamin 2 are involved in the formation of FAs as well as cell–cell contacts that are essential for collective cell migration.

## DISCUSSION

In this study, we identified pacsin 2 as a novel regulator of collective cell migration in various cancer cell lines. Pacsin 2-depleted T24 cells migrated in a directional manner (Fig. 2) with an increased number of cell–cell contacts enriched with N-cadherin (Fig. 3). Similarly, pacsin 2 depletion also induced N-cadherin-rich cell junctions in the lung cancer cell line H1299 (Fig. S5), suggesting that pacsin 2 plays a conserved role as a negative regulator in the formation of N-cadherin-rich cell junctions. In many epithelial cancers, metastasis is facilitated by epithelial-to-mesenchymal transition (EMT) (Nieto et al., 2016). In the EMT, expression profiles of cadherin isoforms are typically switched from E-cadherin to N-cadherin in a process referred to as ‘cadherin switching’, which is associated with increased migratory and invasive behaviour of cancer cells (Wheelock et al., 2008). Previous studies have shown that N-cadherin promotes cell aggregation and collective invasion into collagen matrices, and penetration into mesenchymal layers in lung cancer (Kuriyama et al., 2016) and ovarian cancer (Klymenko et al., 2017). Likewise, in transformed Madin–Darby canine kidney (MDCK) cells, N-cadherin-mediated cell–cell adhesion enhances directional collective cell migration into 3D matrices (Shih and Yamada, 2012). Consistent with this, E-cadherin and N-cadherin were the dominant cadherin isoforms in less aggressive (RT4 and A549) and malignant (T24 and H1299) cancer cells, respectively (Figs S2, S5) (Elie-Caille et al., 2020; Mishra et al., 2018). Cadherin switching is controlled by either transcriptional (Maeda et al., 2005; Thiery and Sleeman, 2006) or post-transcriptional mechanisms (Davis et al., 2003; Ireton et al., 2002; Xiao et al., 2003a). In T24 cells, pacsin 2 RNAi and dynamin 2 RNAi both altered the surface level of N-cadherin without affecting its total expression level (Fig. S6), suggesting that pacsin 2, as well as dynamin 2, regulates N-cadherin internalization in T24 cells to affect their migratory behaviours.

In this study, we showed that interdigitating membranous protrusions are formed at the N-cadherin-rich cell–cell contact sites in pacsin 2 RNAi cells (Fig. 4). In human umbilical vein endothelial cells (HUVECs), VE-cadherin-rich membrane protrusions, known as cadherin fingers or focal adherens junctions (hereafter referred to as FAJs), serve as structural guidance cues that allow coordinated movement of collectively migrating cells (Dorland et al., 2016; Hayer et al., 2016). FAJs are asymmetric junctional structures that extend from the rear of leader cells and are engulfed at the front of follower cells, exposing topologically opposite membrane curvature to the cytoplasm of the leader cell (negatively curved) and the follower cell (positively curved). The distinct types of membrane curvature in FAJs are capable of selectively recruiting curvature-sensing BAR domain proteins. Indeed, the F-BAR domain protein pacsin 2 is recruited to a subset of FAJs, where it plays an important role to maintain cell–cell adhesion (Dorland et al., 2016). Interestingly, our study showed that pacsin 2 has the opposite effect on the formation of FAJ-like structures in T24 cells (Figs 3 and 4) and H1299 cells (Fig. S5). However, these results are not mutually exclusive, since multiple BAR domain proteins, such as AMPH1 or nostrin, may contribute to maintaining cell–cell adhesions at FAJs (Dorland et al., 2016; Hayer et al., 2016). Comprehensive analyses of various BAR domain proteins in cell–cell adhesion might reveal their cooperative functions in the formation and/or maintenance of FAJs during collective cell migration.

Pacsin 2 colocalized with N-cadherin at the cell periphery in T24 cells (Fig. S2B) and H1299 cells (Fig. S5C). Furthermore, pacsin 2 RNAi induced attenuation of N-cadherin internalization in T24 cells (Fig. 5), suggesting that pacsin 2 is required for N-cadherin endocytosis. Previous studies have shown that endocytosis of cadherins from the cell surface occurs in either a clathrin-dependent or clathrin-independent manner (Cadwell et al., 2016). In MDCK cells, E-cadherin is constitutively retrieved by clathrin-dependent endocytosis (Le et al., 1999). VE-cadherin in endothelial cells is also endocytosed in a clathrin-dependent manner, resulting in degradation in lysosomes (Xiao et al., 2003b). Similarly, N-cadherin is endocytosed in the clathrin-dependent pathway to facilitate neurite outgrowth (Chen and Tai, 2017). Furthermore, clathrin-dependent endocytosis is also required for the fibroblast growth factor (FGF)-mediated internalization of E-cadherin (Bryant et al., 2005) and vascular endothelial growth factor (VEGF)-mediated internalization of VE-cadherin (Gavard and Gutkind, 2006). A recent study using HUVECs has shown that pacsin 2 inhibits VE-cadherin internalization from trailing ends of FAJs without affecting the total surface levels of VE-cadherin (Dorland et al., 2016). However, our study clearly showed that pacsin 2 depletion inhibits N-cadherin internalization from the cell surface (Fig. 5). Since the cytoplasmic domains of VE- and N-cadherins are divergent in their amino acid sequences, pacsin 2 might associate with multiple cadherins in various ways to control their functions required in specific cell types.

Clathrin-independent endocytic pathways are also involved in the internalization of cadherins, though this process is poorly understood compared to clathrin-dependent endocytosis. Caveolin-dependent endocytosis of E-cadherin is required for disruption of cell–cell adhesion induced by EGF signalling, which is relevant to the EMT of cancer cells (Lu et al., 2003). Another study has shown that Rac1-modulated macropinocytosis is also required for the EGF-induced internalization of E-cadherin in breast carcinoma (Bryant et al., 2007). Clathrin- and caveolae-independent endocytosis is required for N-cadherin internalization to regulate early neuronal maturation *in vivo* (Shikanai et al., 2018). Pacsins and dynamins regulate clathrin-dependent endocytosis and caveolae-dependent endocytosis (Dessy et al., 2000; Henley et al., 1998; Qualmann and Kelly, 2000; Senju et al., 2011). Consistently, dynamin 2 RNAi cells phenocopied pacsin 2 RNAi cells in the formation of cell–cell contacts enriched with N-cadherin (Fig. S1), suggesting that pacsin 2 and dynamin 2 cooperatively regulate the internalization of N-cadherin in clathrin-dependent and/or -independent endocytic pathways.

Clathrin-mediated endocytosis of cadherins is regulated by p120, an armadillo family protein that binds to the cytoplasmic domain of classical cadherin (Reynolds, 2007). In p120-null SW48 colon carcinoma cells (Ireton et al., 2002) and p120-depleted microvascular endothelial cells (MECs; Xiao et al., 2003a), cadherins are degraded through an endo-lysosomal pathway, revealing that p120 plays essential roles in the regulation of cadherin endocytosis. Interestingly, the pacsin 2 SH3 domain bound to cytoplasmic regions of N-cadherin where two PxxP motifs are located in regions distinct from p120-binding sites (Fig. 6B). Indeed, an N-cadherin mutant with proline-to-alanine substitutions in the PxxP motifs failed to bind to pacsin 2 SH3 domain (Fig. 6D,E) and induced attenuation of N-cadherin internalization from the cell surface (Fig. 7). Thus, pacsin 2 might regulate N-cadherin endocytosis cooperatively with p120 and/or in a novel mechanism independent from p120-mediated regulation of endocytosis.

In this study, we also showed that depletion of either pacsin 2 or dynamin 2 increased the number of FAs in T24 cells (Fig. 8; Fig. S7). Increased FA numbers upon pacsin 2 RNAi and dynamin 2 RNAi were also observed even in single-cell conditions (Fig. S8), suggesting their direct roles in regulating FA turnover. Indeed, in NIH-3T3 cells, FA disassembly induced by microtubule regrowth after nocodazole washout depends on the recruitment of dynamin 2 to FAs (Ezratty et al., 2005). Another study has shown that the interaction between dynamin 2 and focal adhesion kinase (FAK, also known as PTK2) regulates FA dynamics in response to active Src (Wang et al., 2011). An additional study has shown that the clathrin-dependent pathway is the main pathway for dynamin 2-dependent endocytosis of FA components that leads to FA disassembly (Chao and Kunz, 2009). In contrast to dynamin 2, pacsin 2 function in FA turnover is largely unknown. Since pacsin 2 and dynamin 2 are cooperatively involved in clathrin-dependent and clathrin-independent endocytosis, further studies are required to reveal their precise function in FA turnover.

In collective cell migration, cell–cell and cell–ECM adhesions need to be finely balanced (Hamidi and Ivaska, 2018). Cell–cell adhesion molecules (e.g. cadherins) and FA molecules (e.g. integrins) share common signalling molecules, and they are physically linked intracellularly via the actin cytoskeleton (Mui et al., 2016). The convergence of crosstalk between these cell adhesion molecules is thought to be Rho-family GTPases (Combedazou et al., 2020). N-cadherin in non-tumour cells enhances collective cell migration via the polarization of Rho-family GTPases essential for cytoskeletal regulation (Mrozik et al., 2018). N-cadherin also facilitates collective cell migration by polarizing FAs in the leading cells by elevating Cdc42 and Rac1 activity towards the free leading edge, resulting in enhanced cell migration (Ouyang et al., 2013; Sabatini et al., 2008; Theveneau et al., 2010). Simultaneously, RhoA is also activated at the lateral and rear sides of the leading cells, inducing enhanced stress fibre formation and actomyosin contractility to establish robust cell–cell contacts (Carmona-Fontaine et al., 2008). The enhanced cell–cell contacts and FAs observed in either pacsin 2 RNAi (Figs 3, 8) or dynamin 2 RNAi cells (Figs S1, S7) suggest their key roles in distinct cell adhesion machinery required for coordinated movement of collectively migrating cells.

BAR domain proteins have been implicated in cancer metastasis by controlling cell motility, migration and invasion. CIP4 (Cdc42-interacting protein 4), an F-BAR domain protein, promotes formation of invadopodia in breast cancer cell lines (Kreider-Letterman et al., 2023; Pichot et al., 2010). Alternative studies using breast cancer cells have shown that CIP4 suppresses Src-induced invadopodia formation by promoting endocytosis of MT1-MMP (also known as MMP14) (Hu et al., 2011). An I-BAR domain protein, MIM (missing in metastasis, also known as MTSS1), suppresses metastasis by regulating cytoskeletal dynamics and lamellipodia formation, consequently affecting the invasion and metastatic behaviour of cancer cells (Woodings et al., 2003). Furthermore, an N-BAR domain protein, endophilin, regulates the endocytosis of EGFR by controlling F-actin cytoskeleton (Vehlow et al., 2013). Interestingly, expression of the brain-specific pacsin isoform pacsin 1 is negatively correlated with the malignancy of gliomas, indicating that pacsin 1 could play an essential role in the development of gliomas and be a potential new biomarker and targeted therapy site for gliomas (Zimu et al., 2021). Collectively migrating cancer cells have higher metastatic potential than singly migrating cells (Yang et al., 2019). Recent findings indicate that collective migration is characteristic of cancer metastasis of epithelial origin (Wang et al., 2016), including ovarian cancer (Choi et al., 2016), prostate cancer (Cui and Yamada, 2013), breast cancer (Aceto et al., 2014) and colorectal cancer (Chung et al., 2016). Based on TCGA PanCancer Atlas studies in cBioPortal, deep deletions or mutations in the pacsin 2 gene have been identified in samples from people with different types of malignant cancers, including ovarian, breast and bladder cancers (Cerami et al., 2012; Gao et al., 2013). Pacsin 2 has been implicated in various cellular functions such as cell migration (Meng et al., 2011), cell spreading (de Kreuk et al., 2011), EGF receptor internalization (de Kreuk et al., 2012) and collective cell migration (Dorland et al., 2016; Malinova et al., 2021) that are tightly associated with various malignancies. Thus, future studies of the correlation between pacsin 2 expression levels and the malignancy of various cancers might identify pacsin 2 as a potential therapeutic target in cancer metastasis.

## MATERIALS AND METHODS

### Molecular biology

Expression constructs were prepared using Gateway cloning (Thermo Fisher Scientific), as described previously (Fujise et al., 2021). To prepare entry clones for either full-length N-cadherin and the N-cadherin cytoplasmic domain or the pacsin 2 SH3 domain, PCR fragments amplified from clones of human N-cadherin (NM_001792) or pacsin 2 (NM_001184970) using primers described in Table S1 were used for B–P recombination with either pDONR201 (for full-length N-cadherin and pacsin 2 SH3) or pENTR/D-TOPO (for N-cadherin cytoplasmic domain). These entry clones were subcloned into pCI-based destination vectors for expressing GFP-tagged proteins in mammalian cells (N-cadherin cytoplasmic domain and full length) or pGEX6P2 destination vectors for expressing GST-tagged proteins in bacterial cells (pacsin 2 SH3) by L–R recombination.

### Cell culture, DNA transfection and RNAi

T24 (ATCC HTB-4), RT4 (ATCC HTB-2), H1299 (ATCC CRL-5803) and A549 (ATCC CCL-185) cells were cultured in RPMI-1640 medium (189-02025, FUJIFILM Wako Chemicals) supplemented with 10% fetal bovine serum (FBS) (26140-079, Gibco) and penicillin-streptomycin (PS) (100 unit/ml) (15140122, Thermo Fisher Scientific) at 37°C in humidified air with 5% CO_2_. HEK293T cells (ATCC CRL-3216) were grown in D-MEM (high glucose) with L-glutamine, Phenol Red, and sodium pyruvate (043-30085, FUJIFILM Wako chemicals) supplemented with 10% FBS (26140-079, Gibco) and PS (100 unit/ml) (15140122, Thermo Fisher Scientific) at 37°C in 5% CO_2_. HUVEC cells (C2519A, Lonza) were cultured on flasks coated with gelatin (G1393, Merck) in EBM-2 Endothelial Cell Basal Medium (CC3156, Lonza) supplemented with EGM-2 Endothelial Cell Growth Medium-2 SingleQuots (CC4176, Lonza) at 37°C in 5% CO_2_.

For transfection of T24 and HEK293T, Lipofectamine LTX with Plus Reagent (15338-100, Thermo Fisher Scientific) was used following the manufacturer's instructions. To transfect T24 cells for immunofluorescence microscopy, 70% confluent cells in VIOLAMO VTC-P24 24-well plates (2-8588-03, AS ONE) were transfected with 0.5 μg of expression plasmids, and cells were fixed 48 h after the transfection. For surface biotinylation and endocytosis assays using T24 cells and GST pull-down assays using HEK293T cells, 70% confluent cells in 100 mm TC-treated culture dishes (430167, Corning) were transfected with 15 μg expression plasmids, and cells were collected 24 h (T24 cells) or 48 h (HEK293T cells) after the transfection to use for further analyses.

For RNAi of T24 cells and H1299 cells, 70% confluent cells in 24-well plates were transfected with 10 pmol of either siGENOME SMART pool siRNA for human Dnm2 (M-04007-03, Dharmacon) or siGENOME nontargeting siRNA Pool #1 (D-001206-13-05, Dharmacon), Mission siRNA for human pacsin 2 siRNA [SASI_Hs01_0021-5539 (siPacsin2#1), SASI_Hs01_0021-5540 (siPacsin2#2), SASI_Hs01_0021-5538 (siPacsin2#3); Merck] or MISSION siRNA Universal Negative Control #1 (SIC-001, Merck) using Lipofectamine RNAiMAX Transfection Reagent (13778150, Thermo Fisher Scientific) following manufacturer's instructions. siPacsin2#2 (SASI_Hs01_0021-5540) was used for figures that just mention siPacsin2 (e.g. Fig. 2D-G, Fig. 4, Fig. 5; Movie 2).

### Antibodies and reagents

Primary antibodies used in this study were rabbit polyclonal anti-dynamin 2 (ab65556, Abcam), rabbit polyclonal anti-PACSIN1 (M-46; sc-30127, Santa Cruz Biotechnology), mouse monoclonal anti-PACSIN 2 (SAB1402538-100UG, SIGMA), mouse monoclonal anti-PACSIN3 (C-3; sc-166923, Santa Cruz Biotechnology), mouse monoclonal anti-E-cadherin (610181, BD Transduction laboratory), mouse monoclonal anti-N-cadherin (610920, BD Transduction laboratory), mouse monoclonal anti-P-cadherin (12H6, Cell Signaling Technology), mouse monoclonal anti-VE-cadherin (610251, BD Transduction laboratory), mouse monoclonal anti-paxillin (5H11) (AH00492, Thermo Fisher Scientific), mouse monoclonal anti-α tubulin (T5168, Merck), rabbit polyclonal anti-cortactin (A302-608A-M, Thermo Fisher Scientific), anti-GFP (D5.1) XP rabbit mAb (2956, Cell Signaling Technology) and mouse monoclonal α-catenin (G-11) (sc-9988, Santa Cruz Biotechnology). Secondary antibodies and Alexa Fluor-conjugated phalloidin for immunofluorescence microscopy were purchased from Thermo Fisher Scientific: Alexa Fluor 488 donkey anti-goat IgG (H+L) (A-11055), Alexa Fluor 488 donkey anti-rabbit IgG (H+L) (A-21206), Alexa Fluor 555 donkey anti-mouse IgG (H+L) (A-31570), Alexa Fluor 555 donkey anti-rabbit IgG (H+L) (A-31572) and Alexa Fluor 555 Phalloidin (A-34055). Secondary antibodies for immunoblot analyses were also purchased from Thermo Fisher Scientific: goat anti-rabbit IgG (H+L) secondary antibody, HRP (31460); and rabbit anti-mouse IgG (H+L) secondary antibody, HRP (31450).

Paraformaldehyde used for cell fixation was prepared from 16% paraformaldehyde (15710, Electron Microscopy Sciences). Glutaraldehyde used for fixing gelatin-coated coverslips was prepared from glutaraldehyde 25% EM (G004, TAAB).

### Immunofluorescence microscopy

Immunofluorescence microscopy of T24 and H1299 cells on gelatin-coated coverslips was performed as described previously (Li et al., 2021) using primary antibodies (1:100 dilution for anti-N-cadherin; 1:200 dilution for anti-dynamin 2, anti-cortactin, anti-paxillin and anti-pacsin 2; 1:500 dilution for anti-pacsin 1 and anti-pacsin 3; and 1:400 for anti-α-catenin) and secondary antibodies (1:1000 dilution), as well as Alexa Fluor 555 Phalloidin (1:1000 dilution). DNA was stained using 1:10,000 dilution of Hoechst 33258 (343-07961, DOJINDO). FITC–gelatin was also prepared as described previously (Li et al., 2021). Immunostained samples for T24 cells were visualized using a BX51 fluorescence microscope (OLYMPUS) with a 40× NA 0.75 objective lens, and images were acquired with Discovery MH15 CMOS camera (Tucsen) and ISCapture image acquisition software (Tucsen). All images were analysed using Fiji (Schindelin et al., 2012) and processed with Adobe Photoshop 2022 (Adobe).

### Immunoblot analysis

Immunoblot analyses were performed as described previously (Fujise et al., 2021) using primary antibodies (1:1000 dilution) and secondary antibodies (1:10,000 dilution). For signal detection, either Amersham ECL Prime (RPN2232, Cytiva) or SuperSignal West Pico PLUS Chemiluminescent Substrate (34580, Thermo Fisher Scientific) was used. For Coomassie Brilliant Blue (CBB) staining, Brilliant Blue R (27816, Merck) was used at 0.225% (w/v) in 50% (v/v) methanol and 10% (v/v) acetic acid.

### Wound healing assay

Confluent T24 cells transfected with siRNAs for control and pacsin 2 RNAi in 6-well plates were treated with 10 ng/ml of recombinant human EGF (236-EG, R&D systems) in RPMI-1640 medium and incubated at 37°C, 5% CO_2_ for 2 h. After 2 h, the cell monolayer was scratched with a sterile 200 μl pipette tip. Recovery processes were captured after 0, 6 and 12 h using a NEX-5N camera (Sony) attached to a Nikon Eclipse Ts2 microscope using a 2× objective lens.

For live-cell imaging of the wound healing assay, T24 cells (0.6×10^6^ cells) transfected with either control siRNA or pacsin 2 siRNA were seeded in a 35 mm glass-base dish (3910-035, IWAKI) and incubated at 37°C, 5% CO_2_ until the cell monolayer became confluent. Cells were then treated with 10 ng/ml of recombinant human EGF (236-EG, R&D systems) in RPMI-1640 medium and incubated at 37°C, 5% CO_2_ for 2 h. After the cell monolayer was scratched with a sterile 20 µl pipette tip, cells were maintained in 5% CO_2_ at 37°C with a thermo-control system (MI-IBC, OLYMPUS), and images were acquired on an IX71 microscope (OLYMPUS) fitted with an X-Light spinning disc confocal unit (CrestOptics) and iXon EMCCD camera (DU-888E-C00-#BV, ANDOR) using MetaMorph (Molecular Devices). Images were captured with a 20× objective lens (LCPlanFI 20×/0.40) at 1-min intervals for 6 h.

For cell tracking analyses, ten cells at the front of either side of the wound edge were randomly selected and tracked using the Manual Tracking plugins of Fiji (Schindelin et al., 2012). Cell velocity and directionality were determined by analysing the trajectories of 10 cells/movie from four independent movies (40 cells each for control and pacsin 2 RNAi) using the Chemotaxis Tool (ibidi). Cell traces were analysed using OriginPro 2021 (Origin Lab).

### GST pull-down assays

The recombinant protein of human pacsin 2 SH3 domain (amino acids 426–486) used as bait for the GST pull-down assay was expressed and purified with the GST Gene Fusion System (Cytiva) as a GST fusion using Glutathione Sepharose 4B (17075601, Cytiva) according to the manufacturer's instructions. The GFP-tagged cytoplasmic domain of N-cadherin (amino acids 746–906) was expressed in HEK293T cells, and the cell extract was prepared by sonication using a TAITEC VP-5S sonicator (output: 5; 3×5 s) in extraction buffer [20 mM Tris-HCl, pH 7.5, 150 mM NaCl, 10% glycerol, 5 mM EDTA, 2% Triton X-100, 1 mM PMSF and protease inhibitor (11697498001, Roche)] followed by centrifugation at 20,600 ***g*** for 10 min at 4°C. For the pull-down assay, 170 µl of the cell extract was added to 10 µl (bed volume) of GST–pacsin 2 SH3 or GST beads in the extraction buffer and mixed for 1 h at 4°C with gentle agitation. After washing three times with the extraction buffer, the bound proteins were analysed by immunoblot analyses.

### Surface biotinylation and endocytosis assay

The surface biotinylation and endocytosis assay was performed as described previously (Morimoto et al., 2005) with minor modifications. T24 cells (2.5×10^5^ cells) transfected with control siRNA or pacsin 2 siRNA for 48 h were seeded in a 100 mm TC-treated culture dish (430167, Corning) in RPMI-1640 medium. Alternatively, T24 cells (3.2×10^6^ cells) in a 100 mm TC-treated culture dish were transfected with constructs encoding either GFP-tagged full-length wild-type N-cadherin (NCADWt–GFP) or GFP-tagged full-length PA mutant N-cadherin (NCADPA–GFP). After 24 h, cell surface proteins were biotinylated with 0.5 mg/ml Ez-link sulfo-NHS-SS-Biotin (21331, Thermo Fisher Scientific) in PBS containing 1 mM CaCl_2_ and 0.5 mM MgCl_2_ (PBS/CM) at 4°C for 30 min, and quenched with 50 mM NH_4_Cl in PBS/CM at 4°C for 15 min. The cells were then allowed to endocytose at 37°C for the indicated periods until the endocytosis was stopped by rapid cooling of the cells on ice at 4°C. The remaining biotin on the cell surface was stripped with 50 mM sodium 2-mercaptoethanesulfonate (MESNa) (M1511, Sigma-Aldrich) in PBS/CM at 4°C for 30 min followed by quenching with 5 mg/ml iodoacetamide (093-02152, Fujifilm) in PBS/CM at 4°C for 15 min. Cells were then lysed with RIPA buffer [20 mM Tris-HCl pH 7.6, 150 mM NaCl, 0.1% SDS, 1% Triton X-100, 1% deoxycholate, 5 mM EDTA with protease inhibitors (11697498001, Roche)] by sonication using a TAITEC VP-5S sonicator (output: 5; 2×5 s), and cell lysate was recovered in the supernatant after centrifugation at 20,000 ***g*** at 4°C for 15 min. The cell lysates containing an equal amount of total proteins (≃2 mg in 1 ml RIPA buffer) were incubated with 15 µl (bed volume) of Pierce NeutrAvidin agarose (29200, Thermo Fisher Scientific) for 2 h at 4°C to capture internalized biotinylated protein. After washing three times in RIPA buffer, biotinylated proteins were eluted from NeutrAvidin agarose beads by sample buffer, separated by SDS–PAGE and analysed by immunoblotting using an antibody against N-cadherin. The relative amount of internalized N-cadherin (pull-down) was obtained by normalizing it to the total amount of N-cadherin expressed in the cells.

### Electron microscopy

T24 cells were fixed in 2% glutaraldehyde and 2% paraformaldehyde at 4°C overnight and then post fixed in 2% osmium tetraoxide for 1.5 h at 4°C followed by dehydration with ethanol and embedding in Spurr resin (Polysciences Inc., Warrington, PA, USA). Ultrathin sections of the samples were prepared using an ultramicrotome (Leica EM UC7), and they were stained with uranyl acetate and lead and observed by electron microscope H-7650 (Hitachi, Tokyo, Japan) at Central Research Laboratory (Okayama University Medical School).

### Image analyses of N-cadherin and FAs

To quantitatively evaluate the localization of GFP-tagged N-cadherin or α-catenin, 25×25-pixel regions of interest (ROIs) were manually located on cell–cell contact sites, and then maximum intensities were measured from the 2-pixel-Gaussian filtered fluorescently labelled N-cadherin images using ImageJ software (Schneider et al., 2012). Intensity ratios of GFP-tagged N-cadherin to α-catenin were also calculated.

To quantitatively evaluate the intracellular distribution of FAs, automatic image processing was performed using ImageJ. First, the cell regions were determined by the Triangle intensity thresholding of 5–35-pixel-band-pass filtered images of fluorescently labelled actin filaments. Next, using the ‘Distance Map’ function in ImageJ, the relative position from the cell contour was quantified. The position of the cell contour was given a minimum value of 0 and the position farthest from the cell contour was given a maximum value of 1. Then, the regions of FAs in the cell regions were automatically detected by the Yen's intensity thresholding of 1–4-pixel-band-pass filtered images of fluorescently labelled paxillin. Finally, the average distance from the cell periphery of FA regions with more than 30 pixels was measured.

### Statistical analysis

All the experiments were repeated three times independently. Data were analysed for statistical significance using GraphPad Prism6 (GraphPad Software).

## Supplementary Material

10.1242/joces.260827_sup1 Supplementary information

## References

[JCS260827C1] Aceto, N., Bardia, A., Miyamoto, D. T., Donaldson, M. C., Wittner, B. S., Spencer, J. A., Yu, M., Pely, A., Engstrom, A., Zhu, H. et al. (2014). Circulating tumor cell clusters are oligoclonal precursors of breast cancer metastasis. *Cell* 158, 1110-1122. 10.1016/j.cell.2014.07.013 25171411 PMC4149753

[JCS260827C2] Akhtar, N. and Hotchin, N. A. (2001). Rac1 regulates adherens junctions through endocytosis of E-cadherin. *Mol. Biol. Cell* 12, 847-862. 10.1091/mbc.12.4.847 11294891 PMC32271

[JCS260827C3] Bryant, D. M., Wylie, F. G. and Stow, J. L. (2005). Regulation of endocytosis, nuclear translocation, and signaling of fibroblast growth factor receptor 1 by E-cadherin. *Mol. Biol. Cell* 16, 14-23. 10.1091/mbc.e04-09-0845 15509650 PMC539147

[JCS260827C4] Bryant, D. M., Kerr, M. C., Hammond, L. A., Joseph, S. R., Mostov, K. E., Teasdale, R. D. and Stow, J. L. (2007). EGF induces macropinocytosis and SNX1-modulated recycling of E-cadherin. *J. Cell Sci.* 120, 1818-1828. 10.1242/jcs.000653 17502486

[JCS260827C5] Cadwell, C. M., Su, W. and Kowalczyk, A. P. (2016). Cadherin tales: regulation of cadherin function by endocytic membrane trafficking. *Traffic* 17, 1262-1271. 10.1111/tra.12448 27624909

[JCS260827C6] Carmona-Fontaine, C., Matthews, H. K., Kuriyama, S., Moreno, M., Dunn, G. A., Parsons, M., Stern, C. D. and Mayor, R. (2008). Contact inhibition of locomotion in vivo controls neural crest directional migration. *Nature* 456, 957-961. 10.1038/nature07441 19078960 PMC2635562

[JCS260827C7] Cerami, E., Gao, J., Dogrusoz, U., Gross, B. E., Sumer, S. O., Aksoy, B. A., Jacobsen, A., Byrne, C. J., Heuer, M. L., Larsson, E. et al. (2012). The cBio cancer genomics portal: an open platform for exploring multidimensional cancer genomics data. *Cancer Discov.* 2, 401-404. 10.1158/2159-8290.CD-12-0095 22588877 PMC3956037

[JCS260827C8] Chandrasekaran, R., Kenworthy, A. K. and Lacy, D. B. (2016). Clostridium difficile Toxin a undergoes clathrin-independent, PACSIN2-dependent endocytosis. *PLoS Pathog.* 12, 1-30. 10.1371/journal.ppat.1006070 PMC5152916 27942025

[JCS260827C9] Chao, W.-T. and Kunz, J. (2009). Focal adhesion disassembly requires clathrin-dependent endocytosis of integrins. *FEBS Lett.* 583, 1337-1343. 10.1016/j.febslet.2009.03.037 19306879 PMC2801759

[JCS260827C10] Chen, Y.-T. and Tai, C.-Y. (2017). μ2-Dependent endocytosis of N-cadherin is regulated by β-catenin to facilitate neurite outgrowth. *Traffic* 18, 287-303. 10.1111/tra.12473 28224728

[JCS260827C11] Chiasson, C. M., Wittich, K. B., Vincent, P. A., Faundez, V. and Kowalczyk, A. P. (2009). p120-catenin inhibits VE-cadherin internalization through a Rho-independent mechanism. *Mol. Biol. Cell* 20, 1970-1980. 10.1091/mbc.e08-07-0735 19211843 PMC2663933

[JCS260827C12] Choi, P.-W., Yang, J., Ng, S.-K., Feltmate, C., Muto, M. G., Hasselblatt, K., Lafferty-Whyte, K., JeBailey, L., MacConaill, L., Welch, W. R. et al. (2016). Loss of E-cadherin disrupts ovarian epithelial inclusion cyst formation and collective cell movement in ovarian cancer cells. *Oncotarget* 7, 4110-4121. 10.18632/oncotarget.6588 26684027 PMC4826193

[JCS260827C13] Chung, Y.-C., Wei, W.-C., Hung, C.-N., Kuo, J.-F., Hsu, C.-P., Chang, K.-J. and Chao, W.-T. (2016). Rab11 collaborates E-cadherin to promote collective cell migration and indicates a poor prognosis in colorectal carcinoma. *Eur. J. Clin. Invest.* 46, 1002-1011. 10.1111/eci.12683 27696383

[JCS260827C14] Combedazou, A., Gayral, S., Colombié, N., Fougerat, A., Laffargue, M. and Ramel, D. (2020). Small GTPases orchestrate cell-cell communication during collective cell movement. *Small GTPases* 11, 103-112. 10.1080/21541248.2017.1366965 28980871 PMC7053965

[JCS260827C15] Cui, Y. and Yamada, S. (2013). N-cadherin dependent collective cell invasion of prostate cancer cells is regulated by the N-terminus of alpha-catenin. *PLoS ONE* 8, e55069. 10.1371/journal.pone.0055069 23359820 PMC3554680

[JCS260827C16] Davis, M. A., Ireton, R. C. and Reynolds, A. B. (2003). A core function for p120-catenin in cadherin turnover. *J. Cell Biol.* 163, 525-534. 10.1083/jcb.200307111 14610055 PMC2173649

[JCS260827C17] de Kreuk, B.-J., Nethe, M., Fernandez-Borja, M., Anthony, E. C., Hensbergen, P. J., Deelder, A. M., Plomann, M. and Hordijk, P. L. (2011). The F-BAR domain protein PACSIN2 associates with Rac1 and regulates cell spreading and migration. *J. Cell Sci.* 124, 2375-2388. 10.1242/jcs.080630 21693584

[JCS260827C18] de Kreuk, B.-J., Anthony, E. C., Geerts, D. and Hordijk, P. L. (2012). The F-BAR protein PACSIN2 regulates epidermal growth factor receptor internalization. *J. Biol. Chem.* 287, 43438-43453. 10.1074/jbc.M112.391078 23129763 PMC3527931

[JCS260827C19] Dessy, C., Kelly, R. A., Balligand, J. L. and Feron, O. (2000). Dynamin mediates caveolar sequestration of muscarinic cholinergic receptors and alteration in NO signaling. *EMBO J.* 19, 4272-4280. 10.1093/emboj/19.16.4272 10944110 PMC302031

[JCS260827C20] Dorland, Y. L., Malinova, T. S., van Stalborch, A.-M. D., Grieve, A. G., van Geemen, D., Jansen, N. S., de Kreuk, B.-J., Nawaz, K., Kole, J., Geerts, D. et al. (2016). The F-BAR protein pacsin2 inhibits asymmetric VE-cadherin internalization from tensile adherens junctions. *Nat. Commun.* 7, 12210. 10.1038/ncomms12210 27417273 PMC4947187

[JCS260827C21] Dumont, V. and Lehtonen, S. (2022). PACSIN proteins in vivo: roles in development and physiology. *Acta Physiol. (Oxf.)* 234, e13783. 10.1111/apha.13783 34990060 PMC9285741

[JCS260827C22] Elie-Caille, C., Lascombe, I., Péchery, A., Bittard, H. and Fauconnet, S. (2020). Molecular and nanoscale evaluation of N-cadherin expression in invasive bladder cancer cells under control conditions or GW501516 exposure. *Mol. Cell. Biochem.* 471, 113-127. 10.1007/s11010-020-03771-1 32519230 PMC7370938

[JCS260827C23] Ezratty, E. J., Partridge, M. A. and Gundersen, G. G. (2005). Microtubule-induced focal adhesion disassembly is mediated by dynamin and focal adhesion kinase. *Nat. Cell Biol.* 7, 581-590. 10.1038/ncb1262 15895076

[JCS260827C24] Friedl, P. and Gilmour, D. (2009). Collective cell migration in morphogenesis, regeneration and cancer. *Nat. Rev. Mol. Cell Biol.* 10, 445-457. 10.1038/nrm2720 19546857

[JCS260827C25] Fujise, K., Okubo, M., Abe, T., Yamada, H., Nishino, I., Noguchi, S., Takei, K. and Takeda, T. (2021). Mutant BIN1-Dynamin 2 complexes dysregulate membrane remodeling in the pathogenesis of centronuclear myopathy. *J. Biol. Chem.* 296, 100077. 10.1074/jbc.RA120.015184 33187981 PMC7949082

[JCS260827C26] Gao, J., Aksoy, B. A., Dogrusoz, U., Dresdner, G., Gross, B., Sumer, S. O., Sun, Y., Jacobsen, A., Sinha, R., Larsson, E. et al. (2013). Integrative analysis of complex cancer genomics and clinical profiles using the cBioPortal. *Sci. Signal.* 6, pl1. 10.1126/scisignal.2004088 23550210 PMC4160307

[JCS260827C27] Gavard, J. and Gutkind, J. S. (2006). VEGF controls endothelial-cell permeability by promoting the β-arrestin-dependent endocytosis of VE-cadherin. *Nat. Cell Biol.* 8, 1223-1234. 10.1038/ncb1486 17060906

[JCS260827C28] Gumbiner, B. M. (2005). Regulation of cadherin-mediated adhesion in morphogenesis. *Nat. Rev. Mol. Cell Biol.* 6, 622-634. 10.1038/nrm1699 16025097

[JCS260827C29] Haeger, A., Wolf, K., Zegers, M. M. and Friedl, P. (2015). Collective cell migration: Guidance principles and hierarchies. *Trends Cell Biol.* 25, 556-566. 10.1016/j.tcb.2015.06.003 26137890

[JCS260827C30] Halbleib, J. M. and Nelson, W. J. (2006). Cadherins in development: cell adhesion, sorting, and tissue morphogenesis. *Genes Dev.* 20, 3199-3214. 10.1101/gad.1486806 17158740

[JCS260827C31] Hamidi, H. and Ivaska, J. (2018). Every step of the way: integrins in cancer progression and metastasis. *Nat. Rev. Cancer* 18, 533-548. 10.1038/s41568-018-0038-z 30002479 PMC6629548

[JCS260827C32] Hansen, C. G., Howard, G. and Nichols, B. J. (2011). Pacsin 2 is recruited to caveolae and functions in caveolar biogenesis. *J. Cell Sci.* 124, 2777-2785. 10.1242/jcs.084319 21807942

[JCS260827C33] Hayer, A., Shao, L., Chung, M., Joubert, L.-M., Yang, H. W., Tsai, F.-C., Bisaria, A., Betzig, E. and Meyer, T. (2016). Engulfed cadherin fingers are polarized junctional structures between collectively migrating endothelial cells. *Nat. Cell Biol.* 18, 1311-1323. 10.1038/ncb3438 27842057 PMC6159904

[JCS260827C34] Henley, J. R., Krueger, E. W. A., Oswald, B. J. and McNiven, M. A. (1998). Dynamin-mediated internalization of caveolae. *J. Cell Biol.* 141, 85-99. 10.1083/jcb.141.1.85 9531550 PMC2132718

[JCS260827C35] Hu, J., Mukhopadhyay, A., Truesdell, P., Chander, H., Mukhopadhyay, U. K., Mak, A. S. and Craig, A. W. B. (2011). Cdc42-interacting protein 4 is a Src substrate that regulates invadopodia and invasiveness of breast tumors by promoting MT1-MMP endocytosis. *J. Cell Sci.* 124, 1739-1751. 10.1242/jcs.078014 21525036

[JCS260827C36] Ireton, R. C., Davis, M. A., van Hengel, J., Mariner, D. J., Barnes, K., Thoreson, M. A., Anastasiadis, P. Z., Matrisian, L., Bundy, L. M., Sealy, L. et al. (2002). A novel role for p120 catenin in E-cadherin function. *J. Cell Biol.* 159, 465-476. 10.1083/jcb.200205115 12427869 PMC2173073

[JCS260827C37] Kaszak, I., Witkowska-Pilaszewicz, O., Niewiadomska, Z., Dworecka-Kaszak, B., Ngosa Toka, F. and Jurka, P. (2020). Role of cadherins in cancer-a review. *Int. J. Mol. Sci.* 21, 7624. 10.3390/ijms21207624 33076339 PMC7589192

[JCS260827C38] Klymenko, Y., Kim, O., Loughran, E., Yang, J., Lombard, R., Alber, M. and Stack, M. S. (2017). Cadherin composition and multicellular aggregate invasion in organotypic models of epithelial ovarian cancer intraperitoneal metastasis. *Oncogene* 36, 5840-5851. 10.1038/onc.2017.171 28628116 PMC5648607

[JCS260827C39] Kowalczyk, A. P. and Nanes, B. A. (2012). Adherens junction turnover: regulating adhesion through cadherin endocytosis, degradation, and recycling. *Subcell. Biochem.* 60, 197-222. 10.1007/978-94-007-4186-7_9 22674073 PMC4074012

[JCS260827C40] Kreider-Letterman, G., Castillo, A., Mahlandt, E. K., Goedhart, J., Rabino, A., Goicoechea, S. and Garcia-Mata, R. (2023). ARHGAP17 regulates the spatiotemporal activity of Cdc42 at invadopodia. *J. Cell Biol.* 222, e202207020. 10.1083/jcb.202207020 36571786 PMC9794838

[JCS260827C41] Kuriyama, S., Yoshida, M., Yano, S., Aiba, N., Kohno, T., Minamiya, Y., Goto, A. and Tanaka, M. (2016). LPP inhibits collective cell migration during lung cancer dissemination. *Oncogene* 35, 952-964. 10.1038/onc.2015.155 26028032

[JCS260827C42] Le, T. L., Yap, A. S. and Stow, J. L. (1999). Recycling of E-cadherin: A potential mechanism for regulating cadherin dynamics. *J. Cell Biol.* 146, 219-232. 10.1083/jcb.146.999.219 10402472 PMC2199726

[JCS260827C43] Li, J., Fujise, K., Wint, H., Senju, Y., Suetsugu, S., Yamada, H., Takei, K. and Takeda, T. (2021). Dynamin 2 and BAR domain protein pacsin 2 cooperatively regulate formation and maturation of podosomes. *Biochem. Biophys. Res. Commun.* 571, 145-151. 10.1016/j.bbrc.2021.07.041 34325130

[JCS260827C44] Lu, Z., Ghosh, S., Wang, Z. and Hunter, T. (2003). Downregulation of caveolin-1 function by EGF leads to the loss of E-cadherin, increased transcriptional activity of β-catenin, and enhanced tumor cell invasion. *Cancer Cell* 4, 499-515. 10.1016/S1535-6108(03)00304-0 14706341

[JCS260827C45] Maeda, M., Johnson, K. R. and Wheelock, M. J. (2005). Cadherin switching: essential for behavioral but not morphological changes during an epithelium-to-mesenchyme transition. *J. Cell Sci.* 118, 873-887. 10.1242/jcs.01634 15713751

[JCS260827C46] Malinova, T. S., Angulo-Urarte, A., Nüchel, J., Tauber, M., van der Stoel, M. M., Janssen, V., de Haan, A., Groenen, A. G., Tebbens, M., Graupera, M. et al. (2021). A junctional PACSIN2/EHD4/MICAL-L1 complex coordinates VE-cadherin trafficking for endothelial migration and angiogenesis. *Nat. Commun.* 12, 2610. 10.1038/s41467-021-22873-y 33972531 PMC8110786

[JCS260827C47] Meng, H., Tian, L., Zhou, J., Li, Z., Jiao, X., Li, W. W., Plomann, M., Xu, Z., Lisanti, M. P., Wang, C. et al. (2011). PACSIN 2 represses cellular migration through direct association with cyclin D1 but not its alternate splice form cyclin D1b. *Cell Cycle* 10, 73-81. 10.4161/cc.10.1.14243 21200149 PMC3048077

[JCS260827C48] Mishra, D. K., Miller, R. A., Pence, K. A. and Kim, M. P. (2018). Small cell and non small cell lung cancer form metastasis on cellular 4D lung model. *BMC Cancer* 18, 441. 10.1186/s12885-018-4358-x 29669530 PMC5907356

[JCS260827C49] Miyashita, Y. and Ozawa, M. (2007). Increased internalization of p120-uncoupled E-cadherin and a requirement for a dileucine motif in the cytoplasmic domain for endocytosis of the protein. *J. Biol. Chem.* 282, 11540-11548. 10.1074/jbc.M608351200 17298950

[JCS260827C50] Modregger, J., Ritter, B., Witter, B., Paulsson, M. and Plomann, M. (2000). All three PACSIN isoforms bind to endocytic proteins and inhibit endocytosis. *J. Cell Sci.* 113, 4511-4521. 10.1242/jcs.113.24.4511 11082044

[JCS260827C51] Morimoto, S., Nishimura, N., Terai, T., Manabe, S., Yamamoto, Y., Shinahara, W., Miyake, H., Tashiro, S., Shimada, M. and Sasaki, T. (2005). Rab13 mediates the continuous endocytic recycling of occludin to the cell surface. *J. Biol. Chem.* 280, 2220-2228. 10.1074/jbc.M406906200 15528189

[JCS260827C52] Mrozik, K. M., Blaschuk, O. W., Cheong, C. M., Zannettino, A. C. W. and Vandyke, K. (2018). N-cadherin in cancer metastasis, its emerging role in haematological malignancies and potential as a therapeutic target in cancer. *BMC Cancer* 18, 939. 10.1186/s12885-018-4845-0 30285678 PMC6167798

[JCS260827C53] Mui, K. L., Chen, C. S. and Assoian, R. K. (2016). The mechanical regulation of integrin-cadherin crosstalk organizes cells, signaling and forces. *J. Cell Sci.* 129, 1093-1100. 10.1242/jcs.183699 26919980 PMC4813297

[JCS260827C54] Nieto, M. A., Huang, R. Y.-J., Jackson, R. A. and Thiery, J. P. (2016). Emt: 2016. *Cell* 166, 21-45. 10.1016/j.cell.2016.06.028 27368099

[JCS260827C55] Oda, H. and Takeichi, M. (2011). Evolution: structural and functional diversity of cadherin at the adherens junction. *J. Cell Biol.* 193, 1137-1146. 10.1083/jcb.201008173 21708975 PMC3216324

[JCS260827C56] Ouyang, M., Lu, S., Kim, T., Chen, C.-E., Seong, J., Leckband, D. E., Wang, F., Reynolds, A. B., Schwartz, M. A. and Wang, Y. (2013). N-cadherin regulates spatially polarized signals through distinct p120ctn and β-catenin-dependent signalling pathways. *Nat. Commun.* 4, 1589. 10.1038/ncomms2560 23481397 PMC3602931

[JCS260827C57] Pandya, P., Orgaz, J. L. and Sanz-Moreno, V. (2017). Modes of invasion during tumour dissemination. *Mol. Oncol.* 11, 5-27. 10.1002/1878-0261.12019 28085224 PMC5423224

[JCS260827C58] Paterson, A. D., Parton, R. G., Ferguson, C., Stow, J. L. and Yap, A. S. (2003). Characterization of E-cadherin endocytosis in isolated MCF-7 and Chinese hamster ovary cells. The initial fate of unbound E-cadherin. *J. Biol. Chem.* 278, 21050-21057. 10.1074/jbc.M300082200 12657640

[JCS260827C59] Peter, B. J., Kent, H. M., Mills, I. G., Vallis, Y., Butler, P. J. G., Evans, P. R. and McMahon, H. T. (2004). BAR domains as sensors of membrane curvature: the amphiphysin BAR structure. *Science* 303, 495-499. 10.1126/science.1092586 14645856

[JCS260827C60] Pichot, C. S., Arvanitis, C., Hartig, S. M., Jensen, S. A., Bechill, J., Marzouk, S., Yu, J., Frost, J. A. and Corey, S. J. (2010). Cdc42-interacting protein 4 promotes breast cancer cell invasion and formation of invadopodia through activation of N-WASp. *Cancer Res.* 70, 8347-8356. 10.1158/0008-5472.CAN-09-4149 20940394 PMC2970640

[JCS260827C61] Pinheiro, P. S., Jansen, A. M., de Wit, H., Tawfik, B., Madsen, K. L., Verhage, M., Gether, U. and Sorensen, J. B. (2014). The BAR domain protein PICK1 controls vesicle number and size in adrenal chromaffin cells. *J. Neurosci.* 34, 10688-10700. 10.1523/JNEUROSCI.5132-13.2014 25100601 PMC4122802

[JCS260827C62] Plutoni, C., Bazellieres, E., Le Borgne-Rochet, M., Comunale, F., Brugues, A., Séveno, M., Planchon, D., Thuault, S., Morin, N., Bodin, S. et al. (2016). P-cadherin promotes collective cell migration via a Cdc42-mediated increase in mechanical forces. *J. Cell Biol.* 212, 199-217. 10.1083/jcb.201505105 26783302 PMC4738379

[JCS260827C63] Qualmann, B. and Kelly, R. B. (2000). Syndapin isoforms participate in receptor-mediated endocytosis and actin organization. *J. Cell Biol.* 148, 1047-1062. 10.1083/jcb.148.5.1047 10704453 PMC2174535

[JCS260827C64] Qualmann, B., Koch, D. and Kessels, M. M. (2011). Let's go bananas: revisiting the endocytic BAR code. *EMBO J.* 30, 3501-3515. 10.1038/emboj.2011.266 21878992 PMC3181480

[JCS260827C65] Ratheesh, A. and Yap, A. S. (2012). A bigger picture: classical cadherins and the dynamic actin cytoskeleton. *Nat. Rev. Mol. Cell Biol.* 13, 673-679. 10.1038/nrm3431 22931853

[JCS260827C66] Ray, A., Lee, O., Win, Z., Edwards, R. M., Alford, P. W., Kim, D.-H. and Provenzano, P. P. (2017). Anisotropic forces from spatially constrained focal adhesions mediate contact guidance directed cell migration. *Nat. Commun.* 8, 14923. 10.1038/ncomms14923 28401884 PMC5394287

[JCS260827C67] Reynolds, A. B. (2007). p120-catenin: past and present. *Biochim. Biophys. Acta* 1773, 2-7. 10.1016/j.bbamcr.2006.09.019 17175391 PMC2892545

[JCS260827C68] Rørth, P. (2009). Collective cell migration. *Annu. Rev. Cell Dev. Biol.* 25, 407-429. 10.1146/annurev.cellbio.042308.113231 19575657

[JCS260827C69] Sabatini, P. J. B., Zhang, M., Silverman-Gavrila, R., Bendeck, M. P. and Langille, B. L. (2008). Homotypic and endothelial cell adhesions via N-cadherin determine polarity and regulate migration of vascular smooth muscle cells. *Circ. Res.* 103, 405-412. 10.1161/CIRCRESAHA.108.175307 18617695

[JCS260827C70] Safari, F. and Suetsugu, S. (2012). The BAR domain superfamily proteins from subcellular structures to human diseases. *Membranes (Basel)* 2, 91-117. 10.3390/membranes2010091 24957964 PMC4021885

[JCS260827C71] Sánchez-Barrena, M. J., Vallis, Y., Clatworthy, M. R., Doherty, G. J., Veprintsev, D. B., Evans, P. R. and McMahon, H. T. (2012). Bin2 is a membrane sculpting N-BAR protein that influences leucocyte podosomes, motility and phagocytosis. *PLoS ONE* 7, e52401. 10.1371/journal.pone.0052401 23285027 PMC3527510

[JCS260827C72] Schindelin, J., Arganda-Carreras, I., Frise, E., Kaynig, V., Longair, M., Pietzsch, T., Preibisch, S., Rueden, C., Saalfeld, S., Schmid, B. et al. (2012). Fiji: an open-source platform for biological-image analysis. *Nat. Methods* 9, 676-682. 10.1038/nmeth.2019 22743772 PMC3855844

[JCS260827C73] Schneider, C. A., Rasband, W. S. and Eliceiri, K. W. (2012). NIH Image to ImageJ: 25 years of image analysis. *Nat. Methods* 9, 671-675. 10.1038/nmeth.2089 22930834 PMC5554542

[JCS260827C74] Senju, Y., Itoh, Y., Takano, K., Hamada, S. and Suetsugu, S. (2011). Essential role of PACSIN2/syndapin-II in caveolae membrane sculpting. *J. Cell Sci.* 124, 2032-2040. 10.1242/jcs.086264 21610094

[JCS260827C75] Shellard, A. and Mayor, R. (2021). Collective durotaxis along a self-generated stiffness gradient in vivo. *Nature* 600, 690-694. 10.1038/s41586-021-04210-x 34880503

[JCS260827C76] Shih, W. and Yamada, S. (2012). N-cadherin-mediated cell-cell adhesion promotes cell migration in a three-dimensional matrix. *J. Cell Sci.* 125, 3661-3670. 10.1242/jcs.103861 22467866 PMC3445327

[JCS260827C77] Shikanai, M., Nishimura, Y. V., Sakurai, M., Nabeshima, Y.-I., Yuzaki, M. and Kawauchi, T. (2018). Caveolin-1 promotes early neuronal maturation via caveolae-independent trafficking of N-cadherin and L1. *iScience* 7, 53-67. 10.1016/j.isci.2018.08.014 30267686 PMC6135901

[JCS260827C78] Takeda, T., Robinson, I. M., Savoian, M. M., Griffiths, J. R., Whetton, A. D., McMahon, H. T. and Glover, D. M. (2013). Drosophila F-BAR protein Syndapin contributes to coupling the plasma membrane and contractile ring in cytokinesis. *Open Biol.* 3, 130081. 10.1098/rsob.130081 23926047 PMC3758542

[JCS260827C79] Takei, K., Slepnev, V. I., Haucke, V. and De Camilli, P. (1999). Functional partnership between amphiphysin and dynamin in clathrin-mediated endocytosis. *Nat. Cell Biol.* 1, 33-39. 10.1038/9004 10559861

[JCS260827C80] Theveneau, E., Marchant, L., Kuriyama, S., Gull, M., Moepps, B., Parsons, M. and Mayor, R. (2010). Collective chemotaxis requires contact-dependent cell polarity. *Dev. Cell* 19, 39-53. 10.1016/j.devcel.2010.06.012 20643349 PMC2913244

[JCS260827C81] Thiery, J. P. and Sleeman, J. P. (2006). Complex networks orchestrate epithelial-mesenchymal transitions. *Nat. Rev. Mol. Cell Biol.* 7, 131-142. 10.1038/nrm1835 16493418

[JCS260827C82] Vehlow, A., Soong, D., Vizcay-Barrena, G., Bodo, C., Law, A.-L., Perera, U. and Krause, M. (2013). Endophilin, lamellipodin, and mena cooperate to regulate F-actin-dependent EGF-receptor endocytosis. *EMBO J.* 32, 2722-2734. 10.1038/emboj.2013.212 24076656 PMC3801443

[JCS260827C83] Wang, Y., Cao, H., Chen, J. and McNiven, M. A. (2011). A direct interaction between the large GTPase dynamin-2 and FAK regulates focal adhesion dynamics in response to active Src. *Mol. Biol. Cell* 22, 1529-1538. 10.1091/mbc.e10-09-0785 21411625 PMC3084675

[JCS260827C84] Wang, X., Enomoto, A., Asai, N., Kato, T. and Takahashi, M. (2016). Collective invasion of cancer: perspectives from pathology and development. *Pathol. Int.* 66, 183-192. 10.1111/pin.12391 26897041

[JCS260827C85] Wheelock, M. J., Shintani, Y., Maeda, M., Fukumoto, Y. and Johnson, K. R. (2008). Cadherin switching. *J. Cell Sci.* 121, 727-735. 10.1242/jcs.000455 18322269

[JCS260827C86] Woodings, J. A., Sharp, S. J. and Machesky, L. M. (2003). MIM-B, a putative metastasis suppressor protein, binds to actin and to protein tyrosine phosphatase delta. *Biochem. J.* 371, 463-471. 10.1042/bj20021962 12570871 PMC1223315

[JCS260827C87] Xiao, K., Allison, D. F., Buckley, K. M., Kottke, M. D., Vincent, P. A., Faundez, V. and Kowalczyk, A. P. (2003a). Cellular levels of p120 catenin function as a set point for cadherin expression levels in microvascular endothelial cells. *J. Cell Biol.* 163, 535-545. 10.1083/jcb.200306001 14610056 PMC2173638

[JCS260827C88] Xiao, K., Allison, D. F., Kottke, M. D., Summers, S., Sorescu, G. P., Faundez, V. and Kowalczyk, A. P. (2003b). Mechanisms of VE-cadherin processing and degradation in microvascular endothelial cells. *J. Biol. Chem.* 278, 19199-19208. 10.1074/jbc.M211746200 12626512

[JCS260827C89] Yagi, T. and Takeichi, M. (2000). Cadherin superfamily genes: functions, genomic organization, and neurologic diversity. *Genes Dev.* 14, 1169-1180. 10.1101/gad.14.10.1169 10817752

[JCS260827C90] Yamada, K. M. and Sixt, M. (2019). Mechanisms of 3D cell migration. *Nat. Rev. Mol. Cell Biol.* 20, 738-752. 10.1038/s41580-019-0172-9 31582855

[JCS260827C91] Yamamoto, H., Sutoh, M., Hatakeyama, S., Hashimoto, Y., Yoneyama, T., Koie, T., Saitoh, H., Yamaya, K., Funyu, T., Nakamura, T. et al. (2011). Requirement for FBP17 in invadopodia formation by invasive bladder tumor cells. *J. Urol.* 185, 1930-1938. 10.1016/j.juro.2010.12.027 21421245

[JCS260827C92] Yang, Y., Zheng, H., Zhan, Y. and Fan, S. (2019). An emerging tumor invasion mechanism about the collective cell migration. *Am. J. Transl. Res.* 11, 5301-5312. 31632511 PMC6789225

[JCS260827C93] Zhang, Y., Nolan, M., Yamada, H., Watanabe, M., Nasu, Y., Takei, K. and Takeda, T. (2016). Dynamin2 GTPase contributes to invadopodia formation in invasive bladder cancer cells. *Biochem. Biophys. Res. Commun.* 480, 409-414. 10.1016/j.bbrc.2016.10.063 27771248

[JCS260827C94] Zimu, Z., Jia, Z., Xian, F., Rui, M., Yuting, R., Yuan, W., Tianhong, W., Mian, M., Yinlong, L. and Enfang, S. (2021). Decreased expression of PACSIN1 in brain glioma samples predicts poor prognosis. *Front. Mol. Biosci.* 8, 696072. 10.3389/fmolb.2021.696072 34422904 PMC8375027

[JCS260827C95] Zisis, T., Brückner, D. B., Brandstätter, T., Siow, W. X., d'Alessandro, J., Vollmar, A. M., Broedersz, C. P. and Zahler, S. (2022). Disentangling cadherin-mediated cell-cell interactions in collective cancer cell migration. *Biophys. J.* 1, 44-60. 10.1016/j.bpj.2021.12.006 PMC8758422 34890578

