# Supplementary material for: Pacsin 2-dependent N-cadherin internalization regulates the migration behaviour of malignant cancer cells
Source: J Cell Sci. 2023 May 31;136(10):jcs260827. doi: 10.1242/jcs.260827 (PMC10281519; doi:10.1242/jcs.260827)
Supplement: Supplementary information [file joces-136-260827-s1.pdf]

Fig. S1 Wint et al.

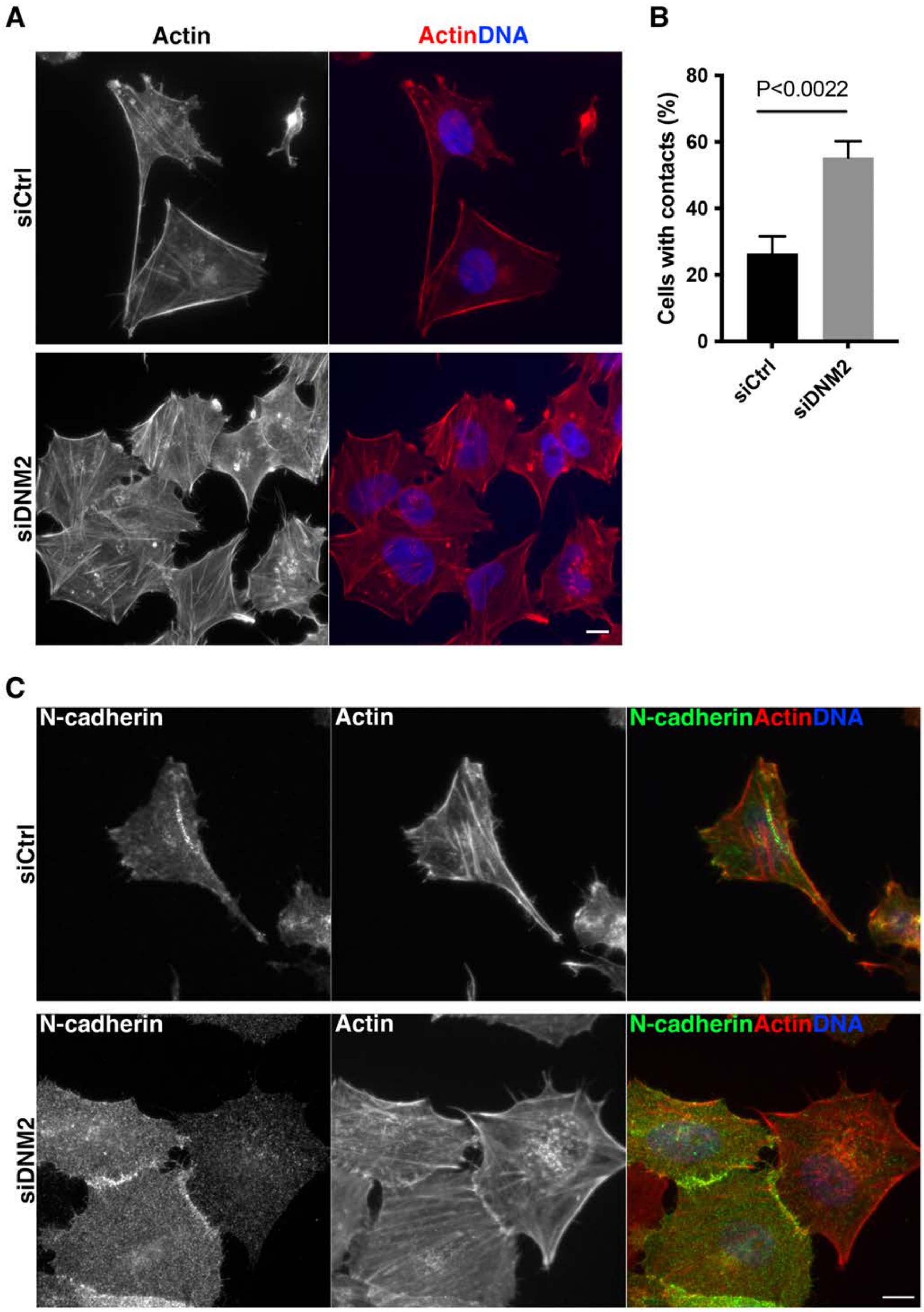

Journal of Cell Science • Supplementary information

Fig. S1. Depletion of dynamin 2 induces N-cadherin-rich bridges between contacting cells. (A) Immunofluorescence micrographs of F-actin (red) and its merged images with DNA (blue) in control RNAi (siCtrl) or dynamin 2 RNAi (siDNM2) cells. The scale bar is 10 µm. (B) Quantitation of the relative number of cells with cell-cell contacts for control RNAi cells (siCtrl) or dynamin 2 RNAi cells (siDNM2). Data are means ± SD ($n \geq 130$ cells, $N=3$) (C) Immunofluorescence micrographs of control RNAi cells (siCtrl) or dynamin 2 RNAi cells (siDNM2) stained for endogenous N-cadherin (green), F-actin (red) and their merged images with DNA (blue). The scale bar is 10 µm.

Fig.S2 Wint et al.

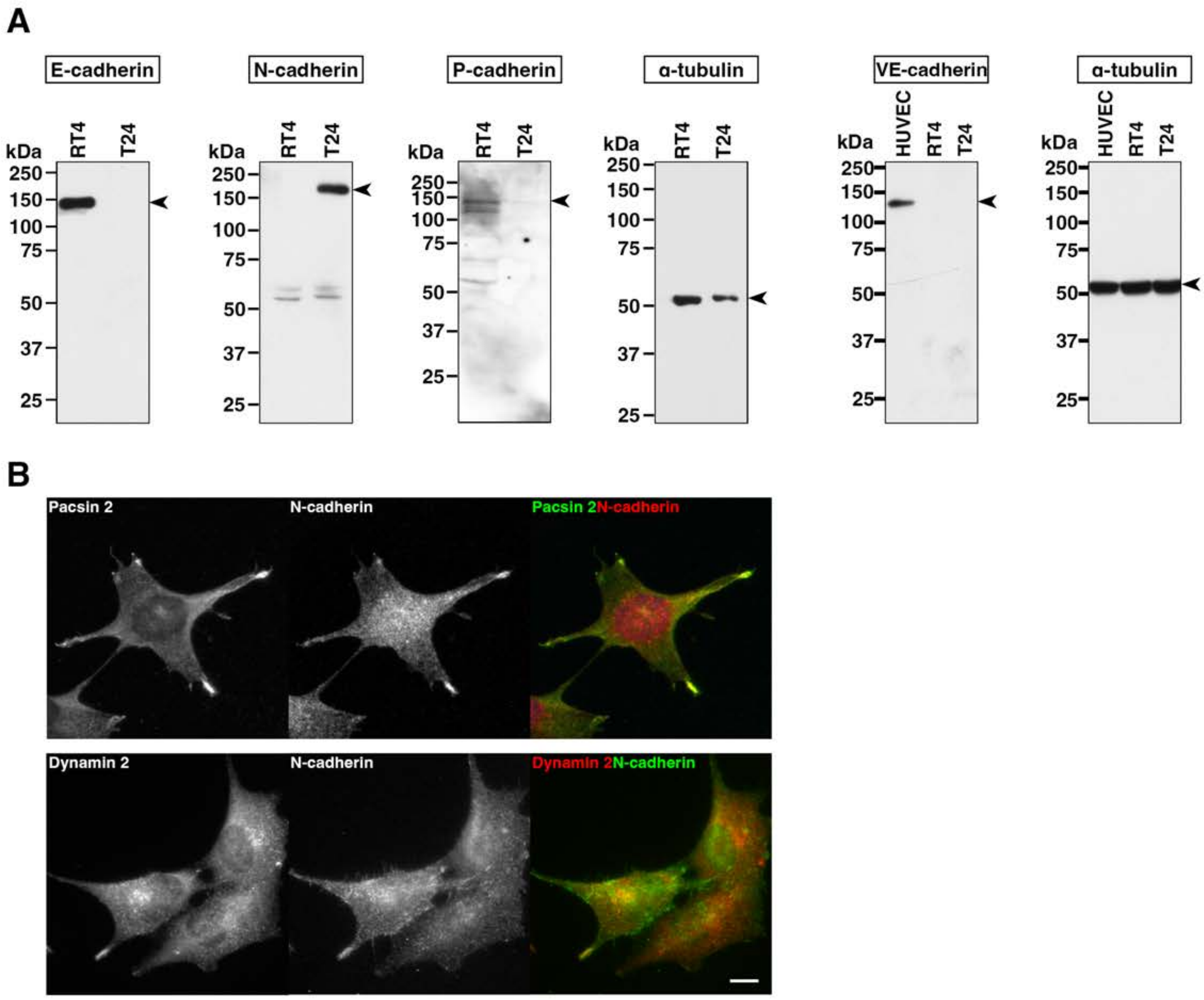

Fig. S2. N-cadherin colocalizes with pacsin 2 and dynamin 2 at the cell periphery in T24 cells. (A) Immunoblot analysis of endogenous E-, N- and P-cadherins in RT4 or T24 cells, and VE-cadherin in RT4, T24 or HUVEC cells together with an internal control (α-tubulin). (B) Immunofluorescence micrographs of endogenous pacsin 2 (green) with endogenous N-cadherin (red) and their merged images (upper panel) or endogenous N-cadherin (green), endogenous dynamin 2 (red) and their merged images (lower panel). The scale bar is 10 μm.

Fig.S3 Wint et al.

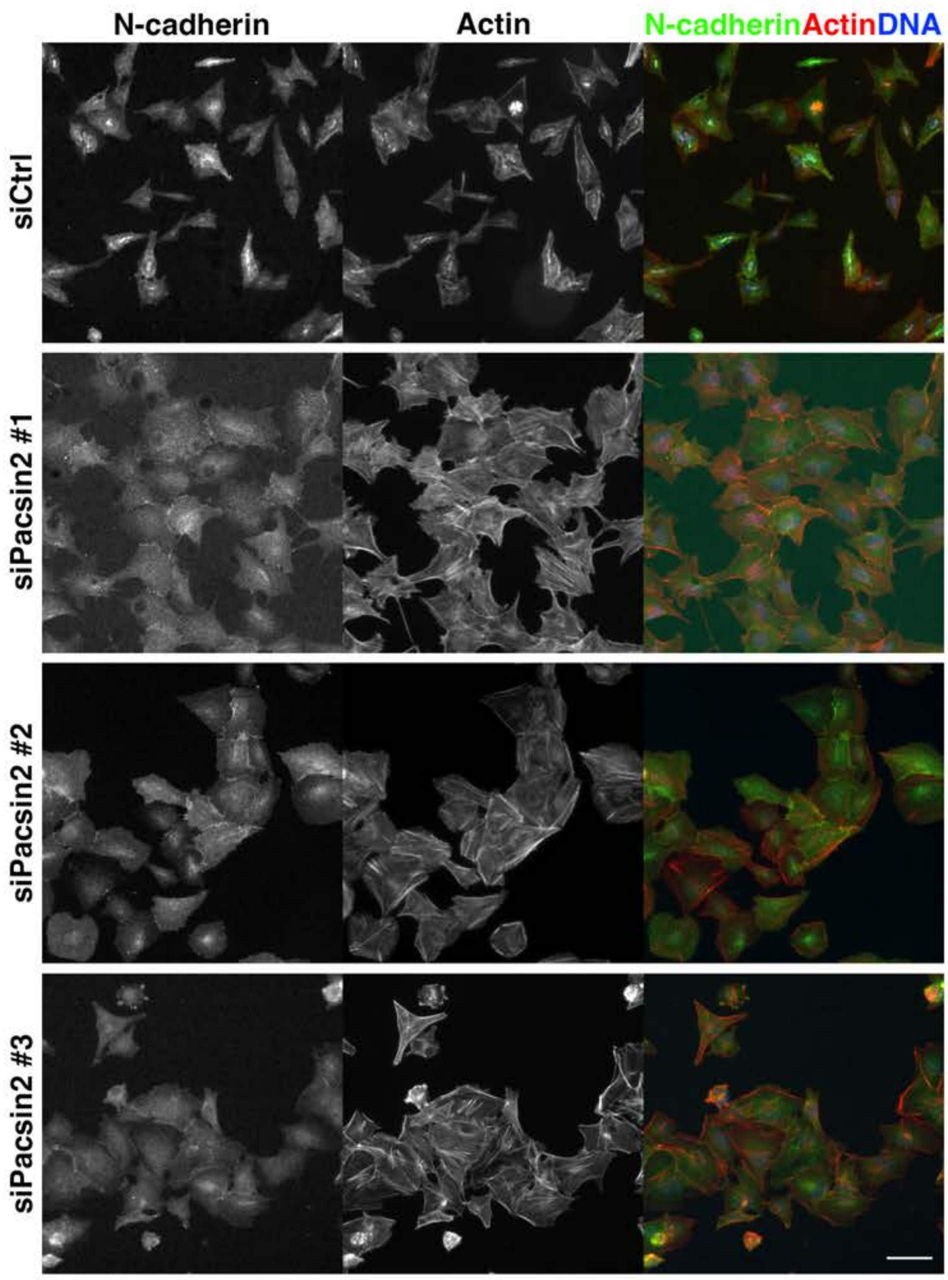

Fig. S3. Induction of cell-cell contacts by pacsin 2 RNAi in T24 cells. Low magnification images of N-cadherin (N-cadherin), F-actin (actin) and their merged images with DNA. The scale bar is 50 μm.

Journal of Cell Science • Supplementary information

Fig.S4 Wint et al

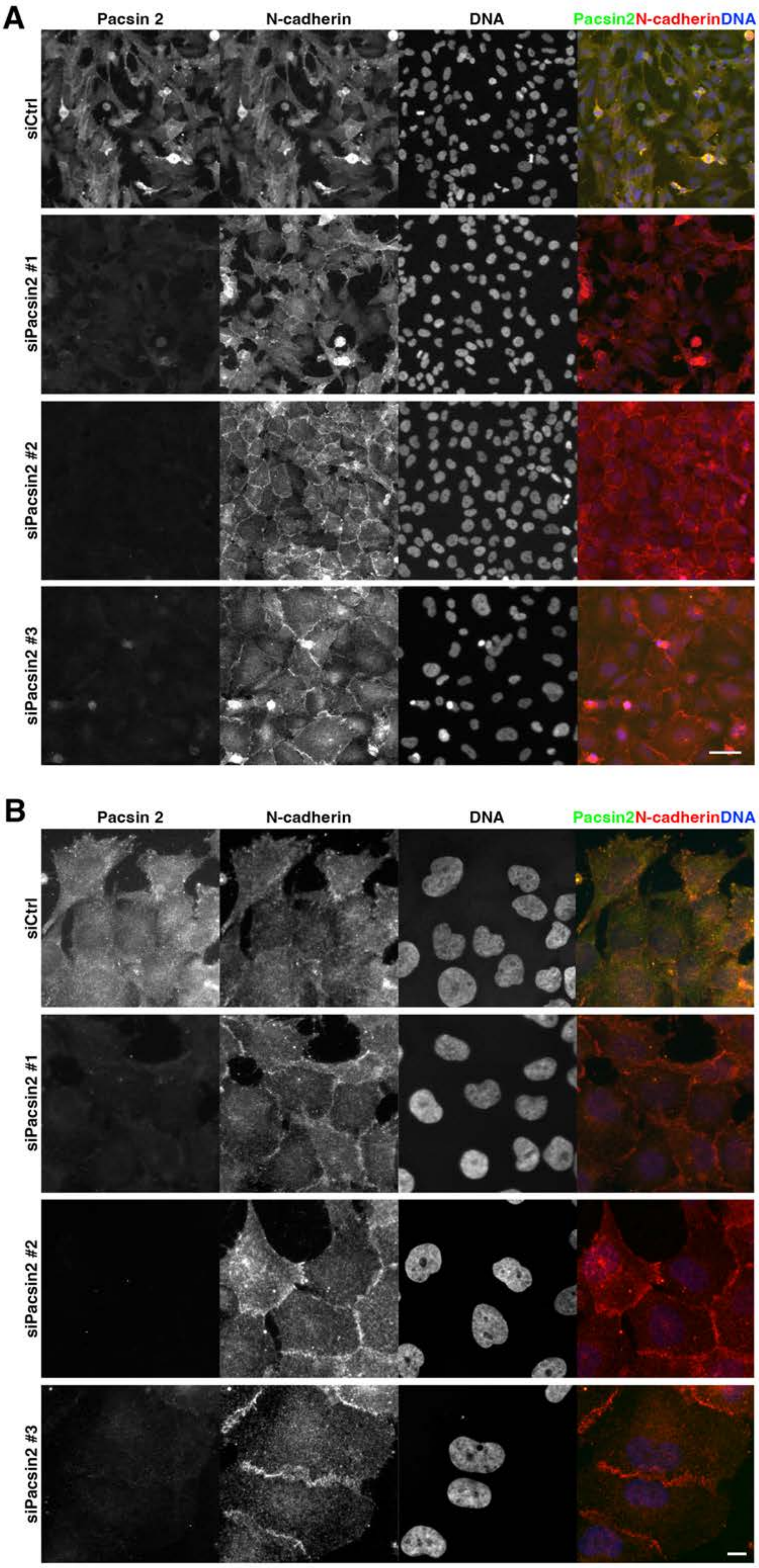

Fig. S4 N-cadherin accumulation is induced by the depletion of pacsin 2 in T24 cells. (A) Low and (B) high magnification immunofluorescence images of pacsin 2, N-cadherin, DNA and their merged images in control RNAi cells (siCtrl) and pacsin 2 RNAi cells (siPacsin2 #1, #2 and 3) in a densely plated condition. Scale bars are 50 μm (A) and 10 μm (B).

Journal of Cell Science • Supplementary information

Fig. S5 Wint et al.

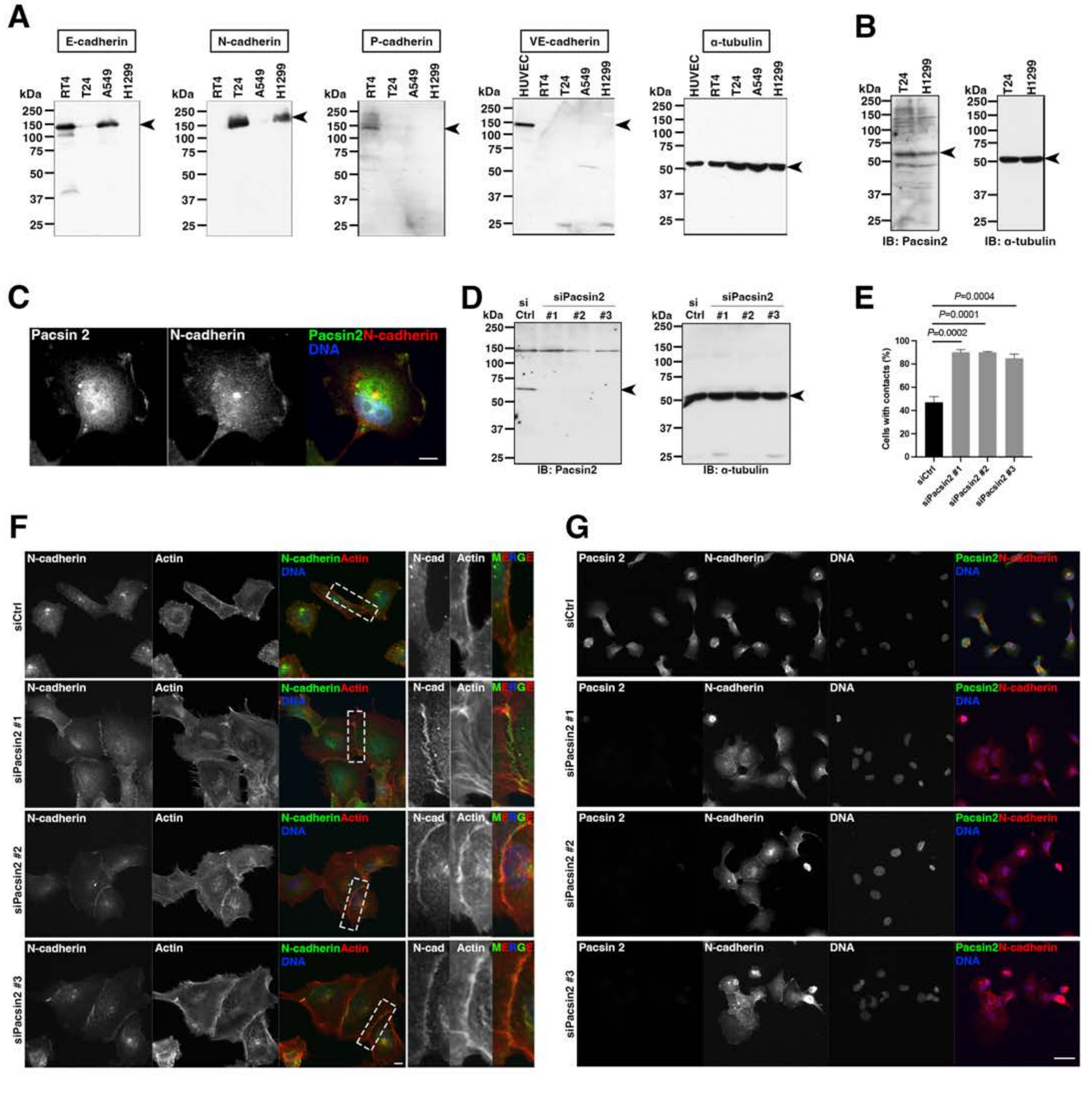

Journal of Cell Science • Supplementary information

Fig. S5. Depletion of pacsin 2 induces N-cadherin-rich cell junctions in H1299 cells. (A) Expression profiles of cadherin isoforms in A549 and H1299 cells in comparison to RT4, T24 or HUVEC cells. (B) Expression of pacsin 2 in H1299 cells. Immunoblot analysis of pacsin 2 in H1299 cells in comparison to T24 is shown together with an internal control (α-tubulin). (C) Immunofluorescence micrographs stained for pacsin 2 (green), N-cadherin (red) and their merged images with DNA (blue) in H1299 cells. The scale bar is 10 µm. (D) Immunoblot analysis of cell extract from control RNAi cells (siCtrl) or pacsin 2 RNAi cells (siPacsin 2 #1, #2 and #3) using antibodies against pacsin 2 (IB: Pacsin 2) or tubulin as an internal control (IB: tubulin). (E) Depletion of pacsin 2 induces cell-cell contacts in H1299 cells. Quantitation of cells with cell contacts in control RNAi cells (siCtrl) or pacsin 2 RNAi cells (siPacsin 2 #1, #2 and #3). Data are means ± SD ($n \geq 229$ cells, N=3). (F) Immunofluorescence micrographs of control RNAi cells (siCtrl) and pacsin 2 RNAi cells (siPacsin 2 #1, #2 and #3) stained for endogenous N-cadherin (green), F-actin (red) and their merged images with DNA (blue). Enlarged images show either the cell periphery in control cells or N-cadherin-rich cell-cell contact sites in pacsin 2 RNAi cells (shown in the dashed rectangle). Scale bars are 10 µm. (G) Low magnification images of pacsin 2 (green), N-cadherin (red) and their merged images with DNA (blue). The scale bar is 50 μm.

Journal of Cell Science • Supplementary information

Fig. S6 Wint et al.

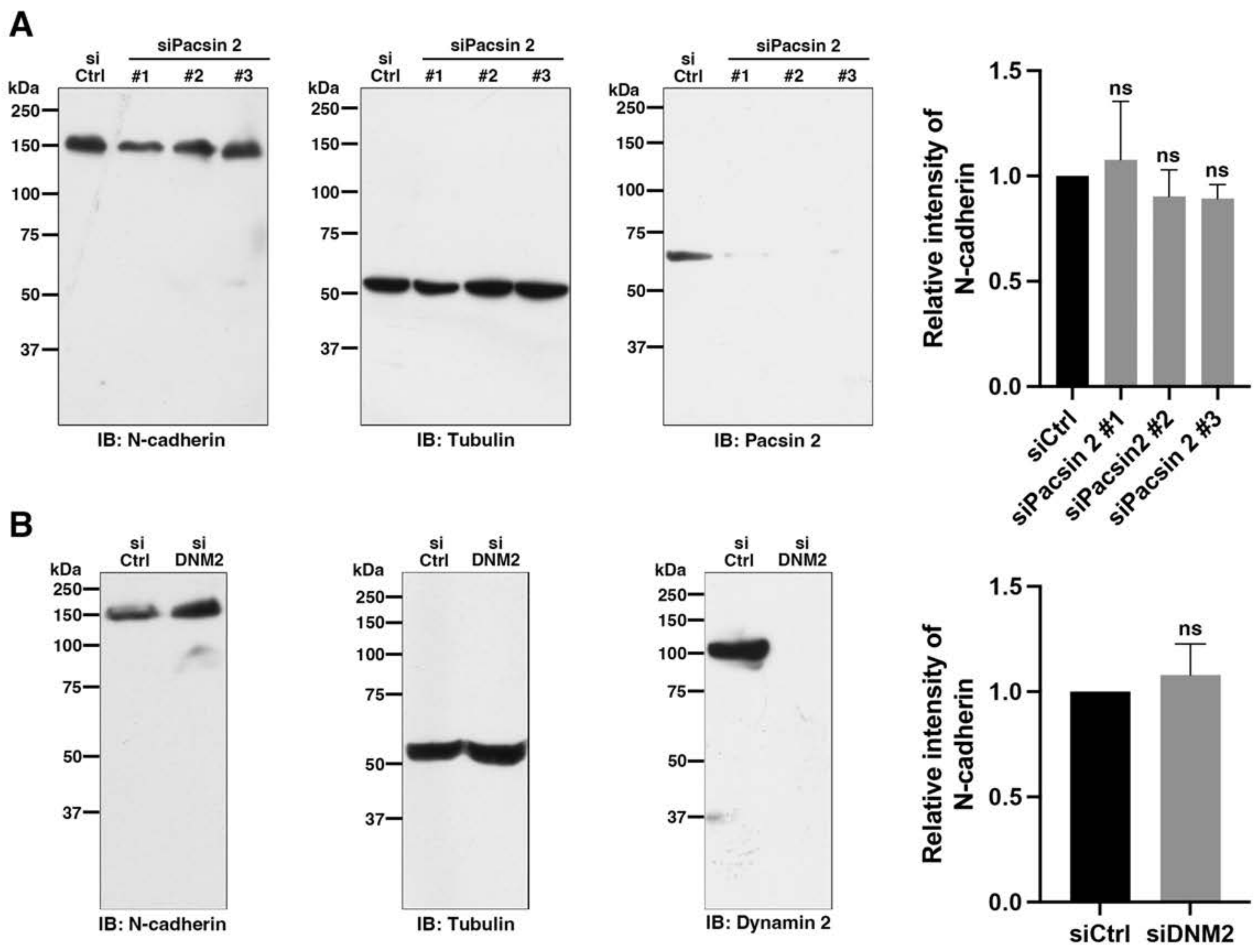

Fig. S6. Expression level of N-cadherin in T24 cells is not affected by depletion of either pacsin 2 or dynamin 2. (A) Immunoblot analyses of cell extract from either control RNAi cells (siCtrl) or pacsin 2 RNAi (siPacsin 2 #1, #2 and #3) cells using antibodies against N-cadherin (IB: N-cadherin), pacsin 2 (IB: Pacsin 2) or αTubulin (IB: Tubulin) as an internal control. Quantitation of N-cadherin levels relative to α-tubulin in control RNAi cells (siCtrl) or pacsin 2 RNAi cells (siPacsin 2 #1, #2 and #3) are also shown. Data are means ± SD (N=3) (B) Immunoblot analysis of cell extract from either control RNAi cells (siCtrl) or dynamin 2 RNAi (siDNM2) cells using antibodies against N-cadherin (IB: N-cadherin), dynamin 2 (IB: dynamin 2) or αTubulin (IB: Tubulin) as an internal control. Quantitation of N-cadherin levels relative to -tubulin in control RNAi (siCtrl)or dynamin 2 RNAi (siDNM2) cells are also shown. Data are means ± SD (N=3).

Fig.S7 Wint et al.

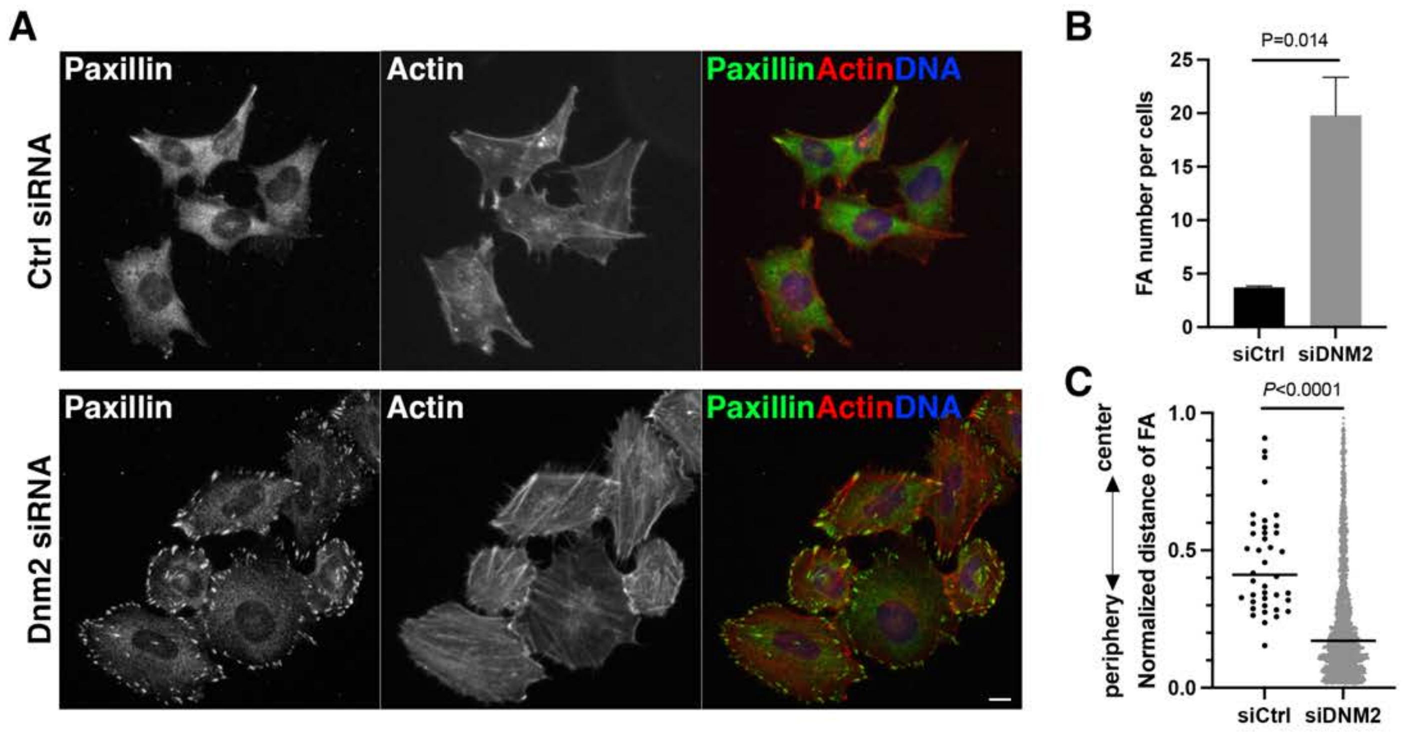

Fig. S7. Depletion of dynamin 2 induces an elevated number of focal adhesions in T24 cells. (A) Immunofluorescence micrographs of Paxillin (green), F-actin (red) and their merged images with DNA (blue) in control RNAi cells (siCtrl) and dynamin 2 RNAi cells (siDNM2). (B) Quantitation of focal adhesions in control RNAi cells (siCtrl) and dynamin 2 RNAi cells (siDNM2). Data are means ± SD (n≥120 cells, N=3). (C) Spatial distribution of focal adhesions (FA) in either control RNAi (siCtrl) or dynamin 2 RNAi (siDNM2) cells. Normalized distance of FA between periphery (0) and centre (1) are shown (control: n≥15 cells; siDNM2:n≥54 cells, N=3). The scale bar is 10 µm.

Journal of Cell Science • Supplementary information

Fig. S8 Wint et al.

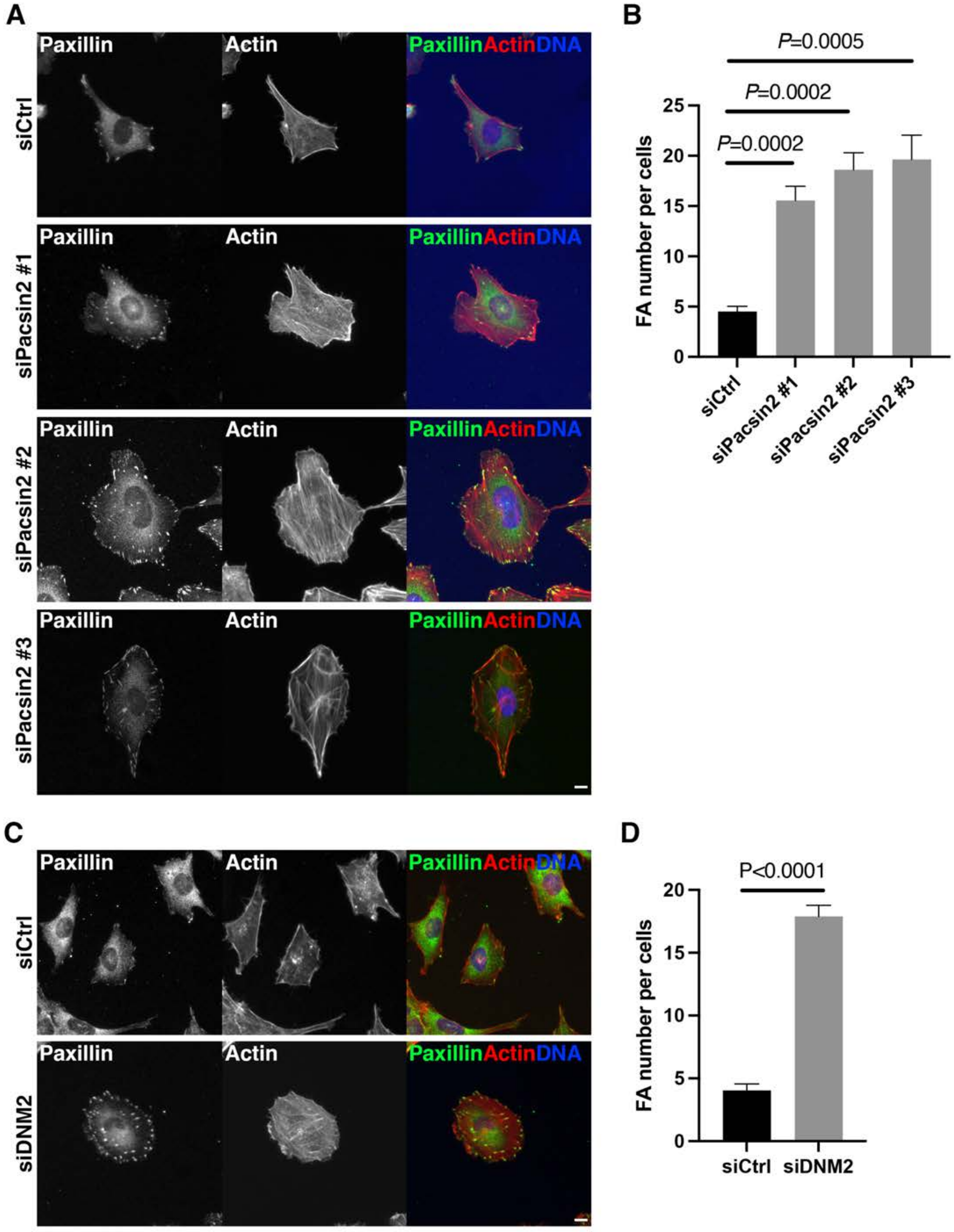

Fig. S8. Depletion of pacsin 2 or dynamin 2 induces an elevated number of focal adhesions in single-cell conditions. (A) Immunofluorescence micrographs of control RNAi cells (siCtrl) and pacsin 2 RNAi cells (siPacsin 2 #1, #2 and #3) stained for a focal adhesion marker paxillin (green), F-actin (red) and their merged images with DNA (blue). (B) Quantitation of focal adhesions in control RNAi cells (siCtrl) and pacsin 2 RNAi cells (siPacsin 2 #1, #2 and #3). Data are means ± SD (n≥100 cells, N=3). (C) Immunofluorescence micrographs of Paxillin (green), F-actin (red) and their merged images with DNA (blue) in control RNAi cells (siCtrl) and dynamin 2 RNAi cells (siDNM2). (D) Quantitation of focal adhesions in control RNAi cells (siCtrl) and dynamin 2 RNAi cells (siDNM2). Data are means ± SD (n≥100 cells, N=3).

Table S1. Primers.

Targets	Primers
Pacsin 2 SH3	5'-GGGGACAAGTTTGTACAAAAAAGCAGGCTGCGGGACGGAAGTGCGA-3'<br>5'-GGGGACCACTTTGTACAAGAAAGCTGGGTTCACTGGATCGCCTCC-3'
N-cadherin Full	5'-GGGGACAAGTTTGTACAAAAAAGCAGGCTGCATGTGCCGGATAGCG-3'<br>5'-GGGGACCACTTTGTACAAGAAAGCTGGGTCGTCATCACCTCCACCATACAT-3'
N-cadherin cytoplasmic domain	5'-CACCATGAAACGCCGGGATAAAG-3'<br>5'-TCAGTCATCACCTCCACC-3'
N-cadherin mutants P818/P821A	5'-GATCGGACCGCATACTGGGCCTCAGCGTGG-3'<br>5'-CCACGCTGAGGCCCAGTATGCGGTCCGATC-3'
N-cadherin mutants P847/850/851A	5'-AGGGAGTCATATGCTGCAGCTGTGGCGTCATTGTCAGCC-3'<br>5'-GGCTGACAATGACGCCACAGCTGCAGCATATGACTCCCT-3'

*Underlined sequences are for BP recombination (Pacsin 2 SH3 and N-cadherin Full) and Blunt TOPO PCR cloning (N-cadherin cytoplasmic domain).

Journal of Cell Science • Supplementary information

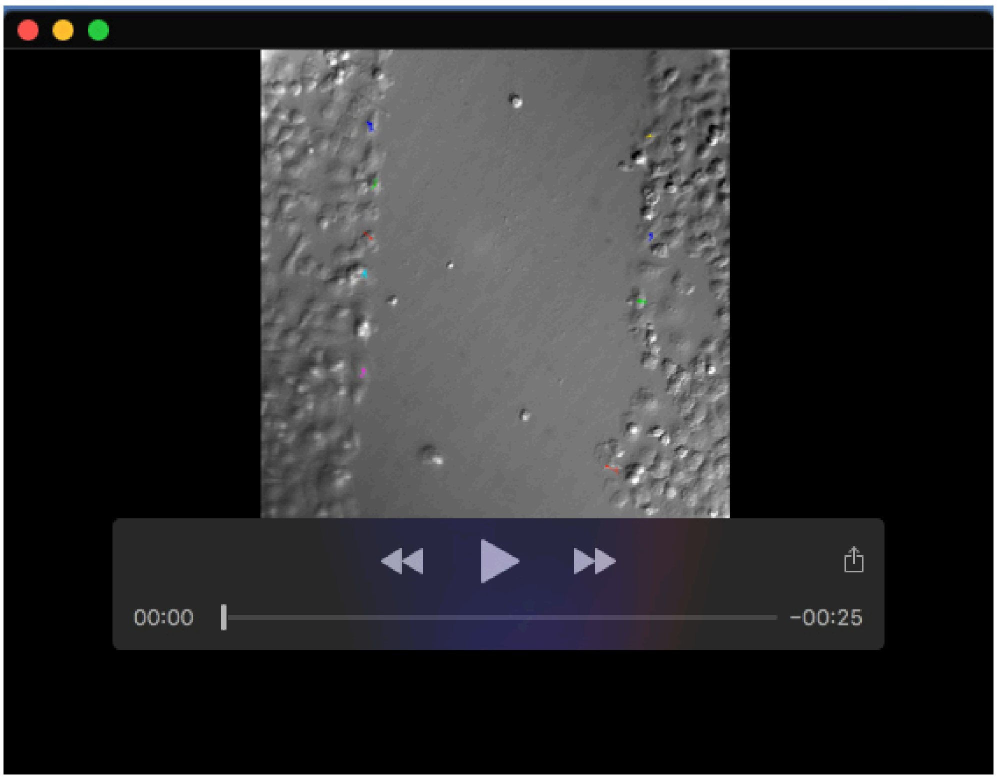

Movie 1. Control RNAi cells in the wound healing assay. Images were obtained every 1min for 6 hours after the start of the wound healing assay. Trajectories of the ten representing cells are shown in different colours.

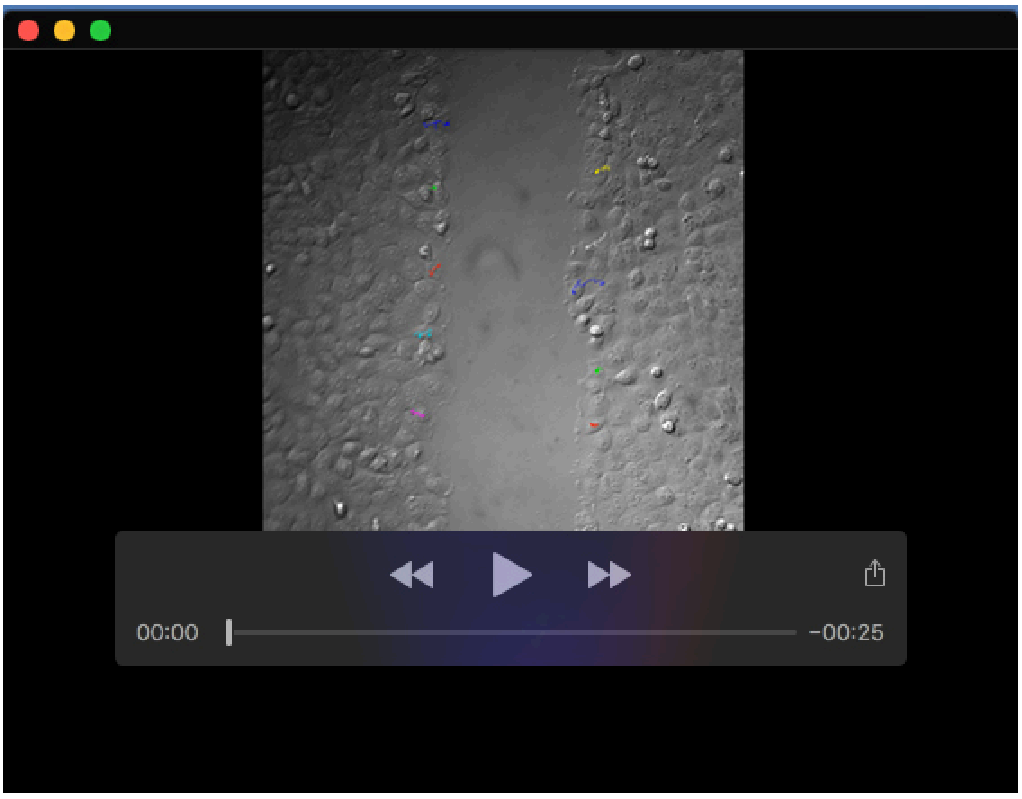

Movie 2. Pacsin 2 RNAi cells in the wound healing assay. Images were obtained every 1min for 6 hours after the start of the wound healing assay. Trajectories of the ten representing cells are shown in different colours.
