# Supplementary material for: Pacsin 2-dependent N-cadherin internalization regulates the migration behaviour of malignant cancer cells
Source: J Cell Sci. 2023 May 31;136(10):jcs260827. doi: 10.1242/jcs.260827 (PMC10281519; doi:10.1242/jcs.260827)
Supplement: Supplementary file 1 [file joces-136-260827_review_history.pdf]

Pacsin 2-dependent N-cadherin internalization regulates the migration behaviour of malignant cancer cells

Haymar Wint, Jianzhen Li, Tadashi Abe, Hiroshi Yamada, Takumi Higaki, Yasutomo Nasu, Masami Watanabe, Kohji Takei and Tetsuya Takeda
DOI: 10.1242/jcs.260827

Editor: Michael Way

Review timeline

Submission to Review Commons:	8 August 2022
Submission to Journal of Cell Science:	22 November 2022
Editorial decision:	19 December 2022
First revision received:	24 February 2023
Editorial decision:	16 March 2023
Second revision received:	12 April 2023
Accepted:	13 April 2023

Reviewer 1

Evidence, reproducibility and clarity

SUMMARY

This study identifies a role for the F-bar (membrane curvature sensing/binding) protein, Pacsin2, in the negative regulation of N-cadherin based cell-cell adhesions, using an siRNA acute knock-down approach in the human bladder T24 cancer cell line (E-cad negative, N- cad positive malignant bladder cancer cell line). Rationale for addressing Pacsin2 loss-of- function in this cell line is based on TCGA database evidence of deep-deletion/mutation in human bladder cancers; specifics are referenced rather than detailed. Consequences of Pacsin2 KD are evaluated by immunoblot analysis of total cell lysates. Consequences for N- cadherin based cell-cell adhesion are inferred from investigator selected fields-of-view showing T24 cells with lesser or greater cell-cell contact formation Potential investigator bias in choosing fields of view or low magnification views are not provided. N-cadherin endocytosis is validated (in the presence of, or under reduced levels of Pacisn2) using classic endocytosis biotinylation-based assay/followed by immunoblotting/quantification of bands. Consequences of this Pacsin2 reduction in T24 cells is enhanced migration (via 2D- scatch wound assay; area migration quantification); individual cell tracking reveals that enhanced wound closure/migration is due to more coordination/ persistence of cells along the wound front). A minimal, in vitro binding assay shows that the SH3-domain of Pacsin2 can directly bind N-cadherin cytodomain. Authors note that the relationship between Pacsin2 and cell-cell adhesion appears complex, as the model presented in this study is opposite to what has been shown for Pacsin2/VE-cadherin function in endothelial cells (where Pacsin2 promotes VE-cadherin cell-cell adhesive function in context of collective cell migration).

Major Comments:

- Are the key conclusions convincing?

The Pacsin2 siRNA KD appears robust by immunoblot analysis. However, the relationship between Pacsin2 KD and reduced N-cadherin internalization could be more robustly/transparently shown. "Representative views" show consequences for Pacsin2 KD on N-cadherin-based cell-cell adhesion. The authors state they find more cell-cell islands with robust contact formation; but the

© 2023. Published by The Company of Biologists under the terms of the Creative Commons Attribution License (https://creativecommons.org/licenses/by/4.0/).

quantification appears subjective; low magnification views would help, paired with Pacsin2 immunostaining. There is also concern that what is being quantified here is due to initial differences in plating density rather than true effect on N-cadherin at cell-cell contacts. It would be helpful to see an image showing densely plated control siRNA and Pacsin2 siRNA KD cells with double-staining for N-cadherin and Pacsin2 to assess effects on N-cadherin localization at junctions. As there is no double-labeling of Pacsin2 and N-cadherin- one cannot readily follow knock-down/N-cadherin changes in single cells, which would better support the Pacsin2 KD/protein reduction/N-cadherin cell-cell contact stabilization relationship.

- Should the authors qualify some of their claims as preliminary or speculative, or remove them altogether?

Claim that Pacsin2 KD impacts cell-cell adhesion via N-cadherin through reduced N- cadherin turnover is somewhat supported, but relationship/data presentation could be more robust/transparent (see #1 above).

- Would additional experiments be essential to support the claims of the paper? Request additional experiments only where necessary for the paper as it is, and do not ask authors to open new lines of experimentation.

Yes- see #1 above.

- Are the suggested experiments realistic in terms of time and resources? It would help if you could add an estimated cost and time investment for substantial experiments.

Request does not seem overly burdensome; possible that the authors cannot detect N- cadherin and Pacsin2 protein at the same time, but if this is the case, the authors should really find another work around (tagged N-cadherin) since it is pretty essential to their study.

- Are the data and the methods presented in such a way that they can be reproduced?

Standard methods used here and method section is reasonably detailed.

- Are the experiments adequately replicated and statistical analysis adequate?

Experimental robustness/field of view transparency and Pacsin2 KD/N-cadherin protein localization views in the same cell are suggested to improve interpretation/study robustness.

Minor comments:

- Specific experimental issues that are easily addressable.

- Are prior studies referenced appropriately?

Rationale for carrying out Pacsin2 KD in this malignant bladder cancer cell line T24 is obliquely referenced along with TCGA data. Would be nice to have authors explicitly state whether Pacsin2 is deeply deleted and undergoes gain or loss of function mutation in these cancers- at least for context.

- Are the text and figures clear and accurate?

Double-labeling of Pacsin2/N-cadherin would be helpful.

- Do you have suggestions that would help the authors improve the presentation of their data and conclusions?

See #1 above

© 2023. Published by The Company of Biologists under the terms of the Creative Commons Attribution License (https://creativecommons.org/licenses/by/4.0/).

CROSS-CONSULTATION COMMENTS

General agreement from all three reviewers on issues related to FOV choice/representativeness (lack of robust quantification methods or transparency thereof), need to see Pascin2 localization at confluency/dense cultures, rigor of antibody specificity (Pascin2).

Significance

Overall- the research question is solid. The overall findings expand what is known about Pacsin2 loss-of-function phenotypes relevant to cell migration. Use of the single T24 bladder cancer cell line (E-cad-; N-cad+) may narrow the overall generality of the model-- it is not clear if what is being learned here is generalizable for N-cadherin over E-cadherin, and/or specific to this single cell system. I'd like to see greater transparency with how the data are presented (lower magnification FOVs; Pacsin2 KD/N-cadherin consequences at the single cell/immunofluorescence level rather than simply whole lysates to ensure Pacsin2 protein level/phenotype relationships. The in vitro evidence that Pacsin2 engages the N-cadherin cytodomain directly (to mediate endocytosis/turnover) is preliminary: binding is only shown between SH3 domain of Pacsin2 and N-cad tail, where typically the cadherin cytodomain is masked by catenins (beta cat and p120ctn). A complete study would mutate candidate interaction sites in N-cadherin and ask whether this mutant form of N-cadherin phenocopies the Pacsin2 KD. Having this data would make the study highly impactful, but this reviewer understands how much more work this is. Nonetheless, I see the Pacsin2 KD/effect on N- cadherin turnover/adhesion/migration a sufficient advance without this added information, so long as data robustness issues can be addressed below.

Reviewer 2

Evidence,reproducibility and clarity

Summary:

The authors identify Pacsin 2 playing a role in the regulation of collective cell migration. Silencing the expression of Pacsin 2 inhibits the endocytosis of N-cadherin, which can now stimulate cell-cell contacts and enhance the directionality of cell movement during wound healing.

Major comments:

This is potentially interesting finding, in particular the role of Pacsin controlling endocytosis of N-cadherin. However, in general the results are still preliminary in my opinion and this study would benefit from CTRL experiments and quantification of some of the results.
For example, in Figure 1, the authros conclude that Pacsin 2 does not localizeto invadopodia based on the images shown.

First, a single cell is shown in each panel with no quantification.

Second, not all staining positive for cortactin or dynamin represents an invadopodia structure. It is not unusual to see punctate staining in cells that is cortactin and dynamin positive and may represent vesicles.

Third, not all cells form invadopodia at the same time, so showing one cell should not be sufficient evidence to draw conclusions.

A definitive proofof invadopodia formation would be to use a matrix degradation assay. Finally, it would have been nice to use the Pacsin 2 siRNA cells to validate the specificity of the antibodies.
In Figure 3, only 10 cells/exp are quantified. Is this a sufficient number fir statistical significance? Also, how were these cells selected? More details in the Methods sections are needed
In addition, there is some overlap with key Pacsin2 articles in the literature that were not cited in the manuscripts, in particular the publications that show Pacsin 2 is involved in collective cell migration (de Kreuk 2011; DOI: 10.1242/jcs.080630. Other key references not cited: Malinova 2021 (DOI: 10.1038/s41467-021-22873-y), de Kreuk 2012 (DOI: 10.1074/jbc.M112.391078) and Meng 2011 (DOI: 10.4161/cc.10.1.14243)

© 2023. Published by The Company of Biologists under the terms of the Creative Commons Attribution License (https://creativecommons.org/licenses/by/4.0/).

In my opinion, Figure 5 shows the most interesting results. However, I have several question/comments regarding these results: a) most figures show one or two cells positive for N-cadherin at cell junctions, but there is no quantification to show what is the difference between CTRL and KD cell lines. The text states that 34% of CTRL cells form cell-cell contacts. Are these contacts mediated by N-cadherin? A picture of cell-cell contacts found in CTRL cellwould be informative. The other question not answered/quantified is whether dynamin KD cells also increase the number of cells that form cell-cell junctions.
The images in Figure 6 are quite striking. However, there is not really a discussion of what does this mean in terms of inhibiting endocytosis and keeping N-cadherin at the plasma membrane. Since the connection between cellslook so different, the same question asked above is relevant, i.e. are the cell-cell junctions in CTRL cells also mediated by N-cadherin.

- The interaction between N-cadherin and Pacsin 2 is only shown in vitro and using fragments of each protein. The manuscript would be strengthened inf the interaction is confirmed with endogenous proteins.

- The finding regarding FA numbers is also interested but somehow disconnected to the rest of the story. How does the inhibition of N-cadherin endocytosis regulate FA turnover? How does it affect the directionality of migration and why, as it would be predicted, it does not slow down migration? Also, the Pacsin 2 KD phenotype shows more FA at the center of the cells (at least in the images shown) whereas for dynamin KD they seem to localize at the periphery of the cells. Based o those pictures alone, the phenotypes appear to be different.

To be able to confirm that, quantification of the position of the FA regarding the edge of the cell (distance) should be quantified (or alternative % of FA at the periphery vs center of the cell).

- Some more details in the methods would be useful, e.g. number of cells plated, quantification of tracks during wound healing, how cells were selected, how minimum N- number was determined, what type of statistical analysis was performed.

Minor comments:

- I suggest merging Fig 1 with Fig S1

- In FigS1, without a positive CTRL, the specificity of Pacsin 3 antibody cannot be confirmed. Similarly, in Fig S3 for VE-cadherin and P-cadherin (i.e. is there no P-cadherin in these cells or the Ab did not work?)

- In Fig 4 it looks like some of the KD cells have a larger area. Since Pacsin 2 has been associated with spreading, it would be important to measure whether in these cells spreading is also affected.

Significance

- The findings are of interesting but not completely unexpected as Pacsin 2 has been associated with endocytosis in several previous publications. Additionally, it has already been shown more than once that Pacsin 2 plays a role in the regulation of collective cell migration. As it is, the results are preliminary in my opinion and may need additional experiments and controls to better support the conclusions.

- As mentioned above, there is some overlap with previous literature, with many of the key articles not referenced here.

- My field of expertise is in cell adhesion, migration and invasion. Also in Rho GTPases signaling and membrane traffic.

© 2023. Published by The Company of Biologists under the terms of the Creative Commons Attribution License (https://creativecommons.org/licenses/by/4.0/).

Reviewer 3

Evidence, reproducibility and clarity

• The authors show that pacsin2 controls N-cadherin in T25 cells. Is this function of Pacsin2 conserved for other N-cadherin expressing cell types?
• In follow-up of that question, in endothelial cells Pacsin2 has been shown to control the internalization of VE-cadherin, and that this trafficking function of Pacsin2 (via the recruitment of the recycling mediators EHD4 and MICAL-L1) is in fact needed to drive directed collective cell migration in scratch assays (Malinova et al, 2021; PMID: 33972531). The authors are asked to discuss how those findings might relate to the finding that depletion of Pacsin2 in fact promotes collective cell migration in T25 cells and thus has the opposite effect on N-cadherin trafficking. What mechanism might underlie this?

• Please correct adherence junctions to adherens junctions.

• Where does pacsin2 localize in confluent T25 cell cultures?

• The relocation/trafficking of N-cadherin might depend on the presence of other classical cadherins. It would be important to know how the depletion of pacsin2 affects the surface levels of all expressed classical cadherins.

• Figure 5; more confluent cultured controls cells should be shown to be able to compare AJ formation in siControl vs. siPacsin2. This also accounts for the other figures in which cell clustering was investigated.

• Is the effect of focal adhesion formation specific for pacsin2, or might it depend on the level of junction formation? What happens to FAs in single cell conditions?

Significance

The findings by Wint et al uncover a role for pacsin2 in the organization of junctions in T25 bladder cancer cells and in cell migration. This is on its own an original finding. The impact of the findings might be larger if confirmation in another N-cadherin expressing cell type can confirm whether this is a conserved function. Also the discussion of contrasting roles for pacsin2 in other cell types might raise the impact, or better understand the context, of the current findings

---

<u>Author response to reviewers' comments</u>

1. General Statements [optional]
   Thank you very much for inviting us to submit our preliminary revision for the Review Commons Refereed Preprint #RC-2022-01620 entitled “Pacsin 2-dependent N-cadherin internalization regulates the migration behaviour of malignant cancer cells”. We are happy to address major and minor points raised by three reviewers and have revised the manuscript accordingly. Specific answers to each reviewer’s comments are as follows.

2. Description of the planned revisions

<u>Reviewer #1</u>

1. *Overall- the research question is solid. The overall findings expand what is known about Pacsin2 loss-of-function phenotypes relevant to cell migration. Use of the single T24 bladder cancer cell line (E-cad-; N-cad+) may narrow the overall generality of the model- it is not clear if what is being learned here is generalizable for N-cadherin over E- cadherin, and/or specific to this single cell system.*

<u>Reviewer #3</u>

1. *The authors show that pacsin2 controls N-cadherin in T25 cells. Is this function of Pacsin2 conserved for other N-cadherin expressing cell types?*
2. *The findings by Wint et al uncover a role for pacsin2 in the organization of junctions*

© 2023. Published by The Company of Biologists under the terms of the Creative Commons Attribution License (https://creativecommons.org/licenses/by/4.0/).

in T25 bladder cancer cells and in cell migration. This is on its own an original finding. The impact of the findings might be larger if confirmation in another N-cadherin expressing cell type can confirm whether this is a conserved function. Also the discussion of contrasting roles for pacsin2 in other cell types might raise the impact, or better understand the context, of the current findings.

We thank Reviewers #1 and #3 for their comments. To assess the generality of the model, other E-cad-; N-cad+ cancer cell lines (e.g. HeLa or H1299) will be used to determine if the pacsin 2-dependent endocytosis of N-cadherin is conserved.

<u>Reviewer #2</u>
<u>Minor comments</u>

1. *In FigS1, without a positive CTRL, the specificity of Pacsin 3 antibody cannot be confirmed. Similarly, in Fig S3 for VE-cadherin and P-cadherin (i.e. is there no P- cadherin in these cells or the Ab did not work?)*

   We thank Reviewer #2 for the comments. The specificity of P-cadherin antibody was confirmed using total cell extract from RT4 (new Fig. S2). To confirm the specificity of pacsin 3 (new Fig. 1, A) or VE-cadherin (new Fig. S2) antibodies, either purified proteins or total cell extract of HEK cells transfected with respective expression constructs will be used in full revision of the manuscript.

3. Description of the revisions that have already been incorporated in the transferred manuscript

<u>Reviewer #1</u>
<u>Major comments</u>

1. *The Pacsin2 siRNA KD appears robust by immunoblot analysis. However, the relationship between Pacsin2 KD and reduced N-cadherin internalization could be more robustly/transparently shown. "Representative views" show consequences for Pacsin2 KD on N-cadherin-based cell-cell adhesion. The authors state they find more cell-cell islands with robust contact formation; but the quantification appears subjective; low magnification views would help, paired with Pacsin2 immunostaining.*

   We thank Reviewer #1 for raising this issue. To probe the relationship between pacsin2 KD and reduced N-cadherin internalization more robustly and transparently, low magnification views of cell-cell contact formation by pacsin 2 KD cells are shown in the new Fig. S4. In these low magnification images, control RNAi cells are dispersedly distributed (Fig. S4, siCtrl), whereas pacsin 2 RNAi cells form more “cell-cell islands” (Fig. S4, siPacsin 2, #1, #2 and #3). Since pacsin2 KD appeared robust not only in immunoblot analysis (Fig. 2C) but also in immunostaining (new Fig. S5, A and B), immunostaining images for N-cadherin, Actin and their merged images with DNA are shown in new Fig. S4 to complement high magnification images in Fig. 5.

2. *There is also concern that what is being quantified here is due to initial differences in plating density rather than true effect on N-cadherin at cell-cell contacts. It would be helpful to see an image showing densely plated control siRNA and Pacsin2 siRNA KD cells with double-staining for N-cadherin and Pacsin2 to assess effects on N-cadherin localization at junctions. As there is no double-labeling of Pacsin2 and N-cadherin- one cannot readily follow knock-down/N-cadherin changes in single cells, which would better support the Pacsin2 KD/protein reduction/N-cadherin cell-cell contact stabilization relationship.*

   We also thank Reviewer #1 for the comments. We show densely plated control and pacsin2 RNAi cells with double-staining for N-cadherin and pacsin2 to assess the effects of pacsin 2 KD on N-cadherin localization at junctions both in low and high magnification (new Fig. S5, A and B, respectively). Consistent with immunoblot analysis (Fig. 2C), pacsin2 KD appeared robust as shown in immunostaining (new Fig. S5, A and B). In densely plated pacsin 2 RNAi cells, N-cadherin was accumulated at the cell-cell contact sites (new Fig. S5, A and B, siPacsin2 #1, 2 and 3) in a similar manner as sparsely plated pacsin 2 RNAi cells (Fig. 5). In contrast, N-cadherin was not accumulated at the cell-cell contact sites in control RNAi cells even in a densely plated condition (new Fig. S5, A and B, siCtrl). These data strongly suggest that N-cadherin accumulation

© 2023. Published by The Company of Biologists under the terms of the Creative Commons Attribution License (https://creativecommons.org/licenses/by/4.0/).

at the cell-cell contact sites is caused by the true effect of pacsin 2 KD on N-cadherin but not due to initial differences in plating density.

Minor Comments

1. *Rationale for carrying out Pacsin2 KD in this malignant bladder cancer cell line T24 is obliquely referenced along with TCGA database. Would be nice to have authors explicitly state whether Pacsin2 is deeply deleted and undergoes gain or loss of function mutation in these cancers- at least for context.*

We thank Reviewer #1 for suggesting to rationalize a reason for carrying out pacsin 2 KD in the malignant bladder cancer cell line. We now explicitly state deep deletions or mutations in pacsin 2 gene that have been identified in bladder cancer cells and other cancer cell types including ovarian and breast cancer cells both in the introduction and discussion section.

2 *The in vitro evidence that Pacsin2 engages the N-cadherin cytodomain directly (to mediate endocytosis/turnover) is preliminary: binding is only shown between SH3 domain of Pacsin2 and N-cad tail, where typically the cadherin cytodomain is masked by catenins (beta cat and p120ctn). A complete study would mutate candidate interaction sites in N-cadherin and ask whether this mutant form of N-cadherin phenocopies the Pacsin2 KD. Having this data would make the study highly impactful, but this reviewer understands how much more work this is. Nonetheless, I see the Pacsin2 KD/effect on N-cadherin turnover/adhesion/migration a sufficient advance without this added information, so long as data robustness issues can be addressed below.*

We agree with Reviewer #1 for the preliminary *in vitro* evidence about the direct engagement of pacsin 2 in endocytosis/turnover of N-cadherin. To make the data more impactful, putative pacsin 2 interaction sites in N-cadherin cytodomain (PxxP motifs) were mutated and their interaction with pacsin 2 SH3 domain was examined (Fig. 8). In a GST pull-down assay, wild type GFP-tagged N-cadherin cytodomain efficiently interacts with GST-tagged pacsin 2 SH3 domain (new Fig. 8, C, Wt). In contrast, the interaction of mutant forms of GFP-tagged N-cadherin cytodomain with pacsin 2 SH3 domain was suppressed (Fig.8, C, P818/821A, P847/850A or P847/850/851A). Interestingly, the interaction became almost undetectable when N-cadherin cytodomain with mutations in the two PxxP motifs (Fig. 8, C, P818/821A+P847/850A or P818/821A+P847/850/851A). These results strongly suggest that pacsin 2 SH3 domain binds to N-cadherin cytodomain via two PxxP motifs.

We next examined the expression of the mutant form of N-cadherin phenocopies pacsin 2 KD. Exogenously expressed GFP-N-cadherin weakly accumulated at the cell-cell contact sites, but the formation of interdigitated F-actin structures was rarely observed (Fig. 8, D, N-cadWtGFP). In contrast, exogenously expressed N-cadherin mutants with combined PA mutations (P818/821A + P847/850/851A) strongly accumulated to the cell-cell contact sites frequently inducing interdigitated F-actin structures (Fig. 8, D, N-cadPAGFP). Although it is still preliminary, quantitative analyses showed more than 80 % of cells expressing PxxP mutant N-cadherin induced cell-cell contacts, while only around 30% of wild-type N-cadherin expressing cells induced cell-cell contacts. These results strongly suggest that pacsin 2 regulates N-cadherin internalization required for collective cell migration of T24 cells.

Reviewer #2

Major comments

1. *This is potentially interesting finding, in particular the role of Pacsin controlling endocytosis of N-cadherin. However, in general the results are still preliminary in my opinion and this study would benefit from CTRL experiments and quantification of some of the results. For example, in Figure 1, the authros conclude that Pacsin 2 does not localize to invadopodia based on the images shown. First, a single cell is shown in each panel with no quantification. Second, not all staining positive for cortactin or dynamin represents an invadopodia structure. It is not unusual to see punctate staining in cells that is cortactin and dynamin positive and may represent vesicles. Third, not all cells form invadopodia at the same time, so showing one cell should not be sufficient evidence to draw conclusions. A definitive proof of invadopodia formation would be to use a matrix degradation assay. Finally, it would have been nice to use*

© 2023. Published by The Company of Biologists under the terms of the Creative Commons Attribution License (https://creativecommons.org/licenses/by/4.0/).

the Pacsin 2 siRNA cells to validate the specificity of the antibodies.

Following the comments of Reviewer #2, the localization of pacsin 2 was analyzed in combination with a matrix degradation assay using FITC-gelatin. In this analysis, matrix degradation occurs in perinuclear regions where typical actin dots are formed (Fig. 1, D, upper panel). In contrast, pacsin 2 did not localize in the perinuclear regions but it was observed at the cell periphery (Fig.1, D, lower panel). Quantification analyses of more than 315 cells with invadopodia (N=3) showed that none of pacsin 2 localized to the invadopodia. The specificity of the antibodies was also validated in pacsin 2 siRNA cells (new Fig. S5).

2. *In Figure 3, only 10 cells/exp are quantified. Is this a sufficient number fir statistical significance? Also, how were these cells selected? More details in the Methods sections are needed*

   For the statistical analyses of the live cell imaging, 10 cells in the leading edge were randomly selected from/exp and the data from 4 independent experiments (altogether 40 cells) were used to obtain quantitative results for velocity and directionality. Detailed information for Figure 3 is described in the methods section of the revised manuscript.

3. *In addition, there is some overlap with key Pacsin2 articles in the literature that were not cited in the manuscripts, in particular the publications that show Pacsin 2 is involved in collective cell migration (de Kreuk 2011; DOI: 10.1242/jcs.080630. Other key references not cited: Malinova 2021 (DOI: 10.1038/s41467-021-22873-y), de Kreuk 2012 (DOI: 10.1074/jbc.M112.391078) and Meng 2011 (DOI: 10.4161/cc.10.1.14243)*

   We apologize that some of the key references were not cited in the original manuscript. Although the paper by de Kreuk et al (DOI: 10.1242/jcs.080630) was cited in the original manuscript, the other key references were also cited in the revised manuscript.

4. *In my opinion, Figure 5 shows the most interesting results. However, I have several question/comments regarding these results: a) most figures show one or two cells positive for N-cadherin at cell junctions, but there is no quantification to show what is the difference between CTRL and KD cell lines. The text states that 34% of CTRL cells form cell-cell contacts. Are these contacts mediated by N-cadherin? A picture of cell-cell contacts found in CTRL cell would be informative.*

   We thank Reviewer #2 for the comments. Control RNAi cells did not show N-cadherin accumulation at the cell contact sites even in a densely plated condition (new Fig. S5, A and B, siCtrl). In contrast, densely plated pacsin 2 RNAi cells exhibited strong N-cadherin accumulation at the cell-cell contact sites (new Fig. S5, A and B, siPacsin2 #1, 2 and 3). These data suggest that cell-cell contacts formed by control RNAi cells are not mediated by N-cadherin.

5. *The other question not answered/quantified is whether dynamin KD cells also increase the number of cells that form cell-cell junctions.*

   Dynamin 2 KD cells also increased the number of cells that form cell-cell junctions as shown in the original manuscript (new Fig. S1 and Fig. S6).

6. *The images in Figure 6 are quite striking. However, there is not really a discussion of what does this mean in terms of inhibiting endocytosis and keeping N-cadherin at the plasma membrane. Since the connection between cells look so different, the same question asked above is relevant, i.e. are the cell-cell junctions in CTRL cells also mediated by N-cadherin.*

   Our data support that cell-cell contacts formed by control RNAi cells are not mediated by N-cadherin, whereas they are mainly mediated by N-cadherin in pacsin 2 RNAi cells (Fig. 5 and new Fig. S5). In pacsin 2 RNAi cells, the surface level of N-cadherin is increased because of inhibited internalization of N-cadherin (Fig. 7), which induces cell- cell contact formation. It is also possible that, in pacsin 2 RNAi cells, an excess amount of membrane components are accumulated because of inhibited endocytosis, which may support the formation of interdigitated membranous structures at the cell-cell contact sites. These possible mechanisms will be described in the discussion section.

© 2023. Published by The Company of Biologists under the terms of the Creative Commons Attribution License (https://creativecommons.org/licenses/by/4.0/).

7. *The interaction between N-cadherin and Pacsin 2 is only shown in vitro and using fragments of each protein. The manuscript would be strengthened inf the interaction is confirmed with endogenous proteins.*

We agree with the comments of Reviewer #2 that the manuscript would be strengthened if the interaction is confirmed with endogenous proteins. However, the experiment may be challenging because endogenous interaction between N-cadherin and pacsin 2 could occur temporarily. Instead, to make the *in vitro* data more convincing, candidate interaction sites in N-cadherin cytodomain (PxxP motifs) were mutated and their interaction with pacsin 2 SH3 domain was examined (new Fig. 8, C). In the *in vitro* binding assay, wild type form of GFP-tagged N-cadherin cytodomain was efficiently pulled down with GST-tagged pacsin 2 SH3 domain (new Fig. 8, C, Wt). In contrast, the interaction of PxxP mutants of GFP-tagged N-cadherin cytodomain with pacsin 2 SH3 domain was suppressed (new Fig. 8, C, P818/821A, P847/850A, P847/850/851A, P818/821A+P847/850A or P818/821A+P847/850/851A). This result suggests that SH3 domain of pacsin 2 binds to both PxxP motifs in N-cadherin cytodomain.

Furthermore, exogenously expressed GFP-tagged mutant N-cadherin (P818/821A + P847/850/851A) in T24 cells localized to the cell-cell contact sites inducing interdigitating F-actin (new Fig. 8, D, N-cadPAGFP) similarly as the endogenous N-cadherin in pacsin 2 KD cells (Fig. 5). In contrast, GFP-tagged wild type N-cadherin weakly accumulated to cell-cell contact sites and interdigitated F-actin was rarely formed (new Fig. 8, D, N- cadWtGFP). Consistently, preliminary quantitative analysis showed that around 30% of T24 cells expressing wild-type GFP-tagged N-cadherin induces cell-cell contacts, while more than 80% of cells forms cell-cell contacts when mutant N-cadherin was expressed. These results suggest that pacsin 2 interacts with cytodomain of N-cadherin to regulate its internalization in the collective cell migration of T24 cells.

8. *The finding regarding FA numbers is also interested but somehow disconnected to the rest of the story. How does the inhibition of N-cadherin endocytosis regulate FA turnover? How does it affect the directionality of migration and why, as it would be predicted, it does not slow down migration? Also, the Pacsin 2 KD phenotype shows more FA at the center of the cells (at least in the images shown) whereas for dynamin KD they seem to localize at the periphery of the cells. Based on those pictures alone, the phenotypes appear to be different. To be able to confirm that, quantification of the position of the FA regarding the edge of the cell (distance) should be quantified (or alternative % of FA at the periphery vs center of the cell).*

We confirmed that FA numbers are also increased in single-cell conditions both in pacsin 2 RNAi and DNM2 RNAi cells (new Fig.S9), suggesting that FA turnover is directly regulated by pacsin 2 but not by inhibition of N-cadherin turnover.

We apologize for showing misleading images about FA distribution in pacsin 2 RNAi and dynamin 2 RNAi cells. We reconfirmed that the spatial distribution of FAs showed no clear differences in pacsin 2 RNAi cells and dynamin 2 RNAi cells (Fig. 9, Fig S8 and Fig. S9).

We thank Referee #2 for the important question about the effect of increased FA numbers on directionality and velocity. In the collective cell migration, polarized FA distribution along the direction of migration together with cadherin-mediated cell-cell contacts could contribute to determining directionality. Regarding the velocity, as the reviewer mentioned, it could be predicted that an increased FA number (decreased FA turnover) may slow down migration for singly migrating cells. However, in the case of collective cell migration, the cellular interactions not only with the surrounding extracellular matrix but also with the neighbouring cells are required to allow a group of cells to migrate in a coordinated manner. Although precise mechanisms of collective cell migration are still under debate, the mechanisms of finely balanced cell-cell and cell- extracellular adhesion are discussed in the manuscript.

9. *Some more details in the methods would be useful, e.g. number of cells plated, quantification of tracks during wound healing, how cells were selected, how minimum N- number was determined, what type of statistical analysis was performed.*

© 2023. Published by The Company of Biologists under the terms of the Creative Commons Attribution License (https://creativecommons.org/licenses/by/4.0/).

The methods section was revised so that the detailed methods can be understood.

Minor comments

1. *I suggest merging Fig 1 with Fig S1*

   Fig. 1 and Fig. S1 were merged.

2. *In Fig 4 it looks like some of the KD cells have a larger area. Since Pacsin 2 has been associated with spreading, it would be important to measure whether in these cells spreading is also affected.*

   Pacsin 2 has been implicated in the regulation of cell spreading and migration by associating with Rac 1 (*de Kreuk 2011; DOI: 10.1242/jcs.080630*). This paper was cited in the original manuscript but it is more clearly described in the introduction of the revised manuscript.

Reviewer #3

1. *In follow-up of that question, in endothelial cells Pacsin2 has been shown to control the internalization of VE-cadherin, and that this trafficking function of Pacsin2 (via the recruitment of the recycling mediators EHD4 and MICAL-L1) is in fact needed to drive directed collective cell migration in scratch assays (Malinova et al, 2021; PMID: 33972531). The authors are asked to discuss how those findings might relate to the finding that depletion of Pacsin2 in fact promotes collective cell migration in T25 cells and thus has the opposite effect on N-cadherin trafficking. What mechanism might underlie this?*

   We thank Reviewer #3 for raising this important issue. We read the excellent studies from Huveneers Lab (Malinova et al, 2021: PMID: 33972531; Dorland et al, 2016: PMID: 27417273) with the greatest interest since pacsin 2 (via the recruitment of the recycling mediators EHD4 and MICAL-L1) is needed to drive directed collective cell migration. The opposite effect of pacsin 2 KD in collective cell migration in our study and theirs may be due to differently regulated internalization of the two cadherin isoforms: internalization of VE-cadherin in HUVEC cells is inhibited by pacsin 2 (Fig. 9 in Dorland et al, 2016), while N-cadherin internalization in T24 cells is positively regulated by pacsin 2 (Fig.7 in our study). Interestingly, cytoplasmic regions of N- and VE-cadherins are divergent except for p120-catenin binding site, and two PxxP motifs in N-cadherin are not conserved in VE-cadherin (Figure below). Instead, VE-cadherin contains another PxxP motif which is not conserved in N-cadherin proximal to p120-catenin binding site. Although precise mechanisms remain to be elucidated, we speculate that pacsin 2 may be more accessible to cytodomain of N-cadherin compared to that of VE-cadherin, because (1) two PxxP motifs in N-cadherin cytodomain locate distal to p120-catenin binding site, (2) the PxxP motif in VE-cadherin cytodomain locates proximal to the p120- catenin binding site. Consistently, pacsin 2 recruitment occurs in parallel with the dissociation of p120-catenin from VE-cadherin (Fig. 3, Malinova et al, 2021), suggesting a possible interference by p120-catenin complex for pacsin 2 binding to VE-cadherin.

```
N-Cadherin_cyto      MKRRDKERQAKQLLIDPEDDVRDNILKYDEEGGGEEDQD-YDLSQLQQPDTVEPDAIKPV
VE-Cadherin_cyto     ---RRRLRKQARAHGKSVPEIHEQLVTYDEEGGGEMDTTSYDVSVLNSVRRGGAKPPRPA
                        * : *:  :    ..  ::::::::.******** *    **:* *:.      ... :*.

N-Cadherin_cyto      GIRRMDERPIHAEPQYPVRSAAPHPGDIGDFINEGLKAADNDPTAPPYDSLLVFDYEGSG
VE-Cadherin_cyto     LDARPSLYAQVQKPPRHAPGAHGGPGEMAAMIEVKKDEADHDGDGPPYDTLHIYGYEGSE
                        *  .  .    :*    .  .*    **::. :*:    . **:*  .****:* ::.****

N-Cadherin_cyto      STAGSLSSLNSSSSGGEQDYDYLNDWGPRFKKLADMYGGGDD-----
VE-Cadherin_cyto     SIAESLSSLGTDSSDSDVDYDFLNDWGPRFKMLAELYGSDPREELLY
                     * * *****.:.**..: ***:********* **::**..
```

Amino acid sequence alignment of cytodomains in N- and VE-cadherins.
P120-catenin binding sites (underlined) and PxxP motifs (bold and italicized in red) in human N-cadherin and VE- cadherin are shown.

© 2023. Published by The Company of Biologists under the terms of the Creative Commons Attribution License (https://creativecommons.org/licenses/by/4.0/).

2. *Please correct adherence junctions to adherens junctions.*
   We apologize for the typo. All the “Adherence junctions” were corrected to “adherens junctions".

3. *Where does pacsin2 localize in confluent T25 cell cultures?*
   In control RNAi cells at high density, pacsin 2 does not localize to the cell-cell contact sites, but it still localizes to the cell periphery, especially at the edge of cell clusters (new Fig.S5, B). We will also reconfirm the localization of endogenous pacsin 2 in untreated T24 cells at high density in the full revision of the manuscript.

4. *The relocation/trafficking of N-cadherin might depend on the presence of other classical cadherins. It would be important to know how the depletion of pacsin2 affects the surface levels of all expressed classical cadherins.*
   Expression profiles of three classical cadherins (E-, N, and P-cadherins) and one unconventional cadherin (VE-cadherin) in T24 cells were examined, and N-cadherin appeared to be the sole cadherin isoforms expressed in T24 cells (Fig. S2). Furthermore, we show that 1) pacsin 2 interacts with PxxP motifs in N-cadherin cytoplasmic domain, and 2) overexpression of PA mutants of N-cadherin phenocopied pacsin 2 KD cells (Fig. 8). These data suggest that effect of pacsin 2 depletion on the relocation/trafficking of N-cadherin is rather direct but it is unlikely to be dependent on the presence of other cadherin subtypes.

5. *Figure 5; more confluent cultured controls cells should be shown to be able to compare AJ formation in siControl vs. siPacsin2. This also accounts for the other figures in which cell clustering was investigated.*
   Densely plated control and pacsin2 RNAi cells with double-staining for N-cadherin and Pacsin2 were examined to assess the effects of pacsin 2 KD on N-cadherin localization at junctions (the new Fig. S5). In densely plated pacsin 2 RNAi cells, N-cadherin was accumulated to the cell-cell contact sites in a similar manner as sparsely plated pacsin 2 RNAi cells (new Fig. S5, A and B, siPacsin2 #1, 2 and 3). In contrast, N-cadherin accumulation at the cell contact sites was not observed in control RNAi cells even in a densely plated condition (new Fig. S5, A and B, siCtrl). These data suggest that N-cadherin at cell-cell contacts is due to pacsin 2 expression level rather than initial differences in plating density.

6. *Is the effect of focal adhesion formation specific for pacsin2, or might it depend on the level of junction formation? What happens to FAs in single-cell conditions?*
   We confirmed that pacsin KD cells demonstrated an increased number of FAs in single-cell conditions (new Fig. S9), suggesting that the effect of focal adhesion formation is specific for pacsin 2 but not dependent on the level of junction formation.

4 Description of analyses that authors prefer not to carry out
None

Original submission

First decision letter

MS ID#: JOCES/2022/260827

MS TITLE: Pacsin 2-dependent N-cadherin internalization regulates the migration behaviour of malignant cancer cells

AUTHORS: Haymar Wint, Jianzhen Li, Tadashi Abe, Hiroshi Yamada, Yasutomo Nasu, Masami Watanabe, Kohji Takei, and Tetsuya Takeda
ARTICLE TYPE: Research Article

© 2023. Published by The Company of Biologists under the terms of the Creative Commons Attribution License (https://creativecommons.org/licenses/by/4.0/).

We have now reached a decision on the above manuscript.

To see the reviewers' reports and a copy of this decision letter, please go to: https://submit-jcs.biologists.org and click on the 'Manuscripts with Decisions' queue in the Author Area. (Corresponding author only has access to reviews.)

As you will see, the reviewers raise a number of substantial criticisms that prevent me from accepting the paper at this stage. They suggest, however, that a fully revised version that addresses their additional concerns might prove acceptable. If you think that you can deal satisfactorily with the criticisms on revision, I would be pleased to see a revised manuscript.

Please ensure that you clearly highlight all changes made in the revised manuscript. Please avoid using 'Tracked changes' in Word files as these are lost in PDF conversion.

I should be grateful if you would also provide a point-by-point response detailing how you have dealt with the points raised by the reviewers in the 'Response to Reviewers' box. Please attend to all of the reviewers' comments. If you do not agree with any of their criticisms or suggestions please explain clearly why this is so.

Reviewer 1

Advance summary and potential significance to field

As Reviewer#1 from Reviewer Commons, I find the research question solid.

The overall findings expand what is known about Pacsin2 loss-of-function phenotypes relevant to cell migration. Use of the single T24 bladder cancer cell line (E-cad-; N-cad+) may narrow the overall generality of the model. But upon revision, it is now clear the ability of Pacsin2 to negatively regulate N-cadherin accumulation at cell-cell contacts is likely due to distinct sequences in the N-cadherin tail (in comparison to E-cadherin or VE-cadherin). Thus, it appears the mechanism put forth here operates within the context of a cancer cell line that only expresses N-cadherin.

Comments for the author

I am mostly satisfied with this revision, particularly efforts at data transparency with low magnification immunofluorescence views in Supplement, as well as mapping the Pacsin2 interaction motif on N-cadherin, mutating that motif and suggesting evidence of enhanced recruitment to/stabilization of N-cad-based cell-cell adhesions (new Fig. 8). However the data in panel D of Figure 8 really need to be quantified. The difference in junction localization looks very subtle- and so the authors should endeavor to quantify this effect-- even if modest.
Ideally, the authors would find a way, perhaps with a barrier/chamber to co-culture the WT and mutant N-cadherin so that the accumulation could be quantified with the same exposure/image.

Reviewer 2

Advance summary and potential significance to field

The findngs by Wint et al uncover a role for pacsin2 in the formation of N-cadherin based junctions in T25 bladder cancer cells. This way pacsin2 can control the collective migration, and thus potentially cancer cell invastion, of such cell types.

Comments for the author

The authors have already addressed the majority of the points that were previously raised by the reviewers at Review Commons. This strengthened the current version of the manuscript. Moreover, the authors indicate that they are further addressing some questions in preparation of the full revision.

© 2023. Published by The Company of Biologists under the terms of the Creative Commons Attribution License (https://creativecommons.org/licenses/by/4.0/).

In the cover letter the authors indicate that they will complete the paper by showing that the antibodies for pacsin3 and VE-cadherin are working. This would indeed be needed to draw conclusions on their presence (or absence) in the lysates of T25 cells.

The authors will also address the role of pacsin2 in HeLa or H1299, to assess how general the role of pacsin2 mediated control over N-cadherin and/or E-cadherin is. This would be very informative and will define how general the role of pacsin2 is, or whether the pacsin2 and N-cadherin interaction is specific.

I have no further major comments. Only some small points:

DAPI seems missing from the merged image in Figure 8D
Figure S9: paxillin is misspelled in the grayscale images.

Reviewer 3

Advance summary and potential significance to field

This is potentially interesting finding, in particular the role of Pacsin 2 controlling endocytosis of N-cadherin. The authors show that in the absence of Pacsin 2, endocytosis of N-cadherin is inhibited, with cell surface N-cadherin functioning to reinforce cell-cell junctions and enhance collective cell migration and directionality.

Comments for the author

The authors have addressed most of my initial comments from Review Commons, as well of those of other reviewers. There are still a few issues that, if addressed, would strengthen the manuscript.

Major comments
The connection between pacsin 2 binding to N-cadherin and endocytosis is not conclusively demonstrated. First, the interaction is only demonstrated with isolated domains, which given the concentrations used in pulldown domains may or may not translate to an in vivo interaction. I would recommend that , at least the interaction between FL proteins is tested, preferably endogenous. Second, expression of the PA mutant of N-cadherin seem to support this hypothesis. However, it may help to show that, compared to the wild type, the endocytosis rate of the PA mutant is reduced.
In the same figure 3B, it looks like siCTRL cells travel more total distance which may suggest they are not impaired in migration just directionality. The texts seems to indicate that pacsin KD enhances migration, but that is not completely accurate, as the distance and velocity seem to be reduced with pacsin KD. Only directionality seems to be enhanced.
I disagree with the statement in p6 that pacsin 2 colocalizes with dynamin at the periphery and not with cortactin. I would argue that the pictures look very similar. Again, here colocalization analysis or quantification (histogram profiles or something like that) would strengthen the authors conclusions.
-I still believe that the finding regarding FA numbers is very interesting but somehow disconnected to the rest of the story. How does the inhibition of N-cadherin endocytosis regulate FA turnover? How does it affect the directionality of migration and why, as it would be predicted, it does not slow down migration? Also, the Pacsin 2 KD phenotype shows more FA at the center of the cells (at least in the images shown) whereas for dynamin KD they seem to localize at the periphery of the cells. Based on those pictures alone, the phenotypes appear to be different. To be able to confirm that, quantification of the position of the FA regarding the edge of the cell (distance) should be quantified (or alternative % of FA at the periphery vs center of the cell).

Minor comments
In Figure 3B, the labels for the x and y axes are missing.
In Figure 8, quantification of the difference between WT and N-cadherin at the junctions is recommended.
In Figure S5A and B, the panels for Pacsin2 KD #3 show cells that look significantly larger than the other conditions. The figure seems to indicate that the scale is the same for all pictures. Can you

© 2023. Published by The Company of Biologists under the terms of the Creative Commons Attribution License (https://creativecommons.org/licenses/by/4.0/).

verify whether the cells are larger, and if so how to explain it, or whether the siPacsin2 #3 is at a different scale? Since Pacsin 2 has been associated with spreading, it would be important to measure whether in these cells spreading is also affected.
-In Fig1, without a positive CTRL, the specificity of Pacsin 3 antibody cannot be confirmed. Similarly, in Fig S2 for VE-cadherin
-In Fig 9, the pictures shown suggest a different phenotype between si #1 vs. #2 and #3.

First revision

Author response to reviewers' comments

Reviewer 1
Reviewer 1 Advance Summary and Potential Significance to Field:
As Reviewer#1 from Reviewer Commons, I find the research question solid.

The overall findings expand what is known about Pacsin2 loss-of-function phenotypes relevant to cell migration. Use of the single T24 bladder cancer cell line (E-cad-; N-cad+) may narrow the overall generality of the model. But upon revision, it is now clear the ability of Pacsin2 to negatively regulate N- cadherin accumulation at cell-cell contacts is likely due to distinct sequences in the N-cadherin tail (in comparison to E-cadherin or VE-cadherin). Thus, it appears the mechanism put forth here operates within the context of a cancer cell line that only expresses N-cadherin.

Reviewer 1 Comments for the Author:
I am mostly satisfied with this revision, particularly efforts at data transparency with low magnification immunofluorescence views in Supplement, as well as mapping the Pacsin2 interaction motif on N- cadherin, mutating that motif and suggesting evidence of enhanced recruitment to/stabilization of N-cad- based cell-cell adhesions (new Fig. 8). However the data in panel D of Figure 8 really need to be quantified. The difference in junction localization looks very subtle- and so the authors should endeavor to quantify this effect-- even if modest. Ideally, the authors would find a way, perhaps with a barrier/chamber to co-culture the WT and mutant N-cadherin so that the accumulation could be quantified with the same exposure/image.

We thank Reviewer 1 for the positive and helpful comments. As requested, we quantified the junctional localization of exogenously expressed GFP-tagged N-cadherin in T24 cells. As the reviewer pointed out, the difference in immunofluorescence images looked very subtle, but the quantification analysis showed that the intensity of accumulated GFP-tagged PA mutant (P818/821/847/850/851A) at the cell-cell junctions was higher than that of wild type N-cadherin with a statistical significance (new Fig. 6F and G). Consistently, T24 cells expressing PA mutant N-cadherin form more cell-cell contacts (85%) compared to T24 cells expressing wild-type N-cadherin (38%) (new Fig. 6H). These results suggest that internalization of N-cadherin PA mutant from the cell surface was downregulated due to its defective association with pacsin 2. Indeed, the biotinylation and endocytosis assay showed that internalization of N-cadherin PA mutant was attenuated compared to that of wild-type N-cadherin (new Fig. 7). Taken these results together, we concluded that pacsin 2-mediated internalization of N-cadherin negatively regulate cell-cell contact formation and/or maintenance of T24 cells.

Reviewer 2
Reviewer 2 Advance Summary and Potential Significance to Field:
The findngs by Wint et al uncover a role for pacsin2 in the formation of N-cadherin based junctions in T25 bladder cancer cells. This way pacsin2 can control the collective migration, and thus potentially cancer cell invastion, of such cell types.

Reviewer 2 Comments for the Author:
The authors have already addressed the majority of the points that were previously raised by the reviewers at Review Commons. This strengthened the current version of the manuscript. Moreover, the authors indicate that they are further addressing some questions in preparation of

© 2023. Published by The Company of Biologists under the terms of the Creative Commons Attribution License (https://creativecommons.org/licenses/by/4.0/).

the full revision. In the cover letter the authors indicate that they will complete the paper by showing that the antibodies for pacsin3 and VE-cadherin are working. This would indeed be needed to draw conclusions on their presence (or absence) in the lysates of T25 cells.

We thank Reviewer 2 for helpful comments. To reconfirm the expression of pacsin 3 in T24 cells, we used an alternative anti-pacsin 3 antibody, mouse monoclonal anti-PACSIN3 (C-3) (sc-166923, Santa Cruz), in the immunoblot analysis. As a result, we showed that pacsin 3 in fact expressed in T24 cells (new Fig. 1A, Pacsin 3). However, pacsin 3 dispersedly localized in the cytoplasm of T24 cells (new Fig. 1B, Pacsin 3) in a similar manner as pacsin 1 (new Fig. 1B, Pacsin 1), whereas pacsin 2 specifically localized at the cell periphery (new Fig. 1B, Pacsin 2). Thus, we decided to maintain the overall stories of the manuscript that focuses on the pacsin 2 function in T24 cells.

To reconfirm the expression of VE-cadherin in the immunoblot analysis, a positive control (total cell extract of HUVEC cells) was included together with samples from T24 cells. As a result, we could confirm expression of VE-cadherin in HUVEC cells, but not in total cell extract of either RT4 or T24 cells (new Fig. S2, VE-cadherin). We also reconfirmed P-cadherin expression using a sensitive detection reagent (SuperSignal West Pico PLUS Chemiluminescent Substrate, 34580, ThermoFisher Scientific).

Consistent with a previous study (Mialhe et al., J. Urol. 2000), P-cadherin was expressed in RT4 cells, but not in T24 cells (new Fig. S2, P-cadherin). Thus, we concluded that N-cadherin, but not E-, P- or VE- cadherin, was expressed in T24 cells.

The authors will also address the role of pacsin2 in HeLa or H1299, to assess how general the role of pacsin2 mediated control over N-cadherin and/or E-cadherin is. This would be very informative and will define how general the role of pacsin2 is, or whether the pacsin2 and N-cadherin interaction is specific.

We examined the role of pacsin 2 in a human non-small lung cancer cell line H1299. Firstly, we confirmed that H1299 cells showed similar expression profiles of cadherins (N-cad+; E-cad-; P-cad-; VE- cad-) as is the case for T24 cells (new Fig. S5, A). Secondly, we confirmed that pacsin 2 also expressed in H1299 cells (new Fig. S5, B) and colocalized with N-cadherin at the cell periphery (new Fig. S5, C).

Finally, pacsin 2 KD in H1299 induced cell-cell contacts enriched with N-cadherin (new Fig. S6). Taking these data together, we concluded that the ability of pacsin 2 to negatively regulate N-cadherin accumulation at cell-cell contacts seems to be conserved at least within the context of a cancer cell line that expresses only N-cadherin.

I have no further major comments. Only some small points:
DAPI seems missing from the merged image in Figure 8D

We apologize for not showing DAPI staining clearly in Figure 8D. The signal intensity of DAPI was enhanced so that it is visible in the merged images (new Fig. 6F).

Figure S9: paxillin is misspelled in the grayscale images.

We apologize for the typo. All the misspelling of Paxillin in greyscale was amended (new Fig. S8).

Reviewer 3

Reviewer 3 Advance Summary and Potential Significance to Field:
This is potentially interesting finding, in particular the role of Pacsin 2 controlling endocytosis of N- cadherin. The authors show that in the absence of Pacsin 2, endocytosis of N-cadherin is inhibited, with cell surface N-cadherin functioning to reinforce cell-cell junctions and enhance collective cell migration and directionality.

Reviewer 3 Comments for the Author:
The authors have addressed most of my initial comments from Review Commons, as well of those of other reviewers. There are still a few issues that, if addressed, would strengthen the manuscript.

© 2023. Published by The Company of Biologists under the terms of the Creative Commons Attribution License (https://creativecommons.org/licenses/by/4.0/).

Major comments
The connection between pacsin 2 binding to N-cadherin and endocytosis is not conclusively demonstrated. First, the interaction is only demonstrated with isolated domains, which given the concentrations used in pulldown domains may or may not translate to an in vivo interaction. I would recommend that, at least the interaction between FL proteins is tested, preferably endogenous. Second, expression of the PA mutant of N-cadherin seem to support this hypothesis. However, it may help to show that, compared to the wild type, the endocytosis rate of the PA mutant is reduced.

We thank Reviewer 3 for the helpful comments. Firstly, using the GST pull-down assay, we demonstrated endogenous N-cadherin in T24 cells bound to the pacsin 2 SH3 domain (new Fig. 6C). We also confirmed that GFP-tagged full-length wild type N-cadherin, but not PA mutant (P818/821/847/850/851A), bound to pacsin 2 SH3 domain in the same manner as isolated N-cadherin cytoplasmic domain (new Fig. 6E). Secondly, using the biotinylation and endocytosis assay, we showed that the endocytosis level of the PA mutant N-cadherin is reduced compared to that of wild-type N- cadherin (new Fig. 7). Based on these data, we concluded that pacsin 2 SH3 domain indeed interacts with cytoplasmic regions of N-cadherin to regulate its internalization from the cell surface.

In the same figure 3B, it looks like siCTRL cells travel more total distance which may suggest they are not impaired in migration just directionality. The texts seems to indicate that pacsin KD enhances migration, but that is not completely accurate, as the distance and velocity seem to be reduced with pacsin KD. Only directionality seems to be enhanced.

We agree with the perspectives of Reviewer 3 on the migration behaviour of pacsin 2 KD cells. To describe the phenotype more precisely, the subtitle for Fig. 3 (new Fig. 2) (P.6) was changed from "*Pacsin 2 depletion enhances the migration of T24 cells*" to "*Pacsin 2 depletion induces directional migration of T24 cells*".

I disagree with the statement in p6 that pacsin 2 colocalizes with dynamin at the periphery and not with cortactin. I would argue that the pictures look very similar. Again, here colocalization analysis or quantification (histogram profiles or something like that) would strengthen the authors conclusions.

We apologize for the confusing statement in P6. We were intended to state that pacsin 2 co-localizes with both dynamin 2 and cortactin at the cell periphery, but the colocalization was not observed at perinuclear invadopodia in T24 cells. To make our statement clearer, we included enlarged images of their localization in perinuclear invadopodia and cell periphery (new Fig. 1C).

I still believe that the finding regarding FA numbers is very interesting but somehow disconnected to the rest of the story. How does the inhibition of N-cadherin endocytosis regulate FA turnover? How does it affect the directionality of migration and why, as it would be predicted, it does not slow down migration?

We thank Reviewer 3 for the comments, but we have already addressed this point in the "Revision plan" of Review Commons. We confirmed that FA numbers are increased in single-cell conditions in pacsin 2 RNAi (new Fig. S8A and B) and dynamin 2 RNAi (new Fig. S8C and D), suggesting that FA turnover is regulated by pacsin 2 and dynamin 2 more directly rather than indirectly regulated as a result of inhibited N-cadherin endocytosis. Regarding the effect of increased FA numbers on the directionality and velocity of cell migration, it is believed that polarized FA distribution along the direction of migration together with cadherin-mediated cell-cell contacts could cooperatively contribute to determining the directionality of the collectively migrating cells. As the reviewer mentioned, an increased FA number (decreased FA turnover) could slow down migration for singly migrating cells, and it could be the case since we observed a slight decrease of the velocity in pacsin 2 KD cells (new Fig. 2F). However, in the case of the collectively migrating cells, the cellular interactions not only with the surrounding extracellular matrix but also with the neighbouring cells are required to allow a group of cells to migrate in a coordinated manner. Although precise mechanisms of collective cell migration are still largely unknown, we believe that the mechanisms of finely balanced cell-cell and cell-extracellular adhesion are required for collective cell migration.

© 2023. Published by The Company of Biologists under the terms of the Creative Commons Attribution License (https://creativecommons.org/licenses/by/4.0/).

Also, the Pacsin 2 KD phenotype shows more FA at the center of the cells (at least in the images shown) whereas for dynamin KD they seem to localize at the periphery of the cells. Based on those pictures alone, the phenotypes appear to be different. To be able to confirm that, quantification of the position of the FA regarding the edge of the cell (distance) should be quantified (or alternative % of FA at the periphery vs center of the cell).

Having collaboration with an expert in image analysis (Dr Takumi Higaki, Kumamoto University), we have quantified the spatial distribution of FAs in pacsin 2 KD and dynamin 2 KD cells. As a result, FAs induced by depletion of pacsin 2 and dynamin 2 tend to localize in peripheral regions in T24 cells (new Fig. 8C and Fig. S7C). The effect of pacsin 2 KD on FA distribution showed statistical significance for siPacsin 2 #2 and #3, but not with siPacsin 2 #1, probably due to different level of pacsin 2 depletion among these pacsin 2 siRNAs.

Minor comments
In Figure 3B, the labels for the x and y axes are missing.
Labels for the x and y axes in Fig. 3B (new Fig. 2E) were added.

In Figure 8, quantification of the difference between WT and N-cadherin at the junctions is recommended.
Quantification analysis showed that the intensity of GFP-tagged PA mutant (P818/821/847/850/851A) at the cell junctions was higher than that of wild-type N-cadherin with a statistical significance (new Fig. 6G).

In Figure S5A and B, the panels for Pacsin2 KD #3 show cells that look significantly larger than the other conditions. The figure seems to indicate that the scale is the same for all pictures. Can you verify whether the cells are larger, and if so how to explain it, or whether the siPacsin2 #3 is at a different scale? Since Pacsin 2 has been associated with spreading, it would be important to measure whether in these cells spreading is also affected.
As Reviewer 3 pointed out (and it is also referred to in our manuscript), pacsin 2 has been implicated in cell spreading and migration by negatively regulating the Rac1-mediated signalling pathway (de Kreuk et al., *Journal of Cell Science* 2011, DOI: *10.1242/jcs.080630). Thus, it was not surprising that cell spreading was also promoted by pacsin 2 KD in T24 cells in the same manner as HeLa cells used in the study by de Kreuk et al. Indeed, in a low cell density condition, pacsin 2 KD cells looked larger than control RNAi cells (Fig. S3, Fig. 8 and Fig. S8A). Indeed, quantitative analysis showed that average size of pacsin 2 KD cells was larger with statistical significance irrespective of siRNAs used for the pacsin 2 RNAi (right panel). We were also aware that pacsin2 KD #3 cells looked larger in a high cell density condition (Figure S4A and B). We thought that effect* of the cell contact inhibition on cell size control may not be negligible especially in a high cell density condition, although it is generally downregulated in cancer cells. Future studies may reveal the coordinated functions of pacsin 2 and the cell contact inhibition in the cell size control, but we are not intend to go into the mechanistic insights since it is beyond the scope of this study.

NOTE: We have removed unpublished data that had been provided for the referees in confidence.

In Fig1, without a positive CTRL, the specificity of Pacsin 3 antibody cannot be confirmed. Similarly, in Fig S2 for VE-cadherin

We thank Reviewer 3 for the comments. To reconfirm the expression of pacsin 3 in T24 cells, we used an alternative anti-pacsin 3 antibody, mouse monoclonal anti-PACSIN3 (C-3) (sc-166923, Santa Cruz), in the immunoblot analysis. As a result, we showed that pacsin 3 in fact expressed in T24 cells (new Fig. 1A). However, pacsin 3 localized dispersedly in the cytoplasm (new Fig. 1B, Pacsin 3) in a similar manner as pacsin 1 (new Fig. 1B, Pacsin 1), while pacsin 2 showed specific localization at the cell periphery (new Fig. 1B, Pacsin 2), Therefore, we maintained overall story of the manuscript focusing on pacsin 2 function in T24 cells.

Regarding VE-cadherin, we included a positive control (total cell extract of HUVEC cells) together with samples from T24 cells in the immunoblot analysis. As a result, VE-cadherin expression in HUVEC cells was confirmed, whereas it was undetectable in total cell extract of either RT4 or T24 cells (new Fig. S2A, VE-cadherin). Therefore, we concluded that VE-cadherin is not expressed in both RT4 and T24 cells at least at a detectable level by immunoblot analyses.

© 2023. Published by The Company of Biologists under the terms of the Creative Commons Attribution License (https://creativecommons.org/licenses/by/4.0/).

In Fig 9, the pictures shown suggest a different phenotype between si #1 vs. #2 and #3.

We have quantified the spatial distribution of FA in pacsin 2 KD. As a result, pacsin 2 KD cells tend to form FAs in peripheral regions of T24 cells (new Fig. 8C). The effect of pacsin 2 KD on FA distribution showed statistical significance for siPacsin 2 #2 and #3, but not for siPacsin 2 #1, probably due to different depletion levels among these siRNAs used for the pacsin 2 RNAi.

Other changes

Author list

Dr Takumi Higaki was added as a new co-author. All of the authors (including the new author) are happy with the new author list and sent their statement "I confirm that I am happy with the new author list of the manuscript JCS260827" to the editorial office of Journal of Cell Science via e-mail.

Removal of figure

"Fig. S7 Expression level of N-cadherin in T24 cells is not affected by depletion of either pacsin 2 or dynamin 2" was removed and described as "data not shown" in the main text because of the limitation of figure numbers.

Texts

All the changes in the text were highlighted in red.

Second decision letter

MS ID#: JOCES/2022/260827

MS TITLE: Pacsin 2-dependent N-cadherin internalization regulates the migration behaviour of malignant cancer cells

AUTHORS: Haymar Wint, Jianzhen Li, Tadashi Abe, Hiroshi Yamada, Takumi Higaki, Yasutomo Nasu, Masami Watanabe, Kohji Takei, and Tetsuya Takeda
ARTICLE TYPE: Research Article

We have now reached a decision on the above manuscript.

To see the reviewers' reports and a copy of this decision letter, please go to: https://submit-jcs.biologists.org and click on the 'Manuscripts with Decisions' queue in the Author Area. (Corresponding author only has access to reviews.)

As you will see, the reviewers gave favourable reports but raised some minor points that will require amendments to your manuscript. In particular, I think that reviewer 2 makes an important point for the new quantification you provided. I hope that you will be able to carry these out because I would like to be able to accept your paper once it is returned.

Please ensure that you clearly highlight all changes made in the revised manuscript. Please avoid using 'Tracked changes' in Word files as these are lost in PDF conversion.

I should be grateful if you would also provide a point-by-point response detailing how you have dealt with the points raised by the reviewers in the 'Response to Reviewers' box. Please attend to all of the reviewers' comments. If you do not agree with any of their criticisms or suggestions please explain clearly why this is so.

Reviewer 1

Advance summary and potential significance to field

I am reasonably satisfied with the new data in Fig. 6. Although I asked for an experiment that shows both WT and Pascin2 mutant junction localization in the same image/field of view/exposure time-- as a way to visually show increased enrichment of the mutant at junctions-- the modest

© 2023. Published by The Company of Biologists under the terms of the Creative Commons Attribution License (https://creativecommons.org/licenses/by/4.0/).

nature of the effect suggests that I would not see a large difference. The authors acknowledge this modest contributing role- and the data are now at least quantified-- so there is greater transparency on this issue. I am sufficiently satisfied with this revision.

Comments for the author

None

Reviewer 2

Advance summary and potential significance to field

Wint et al describe a role for pacsin2 in the organization of junctions in T25 bladder cancer cells. The findings are relevant for the (collective) migration of N-cadherin expressing cell types. The SH3 domain of Pacsin2 binds to the N-cadherin cytoplasmic domain, potentially explaining its unique effect on the trafficking of this classical cadherin.

Comments for the author

The authors have answered all my questions appropriately and have now discussed the possible differences between Pacsin2-mediated control over N-cadherin trafficking versus that of VE-cadherin or E-cadherin. By adding comparisons with other cell types the authors confirm that regulation of N-cadherin by pacsin2 is conserved.
• There is one new comment I have, based on the added quantifications in the revision: In figure 6G the authors show a quantification of GFP signal at cell cell junctions, concluding that GFP-tagged N-cadherin PA is slightly intenser compared to WT N-cadherin. However, the whole point of the experiment is that these cells have a higher number of adherens junctions (and thus more GFP signal).
To determine whether there is relatively more N-cadherin Pa, this quantification would need normalization compared to another junctional component, for instance alpha-catenin. This would also account for potential overexpression differences between the WT and PA mutant and the number of junctions per cell type.

Reviewer 3

Advance summary and potential significance to field

The authors identify Pacsin 2 playing a role in the regulation of collective cell migration. Silencing the expression of Pacsin 2 inhibits the endocytosis of N-cadherin, which can now stimulate cell-cell contacts and enhance the directionality of cell movement during wound healing.

Comments for the author

The authors have addressed all my concerns, as well as those of the other reviewers.
I have only two minor comments that can be addressed in writing:
1-The data showing that N-cadherin total levels do not change upon silencing pacsin 2 should be added.
2- In the discussion, the authors cite Hu et al, to indicate the CIP4, a protein similar in structure than Pacsin, inhibits the formation of invadopodia. However, there are other studies that show that CIP4 promotes invadopodia formation, including Pichot et al, 2010 (DOI: 10.1158/0008-5472.CAN-09-4149), and Kreider-Letterman 2023 (DOI: 10.1083/jcb.202207020). The difference in the study by Hu et al., is that they use active Src overexpressing breast cancer cells, vs. the the other studies use breast cancer cell lines. The overexpression of active Src, a key invadopodia regulator may introduce changes in invadopodia dynamics that couls account for the differences observed.

© 2023. Published by The Company of Biologists under the terms of the Creative Commons Attribution License (https://creativecommons.org/licenses/by/4.0/).

Second revision

Author response to reviewers' comments

Reviewer 1

Reviewer 1 Advance summary and potential significance to field
I am reasonably satisfied with the new data in Fig. 6. Although I asked for an experiment that shows both WT and Pascin2 mutant junction localization in the same image/field of view/exposure time-- as a way to visually show increased enrichment of the mutant at junctions-- the modest nature of the effect suggests that I would not see a large difference. The authors acknowledge this modest contributing role-and the data are now at least quantified-- so there is greater transparency on this issue. I am sufficiently satisfied with this revision.

Reviewer 1 Comments for the author
None

We thank Reviewer 1 for the favourable comments about the revision.

Reviewer 2

Reviewer 2 Advance summary and potential significance to field
Wint et al describe a role for pacsin2 in the organization of junctions in T25 bladder cancer cells. The findings are relevant for the (collective) migration of N-cadherin expressing cell types. The SH3 domain of Pacsin2 binds to the N-cadherin cytoplasmic domain, potentially explaining its unique effect on the trafficking of this classical cadherin.

Reviewer 2 Comments for the author
The authors have answered all my questions appropriately and have now discussed the possible differences between Pacsin2-mediated control over N-cadherin trafficking versus that of VE-cadherin or E-cadherin. By adding comparisons with other cell types the authors confirm that regulation of N-cadherin by pacsin2 is conserved.
•There is one new comment I have, based on the added quantifications in the revision: In figure 6G the authors show a quantification of GFP signal at cell cell junctions, concluding that GFP-tagged N-cadherin PA is slightly intenser compared to WT N-cadherin. However, the whole point of the experiment is that these cells have a higher number of adherens junctions (and thus more GFP signal). To determine whether there is relatively more N-cadherin Pa, this quantification would need normalization compared to another junctional component, for instance alpha-catenin. This would also account for potential overexpression differences between the WT and PA mutant and the number of junctions per cell type.

We thank Reviewer 2 for the valuable comments. Immunofluorescence microscopy showed the junctional accumulation of α-catenin was enhanced in T24 cells overexpressing PA mutant N-cadherin-GFP compared to in cells expressing WT N-cadherin-GFP with a statistical significance (new Fig. 6G and I). Importantly, relative intensities of N-cadherin-GFP normalized with α-catenin were equivalent between wild-type and PA mutants (Fig. 6, J), excluding the potential effects of overexpression differences between the WT and PA mutant.

Reviewer 3

Reviewer 3 Advance summary and potential significance to field

The authors identify Pacsin 2 playing a role in the regulation of collective cell migration. Silencing the expression of Pacsin 2 inhibits the endocytosis of N-cadherin, which can now stimulate cell-cell contacts and enhance the directionality of cell movement during wound healing.

Reviewer 3 Comments for the author

The authors have addressed all my concerns, as well as those of the other reviewers.
I have only two minor comments that can be addressed in writing:

© 2023. Published by The Company of Biologists under the terms of the Creative Commons Attribution License (https://creativecommons.org/licenses/by/4.0/).

1-The data showing that N-cadherin total levels do not change upon silencing pacsin 2 should be added.

It is now shown again in Figure S6 as requested.

2- In the discussion, the authors cite Hu et al, to indicate the CIP4, a protein similar in structure than Pacsin, inhibits the formation of invadopodia. However, there are other studies that show that CIP4 promotes invadopodia formation, including Pichot et al, 2010 (DOI: 10.1158/0008-5472.CAN-09-4149), and Kreider-Letterman 2023 (DOI: 10.1083/jcb.202207020). The difference in the study by Hu et al., is that they use active Src overexpressing breast cancer cells, vs. the the other studies use breast cancer cell lines. The overexpression of active Src, a key invadopodia regulator may introduce changes in invadopodia dynamics that couls account for the differences observed.

We thank Reviewer 3 for valuable information about CIP4 function in invadopodia formation. The suggested two papers, Pichot et al, 2010 (DOI: 10.1158/0008-5472.CAN-09-4149), and Kreider-Letterman 2023 (DOI: 10.1083/jcb.202207020), were described and cited in the discussion.

Third decision letter

MS ID#: JOCES/2022/260827

MS TITLE: Pacsin 2-dependent N-cadherin internalization regulates the migration behaviour of malignant cancer cells

AUTHORS: Haymar Wint, Jianzhen Li, Tadashi Abe, Hiroshi Yamada, Takumi Higaki, Yasutomo Nasu, Masami Watanabe, Kohji Takei, and Tetsuya Takeda
ARTICLE TYPE: Research Article

Thank you for sending your manuscript to Journal of Cell Science through Review Commons.

I am happy to tell you that your manuscript has been accepted for publication in Journal of Cell Science, pending standard ethics checks.

© 2023. Published by The Company of Biologists under the terms of the Creative Commons Attribution License (https://creativecommons.org/licenses/by/4.0/).
